# Causes and risk factors of death among people who inject drugs in Indonesia, Ukraine and Vietnam: findings from HPTN 074 randomized trial

**DOI:** 10.1186/s12879-023-08201-3

**Published:** 2023-05-11

**Authors:** Kostyantyn Dumchev, Xu Guo, Tran Viet Ha, Zubairi Djoerban, Oleksandr Zeziulin, Vivian F. Go, Riza Sarasvita, David S. Metzger, Carl A. Latkin, Scott M. Rose, Estelle Piwowar-Manning, Paul Richardson, Brett Hanscom, Kathryn E. Lancaster, William C. Miller, Irving F. Hoffman

**Affiliations:** 1 Ukrainian Institute On Public Health Policy, 5 Biloruska Str., Office 20, Kyiv, 04050 Ukraine; 2 Vaccine and Infectious Disease Division, Fred Hutchinson Cancer Research Center, Seattle, WA USA; 3 Dept. of Health Behavior, Gilings School of Global Public Health, University of North Carolina at Chapel Hill, Chapel Hill, NC USA; 4 Depts. of Hematology, Medical Oncology, and Medicine, Univ. of Indonesia/ Cipto Mangunkusumo Hospital, Jakarta, Indonesia; 5 National Narcotics Board, Jakarta, Indonesia; 6 HIV Prevention Research Division, Department of Psychiatry, School of Medicine, University of Pennsylvania, Philadelphia, Pennsylvania USA; 7 Dept. of Health, Behavior and Society, Bloomberg School of Public Health, Johns Hopkins University, Baltimore, Maryland USA; 8 Science Facilitation Department, Durham, North Carolina FHI 360 USA; 9 Dept. of Pathology, School of Medicine, Johns Hopkins University, Baltimore, MD USA; 10 Division of Epidemiology, College of Public Health, The Ohio State University, Columbus, OH USA; 11 Dept. of Medicine, School of Medicine, University of North Carolina at Chapel Hill, Chapel Hill, NC USA

**Keywords:** HIV, People who inject drugs, Mortality, HPTN 074, Prevention trial, Opioid agonist treatment

## Abstract

**Introduction:**

The HIV Prevention Trials Network (HPTN) 074 study demonstrated a positive effect of an integrated systems navigation and psychosocial counseling intervention on HIV treatment initiation, viral suppression, medication assisted treatment (MAT) enrollment, and risk of death among people who inject drugs (PWID). In this sub-study, we analyzed the incidence, causes, and predictors of death among HIV-infected and uninfected participants.

**Methods:**

The HPTN 074 randomized clinical trial was conducted in Indonesia, Ukraine, and Vietnam. HIV-infected PWID with unsuppressed viral load (indexes) were recruited together with at least one of their HIV-negative injection partners. Indexes were randomized in a 1:3 ratio to the intervention or standard of care.

**Results:**

The trial enrolled 502 index and 806 partner participants. Overall, 13% (66/502) of indexes and 3% (19/806) of partners died during follow-up (crude mortality rates 10.4 [95% CI 8.1–13.3] and 2.1 [1.3–3.3], respectively). These mortality rates were for indexes nearly 30 times and for partners 6 times higher than expected in a population of the same country, age, and gender (standardized mortality ratios 30.7 [23.7–39.0] and 5.8 [3.5–9.1], respectively). HIV-related causes, including a recent CD4 < 200 cells/μL, accounted for 50% of deaths among indexes. Among partners, medical conditions were the most common cause of death (47%). In the multivariable Cox model, the mortality among indexes was associated with sex (male versus female aHR = 4.2 [1.5–17.9]), CD4 count (≥ 200 versus < 200 cells/μL aHR = 0.3 [0.2–0.5]), depression (moderate-to-severe versus no/mild aHR = 2.6 [1.2–5.0]) and study arm (intervention versus control aHR = 0.4 [0.2–0.9]). Among partners, the study arm of the index remained the only significant predictor (intervention versus control aHR = 0.2 [0.0–0.9]) while controlling for the effect of MAT (never versus ever receiving MAT aHR = 2.4 [0.9–7.4]).

**Conclusions:**

The results confirm that both HIV-infected and uninfected PWID remain at a starkly elevated risk of death compared to general population. Mortality related to HIV and other causes can be significantly reduced by scaling-up ART and MAT. Access to these life-saving treatments can be effectively improved by flexible integrated interventions, such as the one developed and tested in HPTN 074.

**Supplementary Information:**

The online version contains supplementary material available at 10.1186/s12879-023-08201-3.

## Introduction

People who inject drugs (PWID) are at higher risk of death compared to their non-drug using peers. The all-cause mortality rate in a global systematic review of cohort studies among PWID was 2.9 per 100 person-years (PY), which is nearly 15 times higher than expected in an age-adjusted population [1]. A more recent review reported a pooled mortality rate of 2.7/100PY among people injecting opioids [2]. The main causes of premature death in PWID are opioid overdose and AIDS [1–3]. Other causes of death are common as well, including but not limited to accidents [4], suicide [5, 6], and other communicable diseases [2, 7]. HIV infection contributes to increased mortality from both AIDS-related and drug-related [8, 9] causes.

Widespread scale-up of antiretroviral therapy (ART) has led to a dramatic decline in all-cause mortality among people living with HIV (PLWH) worldwide. Unfortunately, these benefits have not been fully extended to PWID due to multiple structural, psychosocial, and other barriers, including lack of treatment for opioid dependence [10].

Effective treatment of opioid dependence leads to reduction in drug use and increased engagement and adherence to other life-saving treatments, radically reducing overdose and AIDS-related mortality in short- and long-term [11]. Opioid agonist treatment (OAT), the most effective method to treat opioid dependence, is implemented in only about half of the countries with evidence of drug use [10]. In places where OAT is available, coverage remains suboptimal, especially in low- and middle-income countries.

Numerous interventions have been developed and tested to improve initiation and retention in HIV care and OAT for PWID. Many were shown to have the desired effect in observational or experimental designs [12–14], but only a few have demonstrated an indirect impact on mortality [15, 16].

The HIV Prevention Trials Network (HPTN) 074 study was designed to evaluate the feasibility and efficacy of an integrated, scalable intervention aimed at improving ART uptake and viral suppression [17]. The multi-site, two-arm, randomized vanguard trial enrolled HIV-infected indexes who were assigned to either the integrated intervention or the standard of care arm (SoC) in a 1:3 ratio. HIV-negative injection partners were also enrolled and followed. The intervention combined systems navigation with psychosocial counseling. The intervention was shown to be effective in increasing ART use, viral suppression, and OAT use, although the results varied by site [18]. Importantly, a reduction in mortality was also seen in the experimental arm as compared to the standard of care. This reduction was statistically significant both in HIV-infected indexes and their HIV-negative injection partners.

In this sub-study, we analyze the incidence of death in a variety of demographic and clinical subgroups, summarize the causes of death in this cohort, and explore predictors of mortality among PWID enrolled in the HPTN 074 trial.

## Methods

### Population and setting

The study was conducted at three sites (Jakarta, Indonesia; Kyiv, Ukraine; and Thai Nguyen, Vietnam). The sites were selected based on estimated high HIV incidence and prevalence among PWID. Participants were recruited in a city hospital (Jakarta), two district health centers (Thai Nguyen), and a community-based harm reduction program (Kyiv). The main recruitment methods were community outreach and peer referral. Study enrollment occurred between April of 2015 and June of 2016 and follow-up in the main phase continued until June 2017.

All participants were required to be between 18–45 years of age (the maximum allowed age increased to 60 years after 8 months into the study), active injectors (defined as have injected at least 12 times in the past 3 months), planning to stay in the study area for one year, and able and willing to provide informed consent. Additional criteria for index participants included confirmed HIV infection, HIV viral load ≥ 1,000 copies/mL, CD4 count > 50 cells/μL, and ability to recruit at least one HIV-negative injection partner. Partners had to have HIV-negative test results at screening and have a confirmed relationship with the index (verified by matching the physical description).

### Data collection

The study procedures and data collection methods for the main outcomes are presented in detail in the main study report [18]. In brief, index candidates who matched all eligibility criteria and gave informed consent were enrolled in the study and randomized through a secure web portal either to the standard care or intervention groups. Each index was asked to recruit up to five uninfected injection partners. In the main study phase, both index and partner participants completed an interviewer-administered questionnaire (S1-S4 supporting information files) and provided biological samples at screening, enrollment, one month after enrollment, and at quarterly visits until up to 24 months past enrollment. The index-specific questionnaire included the standard PHQ-9 instrument for depression [19], and questions related to social support and stigma. PHQ-9 scores of 1 to 9 were categorized as “no/mild” depression, and scores of 10 or above as “moderate/severe”. Self-reported injection and non-injection drug use in the past three months was summarized using the following yes/no variables: opioids (heroin, opium, dezomorphine, home-made opioids, codeine, illegal methadone, or buprenorphine), stimulants (amphetamines, methamphetamines, cocaine, spices), other drugs (ketamine, benzodiazepines, dimedrol), polydrug use (any combination of the previous groups), no drug use (no drug use reported). Neither questionnaire included questions about non-fatal overdose experience.

All instances of death were systematically investigated by the local study teams. After becoming aware of a possible death case, study personnel contacted relatives, peers or medical staff to confirm the death and ascertain the date and the cause of death. All case report forms were independently assessed by two independent physicians to verify and categorize the cause; discrepancies were resolved by a third physician.

### Statistical analysis

Crude mortality rates per 100 person years (CMR) were calculated based upon person-time between study enrollment and either the date of death or last follow-up visit. For time-varying covariates, the amount of person time in each category was determined based on the covariate value at the end of each follow-up interval. Exact confidence intervals for crude mortality rates were calculated using the Poisson distribution.

Standardized mortality ratios (SMR) were calculated as the observed number of deaths in each subgroup divided by the number of deaths that would be expected during the registered person-time. The mean annual numbers of expected deaths were calculated using the data from the Global Burden of Disease Study, and computed separately for males and females, each 5-year age category, and each country [20]. Deaths in our study population were disaggregated into matching age and gender groups before calculations were performed. The 95% confidence intervals of SMR were calculated according to a standard formula [21].

We used Cox proportional hazard models to analyze predictors of mortality. Unadjusted models included each variable of interest on its own, and adjusted models included the variable of interest in addition to age, gender, site, and study arm. Multivariable models included at the first step all variables significant in the adjusted cox models with a conservative cutoff point of 0.1 and then stepwise deletion was used to remove non-significant variables from the final models. All covariates collected after baseline (e.g. ART use, CD4, viral load, OAT, depression, substance use, stigma) were analyzed as time-varying covariates in the model. Missing data in the longitudinal sequence was imputed from either the most recent visit with non-missing data (for categorical variables), or the mean value between the previous and following visits (continuous variables). Analyses were performed using SAS Version 9.4.

Due to substantial differences in mortality rates and causes between HIV-positive indexes and HIV-negative partners, the two groups were analyzed separately.

This trial was registered on ClinicalTrials.gov, NCT02935296, on 17/10/2016. The study protocol is available at https://www.hptn.org/research/studies/hptn074.

## Results

The trial enrolled 502 index (194 [39%] in Vietnam, 187 [37%] in Ukraine, and 121 [24%]) in Indonesia) and 806 partner participants (305 [38%] in Vietnam, 318 [39%] in Ukraine, and 183 [23%]) in Indonesia). A majority were men (87%, 1143/1308); most women (77%, 127/165) enrolled at the Ukrainian site. The median age at enrollment was 34 years (IQR: 30, 38), with no significant difference by study group. Participants in Indonesia were slightly younger compared to Ukraine and Vietnam (median age 32, 34, 35, respectively). A majority reported opioid use in the past three months before baseline (99%, 1299/1308). The median lifetime duration of drug injecting was 13 years (range 0–37) and the median number of injecting days per month was 25 (range 0–31). Detailed baseline characteristics of the study cohort are presented in a previous report [22].

Median follow-up time was 1.2 years (IQR: 0.9–1.5); 3.8% of indexes (9/502) and 6.6% of partners (29/806) were lost to follow-up, and their survival status could not be verified.

### Mortality rates

Among indexes, who were HIV-positive and had viral load > 1000cp/ml at baseline, 13% (66/502) died during the follow-up (CMR, 10.4 [95% CI 8.1–13.3], Table 1). This rate was much higher than expected in a population with the same age structure (SMR, 30.7 [23.7–39.0]). The rates differed notably between the study sites. The CMR among indexes was lowest in Ukraine, 6.5 [3.7–10.5] followed by a 1.5 times higher rate in Vietnam (9.9 [6.3–14.7]), and a nearly 3 times higher rate in Indonesia (18.2 [11.9–26.7]). The risk of mortality was starkly elevated in comparison to the general population in all three countries (SMR, 13.2 [7.5–21.4] in Ukraine, 35.9 [23.0–53.5] in Vietnam, and 96.6 [63.1–141.6] in Indonesia). Across the subcategories, the highest mortality was among participants who met diagnostic criteria for AIDS, i.e. had a CD4 count of < 200 cells/μL (CMR, 20.8 [14.8–28.4]; SMR, 60.6 [43.1–82.8]).

**Table 1:** Crude Mortality Rates (CMR) and Standardized Mortality Ratios (SMR) for index participants, by Key Strata

Variable	No. of Deaths	Person-Years	CMR per 100PY [95% CI]^a^	SMR [95% CI]^b^
Overall	66	633.01	10.4 [8.1, 13.3]	30.7 [23.7, 39.0]
Gender				
Female	3	106.90	2.8 [0.6, 8.2]	17.2 [3.6, 50.4]
Male	63	526.11	12.0 [9.2, 15.3]	31.9 [24.5, 40.8]
Study site				
Indonesia	26	142.61	18.2 [11.9, 26.7]	96.6 [63.1, 142]
Ukraine	16	247.84	6.5 [3.7, 10.5]	13.2 [7.5, 21.4]
Vietnam	24	242.56	9.9 [6.3, 14.7]	35.9 [23.0, 53.5]
Study site by Gender				
Indonesia – Female	0	13.13	0.0 [0.0, 28.1]	0.0 [0.0, 212.8]
Indonesia – Male	26	129.48	20.1 [13.1, 29.4]	103 [67.5, 151.3]
Ukraine – Female	3	92.16	3.3 [0.7, 9.5]	19.3 [4.0, 56.5]
Ukraine – Male	13	155.68	8.4 [4.5, 14.3]	12.3 [6.5, 21.0]
Vietnam – Female	0	1.60	0.0 [0.0, 230.2]	0.0 [0.0, 2348.9]
Vietnam – Male	24	240.95	10.0 [6.4, 14.8]	36.0 [23.1, 53.6]
Study arm				
SOC	57	472.22	12.1 [9.1, 15.6]	35.6 [27.0, 46.1]
Intervention	9	160.79	5.6 [2.6, 10.6]	16.3 [7.5, 31.0]
Alcohol use in the past 3 months				
Non-hazardous alcohol use	53	431.75	12.3 [9.2, 16.1]	40.0 [30.0, 52.3]
Hazardous alcohol use	13	201.26	6.5 [3.4, 11.1]	15.7 [8.4, 26.9]
Injected opioids in the past 3 months				
No	15	121.29	12.4 [6.9, 20.4]	53.7 [30.1, 88.6]
Yes	51	511.72	10.0 [7.4, 13.1]	27.2 [20.3, 35.8]
Injected stimulants in the past 3 months				
No	62	550.59	11.3 [8.6, 14.4]	35.1 [26.9, 45.0]
Yes	4	82.42	4.9 [1.3, 12.4]	10.4 [2.8, 26.5]
Injected other drugs in the past 3 months				
No	61	500.08	12.2 [9.3, 15.7]	40.7 [31.1, 52.2]
Yes	5	132.93	3.8 [1.2, 8.8]	7.7 [2.5, 17.9]
No injecting drug use in the past 3 months				
No	52	516.29	10.1 [7.5, 13.2]	27.5 [20.6, 36.1]
Yes	14	116.72	12.0 [6.6, 20.1]	53.5 [29.3, 89.8]
Injecting polydrug use in the past 3 months				
No	59	456.33	12.9 [9.8, 16.7]	45.1 [34.4, 58.2]
Yes	7	176.67	4.0 [1.6, 8.2]	8.3 [3.3, 17.1]
Non-injected opioid use in the past 3 months				
No	64	596.37	10.7 [8.3, 13.7]	31.2 [24.0, 39.8]
Yes	2	36.63	5.5 [0.7, 19.7]	20.5 [2.5, 74.1]
Non-injected stimulant use in the past 3 months				
No	43	461.43	9.3 [6.7, 12.6]	25.1 [18.2, 33.8]
Yes	23	171.57	13.4 [8.5, 20.1]	52.4 [33.2, 78.6]
Non-injecting other drug use in the past 3 months				
No	35	280.60	12.5 [8.7, 17.4]	40.6 [28.3, 56.5]
Yes	31	352.41	8.8 [6.0, 12.5]	24.0 [16.3, 34.1]
No non-injecting drug use in the past 3 months				
No	41	436.69	9.4 [6.7, 12.7]	27.6 [19.8, 37.5]
Yes	25	196.31	12.7 [8.2, 18.8]	37.5 [24.2, 55.3]
Non-injecting polydrug use in the past 3 months				
No	52	511.98	10.2 [7.6, 13.3]	28.6 [21.4, 37.5]
Yes	14	121.03	11.6 [6.3, 19.4]	42.0 [23.0, 70.5]
OAT status				
ever used	33	349.54	9.4 [6.5, 13.3]	28.2 [19.4, 39.6]
OAT naïve	33	283.46	11.6 [8.0, 16.4]	33.7 [23.2, 47.3]
ART status				
ART naïve	38	253.26	15.0 [10.6, 20.6]	40.7 [28.8, 55.9]
previously on ART	3	53.80	5.6 [1.2, 16.3]	18.8 [3.9, 55.0]
current on ART	25	325.95	7.7 [5.0, 11.3]	23.6 [15.3, 34.9]
Depression				
no/mild	51	512.93	9.9 [7.4, 13.1]	31.6 [23.5, 41.5]
moderate/severe	15	120.08	12.5 [7.0, 20.6]	28.0 [15.7, 46.2]
Internalized stigma				
No	27	270.31	10.0 [6.6, 14.5]	26.9 [17.7, 39.2]
Yes	39	362.70	10.8 [7.7, 14.7]	34.0 [24.2, 46.4]
Experienced stigma				
No	51	495.83	10.3 [7.7, 13.5]	30.7 [22.8, 40.3]
Yes	15	137.18	10.9 [6.1, 18.0]	30.8 [17.2, 50.8]
Anticipated stigma				
No	29	234.27	12.4 [8.3, 17.8]	34.1 [22.9, 49.0]
Yes	37	398.74	9.3 [6.5, 12.8]	28.4 [20.0, 39.2]
Total stigma				
No	18	147.21	12.2 [7.3, 19.3]	32.6 [19.3, 51.5]
Yes	48	485.80	9.9 [7.3, 13.1]	30.0 [22.1, 39.8]
Viral suppression				
≥ 40 copies/mL	60	468.00	12.8 [9.8, 16.5]	38.9 [29.7, 50.0]
< 40 copies/mL	6	165.01	3.6 [1.3, 7.9]	9.9 [3.6, 21.5]
CD4 count				
< 200 cells/μL	39	187.63	20.8 [14.8, 28.4]	60.6 [43.1, 82.8]
≥ 200 cells/μL	27	445.38	6.1 [4.0, 8.8]	17.9 [11.8, 26.1]

^a^ Based upon person-time between study enrollment and either the date of death or last follow-up visit. For time-varying covariates, the amount of person time was determined based on the value at the end of each follow-up interval. Exact confidence intervals were calculated using the Poisson distribution

^b^ Calculated as the observed number of deaths divided by the number of deaths that would be expected according to the data from the Global Burden of Disease Study, separately for males and females, each 5-year age category, and each country

Among partners, who were HIV-negative at baseline, 3% (19/806) died during the follow-up (CMR, 2.1 [1.3–3.3], Table 2). This is nearly six times higher than expected in the population with the same age structure (SMR, 5.8 [3.5–9.1]). Unlike in indexes, the lowest mortality among partners was in Vietnam (CMR, 1.1 [0.3–2.9]), followed by Indonesia (1.5 [0.3–4.5] and then Ukraine (3.3 [1.7–5.8]). These rates were 4.2, 6.2, and 7.9 times higher than expected in the general population, respectively. Across the subcategories, the highest mortality was among those who reported injecting drugs other than opioids or stimulants in the past 3 months (CMR, 3.4 [1.3–7.4]; SMR, 6.8 [2.5–14.7]), injected multiple drugs (CMR, 3.2 [1.4–6.4]; SMR, 6.5 [2.8–12.8]), and were OAT naïve (CMR, 3.1 [1.7–5.2]; SMR, 8.4 [4.6–14.1]).

**Table 2:** Crude Mortality Rates (CMR) and Standardized Mortality Ratios (SMR) for partner participants, by Key Strata

Variable	No. of Deaths	Person-Years	CMR per 100PY [95% CI]^a^	SMR [95% CI]^b^
Overall	19	910.88	2.1 [1.3, 3.3]	5.8 [3.5, 9.1]
Gender				
Female	2	110.15	1.8 [0.2, 6.6]	11.0 [1.3, 39.6]
Male	17	800.73	2.1 [1.2, 3.4]	5.5 [3.2, 8.9]
Study site				
Indonesia	3	193.87	1.5 [0.3, 4.5]	7.9 [1.6, 23.0]
Ukraine	12	364.21	3.3 [1.7, 5.8]	6.2 [3.2, 10.9]
Vietnam	4	352.80	1.1 [0.3, 2.9]	4.2 [1.1, 10.7]
Study site by Gender				
Indonesia – Female	0	19.13	0.0 [0.0, 19.3]	0.0 [0.0, 143.0]
Indonesia – Male	3	174.74	1.7 [0.4, 5.0]	8.4 [1.7, 24.7]
Ukraine – Female	2	81.68	2.4 [0.3, 8.9]	13.4 [1.6, 48.6]
Ukraine – Male	10	282.53	3.5 [1.7, 6.5]	5.6 [2.7, 10.4]
Vietnam – Female	0	9.34	0.0 [0.0, 39.5]	0.0 [0.0, 473.6]
Vietnam – Male	4	343.46	1.2 [0.3, 3.0]	4.2 [1.2, 10.8]
Study arm				
SOC	18	694.68	2.6 [1.5, 4.1]	7.3 [4.3, 11.6]
Intervention	1	216.20	0.5 [0.0, 2.6]	1.2 [0.0, 7.0]
Alcohol use in the past 3 months				
Non-hazardous alcohol use	10	604.48	1.7 [0.8, 3.0]	5.0 [2.4, 9.1]
Hazardous alcohol use	9	306.40	2.9 [1.3, 5.6]	7.2 [3.3, 13.7]
Injecting opioids in the past 3 months				
No	0	146.97	0.0 [0.0, 2.5]	0.0 [0.0, 9.6]
Yes	19	763.91	2.5 [1.5, 3.9]	6.6 [4.0, 10.3]
Injecting stimulants in the past 3 months				
No	16	770.29	2.1 [1.2, 3.4]	6.2 [3.5, 10.0]
Yes	3	140.59	2.1 [0.4, 6.2]	4.5 [0.9, 13.1]
Injecting other drugs in the past 3 months				
No	13	734.08	1.8 [0.9, 3.0]	5.5 [2.9, 9.4]
Yes	6	176.80	3.4 [1.3, 7.4]	6.8 [2.5, 14.7]
No injecting drug use in the past 3 months				
No	19	774.97	2.5 [1.5, 3.8]	6.5 [3.9, 10.2]
Yes	0	135.91	0.0 [0.0, 2.7]	0.0 [0.0, 11.0]
Injecting Polydrug use in the past 3 months				
No	11	663.46	1.7 [0.8, 3.0]	5.4 [2.7, 9.7]
Yes	8	247.42	3.2 [1.4, 6.4]	6.5 [2.8, 12.8]
Non-injecting opioids use in the past 3 months				
No	18	850.93	2.1 [1.3, 3.3]	5.9 [3.5, 9.3]
Yes	1	59.95	1.7 [0.0, 9.3]	5.3 [0.1, 29.4]
Non-injecting stimulants use in the past 3 months				
No	14	631.86	2.2 [1.2, 3.7]	5.7 [3.1, 9.6]
Yes	5	279.03	1.8 [0.6, 4.2]	6.3 [2.0, 14.6]
Non-injecting other drug use in the past 3 months				
No	8	391.92	2.0 [0.9, 4.0]	5.9 [2.5, 11.6]
Yes	11	518.97	2.1 [1.1, 3.8]	5.8 [2.9, 10.4]
No non-injecting drug use in the past 3 months				
No	13	637.99	2.0 [1.1, 3.5]	5.8 [3.1, 9.9]
Yes	6	272.89	2.2 [0.8, 4.8]	6.0 [2.2, 13.0]
Non-injecting polydrug use in the past 3 months				
No	15	696.48	2.2 [1.2, 3.6]	5.7 [3.2, 9.4]
Yes	4	214.40	1.9 [0.5, 4.8]	6.4 [1.8, 16.5]
OAT status				
ever used	5	460.78	1.1 [0.4, 2.5]	3.1 [1.0, 7.3]
OAT naïve	14	450.10	3.1 [1.7, 5.2]	8.4 [4.6, 14.1]

^a^ Based upon person-time between study enrollment and either the date of death or last follow-up visit. For time-varying covariates, the amount of person time was determined based on the value at the end of each follow-up interval. Exact confidence intervals were calculated using the Poisson distribution

^b^ Calculated as the observed number of deaths divided by the number of deaths that would be expected according to the data from the Global Burden of Disease Study, separately for males and females, each 5-year age category, and each country

### Causes of death

The leading cause of death among HIV-infected index participants was HIV-related opportunistic infections (17/66, 25.8%), primarily tuberculosis (13/66, 19.7%; Table 3). An additional 16 (24.2%) deaths with unknown cause were among participants who had AIDS according to immunologic criteria (most-recent CD4 < 200 cells/μL). Medical, non-HIV related causes such as lung, liver, or kidney disease, led to 14 (21.2%) deaths. Relatively few deaths with known cause were related to overdose (2), suicide (2), and trauma (3). Twelve deaths occurred among people with a CD4 count of 200 cells/μL or higher where the cause could not be identified. Most deaths in index participants (57/66, 86.4%) occurred in the SoC arm, including all accidental deaths.

**Table 3:** Causes of death among indexes by study arm, ART receipt at last visit and by site

	By study arm		By site			
	SOC	Intervention	Indonesia	Ukraine	Vietnam	Overall
Total Deaths	57	9	26	16	24	66
HIV^1^ (TB)	12 (10) 21% (18%)	5 (3) 56% (33%)	12 (9) 47% (35%)	4 (3) 25% (19%)	1 (1) 4% (4%)	17 (13) 26% (20%)
Medical, Non-HIV^2^	13 23%	1 11%	4 15%	5 31%	5 20%	14 21%
Overdose	2 3%	-	-	1 6%	1 4%	2 3%
Other^3^	5 9%	-	1 4%	-	4 17%	5 8%
Unknown, CD4 < 200 cells/μL	16 28%	-	5 19%	2 13%	9 38%	16 24%
Unknown, CD4 ≥ 200 cells/μL	9 16%	3 33%	4 15%	4 25%	4 17%	12 18%

^1^ “HIV” category includes pulmonary and extrapulmonary tuberculosis and other opportunistic infections, such as candidiasis and toxoplasmosis

^2^ “Medical, Non-HIV” category includes liver, kidney, or lung diseases

^3^ “Other” category includes suicide, trauma related to violence, and accidental trauma

Deaths among the HIV uninfected partner participants were primarily caused by health conditions (9/19, 47.4%), including tuberculosis (3/19, 15.8%; Table 4). In about 20% of cases, the cause was not known. Three deaths were caused by overdose, and another three were due to suicide and trauma. All except one death occurred in the SoC arm.

**Table 4:** Causes of death among partners by study arm and by site

	By study arm		By site			
	SOC	Intervention	Indonesia	Ukraine	Vietnam	Overall
Total Deaths	18	1	3	12	4	19
Medical^1^ (TB)	8 (3) 44% (17%)	1 (0) 100% (0%)	3 (2) 100% (67%)	6 (1) 50% (8%)	-	9 (3) 47% (16%)
Overdose	3 17%	-	-	2 17%	1 25%	3 16%
Other^2^	3 17%	-	-	-	3 75%	3 16%
Unknown	4 22%	-	-	4 33%	-	4 21%

^1^ “Medical” category includes tuberculosis, other lung diseases, liver diseases, thromboembolia

^2^ “Other” category includes suicide, trauma related to violence, and accidental trauma

### Predictors of mortality

Multiple variables were associated with the risk of death among index participants (Tables 5 and 6). Participation in the intervention compared to SoC was associated with lower risk of death in the unadjusted, adjusted, and final models (aHR = 0.4 [0.2–0.9]). The risk was also lower at the Ukraine and Vietnam sites compared to Indonesia in the adjusted models (aHR = 0.4 [0.2–0.9]; HR = 0.5 [0.3–0.9], respectively), but in the multivariable model the difference remained significant only for Vietnam. Among the well-known clinical predictors of survival in HIV patients, viral suppression and higher CD4 count were associated with a lower risk of death (HR = 0.4 [0.2–1.0]; HR = 0.3 [0.2–0.5], respectively), while ART receipt itself did not have any effect (HR = 0.8 [0.5–1.3]). In the multivariable model, controlling for age, gender, site, and study arm, viral suppression became not significant, while CD4 count remained in the model. Self-reported recent injecting of drugs (opioids, other, polydrug use) was also associated with lower mortality, both in unadjusted and adjusted models, with polydrug use remaining significant in the final model. Self-report of not using any injecting or non-injecting drug use in the past 3 months was associated with increased risk of death in unadjusted and adjusted models, respectively, but both lost significance in the multivariable model. In addition, the final model included male gender (aHR = 4.2 [1.5–17.9]), anticipated stigma (aHR = 0.6 [0.4–1.0]), and presence of moderate to severe depression (aHR = 2.6 [1.2–5.0]) in the model.

**Table 5:** Unadjusted and adjusted Hazard Ratios for all-cause mortality in index participants

	Unadjusted Cox Model			Adjusted Cox Model^**1**^			Multivariable Cox Model^**2**^		
	HR	95% CI^**3**^	*p*-value^**4**^	aHR	95% CI^**3**^	*p*-value^**4**^	aHR	95% CI^**3**^	*p*-value^**4**^
Age	1.00	0.96, 1.05	0.981	1.01	0.96, 1.06	0.670	1.00	0.95, 1.05	0.980
Gender			0.003			0.012			0.005
Female	REF			REF			REF		
Male	4.13	1.53,16.92		3.66	1.29,15.39		4.23	1.46,17.94	
Study site			0.006			0.028			0.082
Indonesia	REF			REF			REF		
Ukraine	0.37	0.19, 0.68		0.46	0.23, 0.86		0.62	0.26, 1.40	
Vietnam	0.56	0.32, 0.98		0.52	0.29, 0.91		0.50	0.27, 0.92	
Study arm			0.019			0.015			0.013
SOC	REF			REF			REF		
Intervention	0.46	0.21, 0.89		0.45	0.21, 0.87		0.44	0.20, 0.85	
Alcohol use in the past 3 months			0.019			0.012			
Non Hazardous alcohol use	REF			REF					
Hazardous alcohol use	0.46	0.21, 0.89		0.44	0.20, 0.84				
Injecting opioids in the past 3 months			0.009			0.049			
No	REF			REF					
Yes	0.41	0.22, 0.79		0.50	0.26, 1.00				
Injecting stimulants in the past 3 months			0.023			0.160			
No	REF			REF					
Yes	0.36	0.11, 0.88		0.49	0.14, 1.29				
Injecting other drugs in the past 3 months			0.005			0.038			
No	REF			REF					
Yes	0.32	0.11, 0.73		0.35	0.11, 0.95				
No injecting drug use in the past 3 months			0.013			0.077			
No	REF			REF					
Yes	2.39	1.21, 4.48		1.91	0.93, 3.76				
Injecting polydrug use in the past 3 months			< 0.001			0.006			0.005
No	REF			REF			REF		
Yes	0.29	0.12, 0.60		0.29	0.11, 0.71		0.28	0.10, 0.69	
# of days of injecting drugs use in the past month	1.00	0.98, 1.02	0.851	1.01	0.99, 1.03	0.381			
Non-injecting opioids use in the past 3 months			0.253			0.271			
No	REF			REF					
Yes	0.48	0.08, 1.54		0.49	0.08, 1.61				
Non-injecting stimulants use in the past 3 months			0.289			0.755			
No	REF			REF					
Yes	1.32	0.78, 2.17		0.91	0.50, 1.62				
Non-injecting other drug use in the past 3 months			0.206			0.124			
No	REF			REF					
Yes	0.73	0.45, 1.19		0.67	0.40, 1.12				
No non-injecting drug use in the past 3 months			0.230			0.040			0.088
No	REF			REF			REF		
Yes	1.36	0.82, 2.23		1.78	1.03, 3.05		1.62	0.93, 2.78	
Non-injecting polydrug use in the past 3 months			0.809			0.484			
No	REF			REF					
Yes	1.08	0.57, 1.89		0.80	0.41, 1.47				
OAT status			0.844			0.912			
Ever used	REF			REF					
OAT naive	1.05	0.64, 1.71		0.97	0.59, 1.60				
ART status			0.315			0.327			
ART naive	REF			REF					
previously on ART	0.47	0.11, 1.31		0.47	0.11, 1.32				
current on ART	0.78	0.45, 1.32		0.78	0.44, 1.36				
Depression			0.837			0.052			0.012
no/mild	REF			REF			REF		
moderate/severe	1.06	0.58, 1.85		2.06	0.99, 4.11		2.55	1.24, 5.01	
Internalized stigma			0.725			0.675			
No	REF			REF					
Yes	0.91	0.56, 1.51		0.90	0.54, 1.50				
Experienced stigma			0.490			0.423			
No	REF			REF					
Yes	0.82	0.44, 1.43		0.79	0.42, 1.39				
Anticipated stigma			0.125			0.097			0.043
No	REF			REF			REF		
Yes	0.68	0.42, 1.11		0.65	0.40, 1.08		0.59	0.36, 0.98	
Total stigma			0.171			0.143			
No	REF			REF					
Yes	0.67	0.40, 1.19		0.65	0.38, 1.16				
Viral suppression			0.036			0.084			
≥ 40 copies/mL	REF			REF					
< 40 copies/mL	0.43	0.16, 0.95		0.49	0.18, 1.09				
CD4 count			< 0.001			< 0.001			< 0.001
< 200 cells/μL	REF			REF			REF		
≥ 200 cells/μL	0.28	0.17, 0.46		0.31	0.19, 0.50		0.29	0.17, 0.47	

^1^ Age, gender, site, arm was adjusted for in the Adjusted Cox Models

^2^ Those significant in the adjusted cox model were selected in this model with conservative cutoff point of 0.1. adjusting for age, gender, site, arm, only those with *p*-value < 0.1 will be left in the final model

^3^ Profile likelihood 95% confidence interval

^4^ Logrank type3 *p*-value

**Table 6:** Unadjusted and adjusted Hazard Ratios for all-cause mortality in partner participants

	**Unadjusted Cox Model**			**Adjusted Cox Model**^**1**^			**Multivariable Cox Model**^**2**^		
	**HR**	**95% CI**^**3**^	***p*-value**^**4**^	**aHR**	**95% CI**^**3**^	***p*-value**^**4**^	**aHR**	**95% CI**^**3**^	***p*-value**^**4**^
Age	1.04	0.97, 1.11	0.264	1.04	0.97, 1.12	0.211	1.04	0.97, 1.11	0.230
Gender			0.801			0.409			0.522
Female	REF			REF			REF		
Male	1.20	0.34, 7.58		1.80	0.50,11.49		1.58	0.44,10.13	
Study site			0.173			0.130			0.061
Indonesia	REF			REF			REF		
Ukraine	1.78	0.57, 7.81		1.57	0.49, 7.02		2.02	0.63, 8.97	
Vietnam	0.66	0.15, 3.35		0.52	0.11, 2.70		0.56	0.12, 2.90	
Study arm			0.026			0.023			0.029
SOC	REF			REF			REF		
Intervention	0.17	0.01, 0.84		0.17	0.01, 0.82		0.18	0.01, 0.86	
Alcohol use in the past 3 months			0.225			0.393			
Non Hazardous alcohol use	REF			REF					
Hazardous alcohol use	1.76	0.70, 4.36		1.51	0.58, 3.94				
Injecting opioids in the past 3 months^5^			0.071			0.103			
No	REF			REF					
Yes	3.64	0.48,466.7		3.06	0.37,399.1				
Injecting stimulants in the past 3 months			0.790			0.239			
No	REF			REF					
Yes	0.85	0.20, 2.55		0.48	0.11, 1.58				
Injecting other drugs in the past 3 months			0.294			0.933			
No	REF			REF					
Yes	1.71	0.60, 4.34		0.95	0.30, 3.00				
No injecting drug use in the past 3 months^5^			0.090			0.149			
No	REF			REF					
Yes	0.32	0.00, 2.48		0.41	0.00, 3.73				
Injecting polydrug use in the past 3 months			0.320			0.635			
No	REF			REF					
Yes	1.60	0.62, 3.95		0.75	0.24, 2.61				
# of days of injecting drugs use in the past month	1.03	0.99, 1.08	0.134	1.03	0.98, 1.08	0.277			
Non-injecting opioids use in the past 3 months			0.754			0.989			
No	REF			REF					
Yes	0.74	0.04, 3.57		0.99	0.05, 5.18				
Non-injecting stimulants use in the past 3 months			0.700			0.753			
No	REF			REF					
Yes	0.82	0.26, 2.15		0.84	0.25, 2.37				
Non-injecting other drug use in the past 3 months			0.857			0.884			
No	REF			REF					
Yes	1.09	0.44, 2.81		0.93	0.37, 2.49				
No non-injecting drug use in the past 3 months			0.965			0.799			
No	REF			REF					
Yes	1.02	0.36, 2.59		1.14	0.39, 2.98				
Non-injecting polydrug use in the past 3 months			0.822			0.916			
No	REF			REF					
Yes	0.88	0.25, 2.43		0.94	0.25, 2.76				
OAT status			0.046			0.036			0.081
ever used	REF			REF			REF		
OAT naïve	2.68	1.02, 8.32		2.81	1.06, 8.78		2.38	0.90, 7.40	

^1^ Age, gender, site, arm was adjusted for in the Adjusted Cox Models

^2^ Those significant in the adjusted cox models were selected in this model with conservative cutoff point of 0.1. adjusting for age, gender, site, arm, only those with *p*-value < 0.1 will be left in the final model

^3^ Profile likelihood 95% confidence interval

^4^ Logrank type3 *p*-value

^5^ Firth's method with Breslow for ties was used when there was a zero event count. Elsewhere Efron's method was used for ties

Among partner participants, gender, study site, as well as all drug use variables, were not significant in the univariate analysis. Never receiving OAT was associated with significantly higher mortality in unadjusted and adjusted models (HR = 2.7 [1.0–8.3]; aHR = 2.8 [1.1–8.8]) and allocation to the intervention arm was a strong predictor of survival (HR = 0.2 [0.0–0.8], aHR = 0.2 [0.0–0.8]). In the multivariable model, the study arm remained significant (aHR = 0.2 [0.0–0.9]), while OAT did not (aHR = 2.4 [0.9–7.4]).

## Discussion

In this analysis of the randomized clinical trial conducted at three sites in Indonesia, Vietnam, and Ukraine, we found that PWID, regardless of their HIV status, have significantly higher mortality rates than expected in a population of the same age and gender. The majority of deaths among HIV-infected indexes were related to HIV. The number of deaths due to overdose was low in both participant groups. The intervention, primarily focused on ART initiation and adherence, had a significant effect on mortality for both HIV-infected indexes and their HIV-uninfected partners. Mortality rates differed by country, consistently with the uptake of the intervention [18].

### Mortality rates

The observed crude mortality rates were consistent with the recent systematic review of cohort studies, reporting the pooled CMR of 2.71/100PY among people who inject opioids [2]. In the earlier review that included all PWID the pooled CMR was 2.35/100PY [1], and the mortality rates in HIV-positive were on average 3.15 times greater than in HIV-negative PWID, which is slightly less than 5 times difference in our study. This might be explained by the fact that our study was conducted in low-middle income countries with suboptimal ART coverage, which were underrepresented in the systematic review, and partially by the inclusion criteria that limited our sample to HIV-positive PWID with suppressed (< 1000 cp/ml) viral load at baseline. Another review focusing on non-AIDS mortality among PWID [9], reported the CMR of 2.74/100PY for low and middle income countries, which is similar to our finding.

In our study, the risk of death among PWID living with HIV who were not treated effectively was thirty times higher than expected in a general population adjusted by age and gender, which is twice as high as the pooled SMR in the systematic review. The HIV-uninfected PWID had almost six times higher risk of death compared to the general population, primarily due to acute and chronic health conditions and injuries.

There were marked differences in mortality rates between the sites. Among male indexes, mortality in Indonesia was about twice as high as in Vietnam and Ukraine. In contrast, the male partner mortality was highest in Ukraine, with almost two times difference with Indonesia, and three times with Vietnam. These findings correlate with the differences in uptake of ART and OAT across three sites in our study [18], which could be a plausible explanation. There also were differences related to drug use and injection risk behaviors at baseline [22]. However, given that the site variable remained significant in the multivariable model, adjusting for treatment receipt and drug use, other unaccounted factors, such as access to health care, have likely played a role.

We were not able identify published sources on PWID mortality for Indonesia and Ukraine. We can, however, compare the mortality rates we observed in Vietnam to two intervention trials conducted in Thai Nguyen and a cohort study in Hai Phong provinces. In the first study, conducted in 2005–2007 among male PWID, 23% of whom were HIV infected [23], the CMR was 6.3/100PY and SMR was 13.4. Another trial in male PWID newly diagnosed with HIV in 2009–2011 [15], reported 23% (103/455) of cumulative all-cause mortality at 24 months after enrollment. In the cohort study conducted between 2014 and 2016, mortality was 4.4/100PY and 1.9/100PY, and among HIV-positive and HIV-negative PWID, respectively [24]. In our study, mortality was lower compared to the trials and the HIV-negative cohort, likely due to the effect of our intervention in addition to overall progress in scaling-up ART and OAT in Vietnam [15], but higher compared the HIV-positive cohort in Hai Phong, which may explained by our inclusion criteria that limited the sample to PLWH with unsuppressed viral load.

If we attribute mortality with unconfirmed cause in participants who met immunologic criteria for AIDS (CD4 < 200 cells/μL) to HIV, the HIV-related causes would be responsible for more than half of all deaths among index participants. The majority of confirmed HIV-related deaths were due to tuberculosis. This confirms the fact that PWID continue to face significant barriers related to life-saving HIV and TB treatment engagement and adherence in all three countries. Other health-related causes such as lung or liver diseases were the second most common cause of death in HIV-infected, and the leading cause of death in HIV-negative participants in our study.

Surprisingly, we observed a low rate of death due to overdose, which accounted for almost 1/3 of mortality among opioid injectors in other studies [1]. This finding might be explained by the fact that study subjects were recruited in health care facilities or community centers with good access to overdose prevention and treatment services.

### The effect of the intervention

In our study, we demonstrated a clear and strong effect of the intervention on death outcomes. Among indexes, mortality in the intervention arm compared to the SoC was decreased by more than half. As evident from the multivariable models, the effect of the intervention appears to be mediated by improved immune status, characterized by the CD4 count. Although improved CD4 counts can be plausibly caused only by initiation of ART and resulting viral suppression, these variables did not seem to have an independent effect on mortality in our analysis. Of note, HIV care guidelines since 2015 permitted ART initiation for PWID regardless of CD4 count, which suggests that the observed effect was due to overcoming of other structural and individual-level barriers [17]. The significantly higher OAT uptake in the experimental arm was rather modest overall [18], and also did not demonstrate an independent association with the risk of death. However, increased OAT use could potentiate ART enrollment and retention.

Importantly, the study arm remained significant in the multivariable model controlling for clinical, demographic, and substance use variables. We may hypothesize that the intervention may have facilitated access to other health and social services, and may have promoted healthier living in ways that we did not measure, thereby leading to the reduction in non-HIV related deaths in the experimental arm.

It should be noted, that participants who attained a CD4 cell count of at least 200 cells/μL continued to experience substantially elevated mortality, about 3 times higher than their HIV-negative partners in our study, and up to 18 times higher than expected in their age-matched general population. These mortality rates cannot be directly compared to other studies of patients on ART, because person time calculation in this analysis was starting from study enrollment and not ART initiation. Nevertheless, it is evident in our study, that PWID who had unsuppressed viral load at baseline and initiated ART afterwards had several times higher death rate compared to other PLWH [25], This is consistent with other studies of PWID, and can be explained by later presentation to HIV treatment, high rates of HCV and TB co-infection, and drug-related mortality.

The benefits of the integrated intervention were significant not only for direct recipients but also among their injection partners. One plausible mechanism of this effect could be that by reducing barriers to health care for indexes, our intervention also indirectly motivated and helped their partners to seek care, reduce injection drug use, or increase safer injection practices. Specifically, as reported before [18], our intervention had a moderately positive effect on OAT enrollment among partners. In this analysis, we found about three times the mortality rate in OAT-naïve partners compared to current or past OAT-receiving partners, consistent with a large body of evidence showing the life-saving effect of OAT. In the multivariable model, however, the effect of OAT did not reach the significance threshold, whereas study arm remained strongly associated with reduced mortality, suggesting that there could be other unaccounted factors mediating the effect. These may include the indirect impact on quality of life and psychosocial functioning, which is supported by the fact that there were no deaths related to suicide, violence, or other injuries neither in indexes nor partners of the intervention arm.

### Other predictors of mortality

We assessed the effects of recent injecting and non-injecting substance use on the risk of death. Among indexes, mortality was lower among participants self-reporting hazardous alcohol use or any type of injecting drug use in the past 3 months, and higher among those reporting no injecting or non-injecting drug use. One possible explanation may be that the intensity of drug use among HIV-positive PWID may gradually decline before death due to deteriorating health and decreasing tolerance to substances. Among the HIV-negative partner participants, neither injecting nor non-injecting substance use was associated with mortality.

In contrast to other studies, suggesting higher mortality among opioid users compared to stimulant users [26, 27], we did not observe a significant difference between these sub-groups, regardless of HIV status. This may be explained by the overall low number of deaths caused by opioid overdose.

Importantly, we have also found more than twice the risk of death in index participants with moderate or severe depression. The causal pathway of this association is described by multiple studies showing the negative impact of depression at each stage of HIV treatment continuum: care engagement [28], ART initiation and adherence [29–31], and viral suppression [32, 33], resulting in higher mortality [34–36]. However, some studies reported an independent effect of depression on mortality, controlling for the effects of adherence [37, 38] and clinical variables [35, 39], potentially suggesting other mechanisms. The results from our multivariable model, controlling for clinical variables as well as drug use intensity among PWID, contribute to this evidence. This finding supports the recommendations to include depression screening and treatment in all programs addressing the health of PWID, particularly those living with HIV [40].

Despite the well-documented role of stigma in diminishing access to life-saving treatments for PWID [41–43], we observed an association of anticipated stigma with decreased risk of death in HIV-infected PWID. While other studies suggested that internalized or perceived stigma, as measured by standard scales, can be relatively stable over time and may not necessarily reflect coping and thus changes in access to services [44], our finding may require further investigation.

### Limitations

The participants in our study were recruited using targeted outreach and peer referral; therefore the sample should not be considered representative of the entire PWID population in these countries. Our participants are likely to be more closely linked to HIV prevention and treatment services, also as a result of study participation [45]. Therefore, the mortality estimates and SMRs can be either underestimated (if the recruited sample benefited from the health services) or overestimated (if PWID in poorer health were seeking care). The inclusion criterion of viral load > 1000 cp/ml limited our sample of index participants to those who were either untreated for HIV or treated ineffectively at baseline, also leading to potential overestimation of mortality rates in the index group. Despite these possible biases, our findings are nevertheless close to what has been reported by other studies and pooled LMIC estimates [1, 2].

Our algorithm of assessment of the cause of death relied on investigation by the study teams and was restricted by the lack of contact information and considerations of participant confidentiality. This have led to a substantial proportion of deaths due to unknown causes, and in some cases could also introduce a bias. Verification by three physicians should have minimized the chance of misclassification.

Most of the measures included in this analysis (ART, OAT receipt, drug use, depression) are self-reported, which is prone to recall and social desirability biases. In a sub-study measuring the presence of ART drugs in blood samples [46], we found that bias in self-report of ART use is also bi-directional: some participants reported ART while not taking it, and others did not report it even when laboratory tests detected ARV medications in blood samples. These biases can potentially decrease the accuracy of our risk estimates. On the other hand, the strength of the associations, biological plausibility, and consistency with other studies lend confidence in the validity of our main findings.

## Conclusion

The HPTN 074 randomized controlled trial, conducted in Indonesia, Vietnam, and Ukraine, documented directly measured mortality rates in PWID. The results confirm that both HIV-infected and uninfected PWID remain at a starkly elevated risk of death compared to the general population of the same age. Mortality related to HIV and other causes can be significantly reduced by scaling-up ART and OAT. Access to these life-saving treatments can be effectively improved by flexible integrated interventions, such as the one developed and tested in HPTN 074. Future research may explore strategies that facilitate implementation and scale-up of such interventions.

## Supplementary Information

**Additional file 1.** HPTN 074 Index participants dataset codebook.

**Additional file 2.** HPTN 074 Mortality Data Indexes.

**Additional file 3**. HPTN 074 Mortality Data Partner.

## Data Availability

The datasets supporting the conclusions of this article and respective data dictionaries are included within the additional files.
