# Supplementary material for: Causes and risk factors of death among people who inject drugs in Indonesia, Ukraine and Vietnam: findings from HPTN 074 randomized trial
Source: BMC Infect Dis. 2023 May 11;23:319. doi: 10.1186/s12879-023-08201-3 (PMC10173611; doi:10.1186/s12879-023-08201-3)
Supplement: Supplementary file 1 — Additional file 1. HPTN 074 Index participants dataset codebook. [file 12879_2023_8201_MOESM1_ESM.pdf]

HPTN 074 Index participants dataset codebook

Variable Names	Format	Description
uid		Participant uid
age		Participant age at enrollment
sitedfno	250="Indonesia"<br>251="Ukraine"<br>252="Vietnam"	Study site
gend	1="Female"<br>2="Male"	Participant gender
arm	1= “SOC”<br>2= “Intervention”	Study arm
deceased	1= “Death”<br>0= “Alive”	Mortality status at study close
personyrs		Number of years between enrollment and death (for those who died), and between enrollment and the last visit (for those who were still alive at study close).
dthrsn_i	1="AIDS related"<br>2="Non-AIDS medical"<br>3="Drug related"<br>4="Other cause"	Cause of death
vst_wk		Visit week
auditc	0="Non Hazardous alcohol use"<br>1="Hazardous alcohol use"	Alcohol use in the past 3 months
dg_opd	0="No"<br>1="Yes"	Injected opioids in the past 3 months
dg_stml	0="No"<br>1="Yes"	Injected stimulants in the past 3 months
dg_oth	0="No"<br>1="Yes"	Injected other drugs in the past 3 months
dg_na	0="No"<br>1="Yes"	Injected no drugs in the past 3 months
dg_poly	0="No"<br>1="Yes"	Injected multiple drugs (“Polydrug use”) in the past 3 months
injd		Number of days of injecting drugs use in the past month
dg_opd_nj	0="No"<br>1="Yes"	Non-injected opioid drug use in the past 3 months
dg_stml_nj	0="No"<br>1="Yes"	Non-injected stimulant drug use in the past 3 months
dg_oth_nj	0="No"<br>1="Yes"	Other non-injected drug use in the past 3 months
dg_na_nj	0="No"	No non-injected drug use in the past 3 months

	1="Yes"	
dg_poly_nj	0="No"<br>1="Yes"	Used multiple non-injected drugs (“Polydrug use”) in the past 3 months
artstatt	1="ART naive"<br>2="previously on ART"<br>3="current on ART"	ART status
phq9_2cat	0="no/mild depression"<br>1="moderate/severe"	Depression status according to PHQ9 scale
int_yes	0="No"<br>1="Yes"	Internalized stigma
exp_yes	0="No"<br>1="Yes"	Experienced stigma
ant_yes	0="No"<br>1="Yes"	Anticipated stigma
total_yes	0="No"<br>1="Yes"	Total stigma
matstcatt	1="ever used"<br>2="MAT naive"	MAT status
vl_40	0="non VL suppression"<br>1="VL suppression"	Viral suppression (viral load<40)
cd4_200	0="<200 cells/mm3"<br>1=">=200 cells/mm3"	CD4 count

HPTN 074 Partner participants dataset codebook

Variable Names	Format	Description
uid		Participant uid
age		Participant age at enrollment
sitedfno	250="Indonesia"<br>251="Ukraine"<br>252="Vietnam"	Study site
gend	1="Female"<br>2="Male"	Participant gender
deceased	1= “Death”<br>0= “Alive”	Mortality status at study close
personyrs		Number of years between enrollment and death (for those who died), and between enrollment and the last visit (for those who were still alive at study close).
dthrsn_p	1="Medical"<br>2="Drug-related"<br>3="Other cause"	Cause of death
arm	0= “SOC”<br>1= “Intervention”	Study arm
vst_wk		Visit week
auditc	0="Non Hazardous alcohol use"<br>1="Hazardous alcohol use"	Alcohol use in the past 3 months
dg_opd	0="No"<br>1="Yes"	Injected opioids in the past 3 months
dg_stml	0="No"<br>1="Yes"	Injected stimulants in the past 3 months
dg_oth	0="No"<br>1="Yes"	Injected other drugs in the past 3 months
dg_na	0="No"<br>1="Yes"	Injected no drugs in the past 3 months
dg_poly	0="No"<br>1="Yes"	Injected multiple drugs (“Polydrug use”) in the past 3 months
injd		Number of days of injecting drugs use in the past month
dg_opd_nj	0="No"<br>1="Yes"	Non-injected opioid drug use in the past 3 months
dg_stml_nj	0="No"<br>1="Yes"	Non-injected stimulant drug use in the past 3 months
dg_oth_nj	0="No"<br>1="Yes"	Other non-injected drug use in the past 3 months
dg_na_nj	0="No"<br>1="Yes"	No non-injected drug use in the past 3 months
dg_poly_nj	0="No"<br>1="Yes"	Used multiple non-injected drugs (“Polydrug use”) in the past 3 months

matstcat	1="ever used"<br>2="MAT naive"	MAT status

(HPTN 074) DF/Net 074 (DEM-1) 001

Participant ID: ☐☐☐–☐☐☐☐–☐–☐☐

Site Number *Network Number* *Chk* *Cohort*

Form Completion Date: ☐☐ ☐☐☐ ☐☐

dd *MMM* *yy*

Demographics

Thank you for being a part of our research. People live in different places, with different customs, cultures, sexual practices and beliefs. We hope to include people from different communities in our research. We respect all people, regardless of their differences. Not all questions we ask in our research will apply to you. Because we do not want to make assumptions, we ask the same questions of everyone. We want you to be comfortable in speaking with us. You do not have to answer any question that makes you uncomfortable.

Now I am going to ask you some questions about yourself. The answer to these questions will tell us more about who you are, such as your age and ethnicity (what cultural group you come from). I will also ask you about your sex and gender. Please feel free to ask any questions about things that you don't understand.

**1**	What is your date of birth?	☐☐ ☐☐☐ ☐☐ (*dd* *MMM* *yy*) *OR* *If unknown, record age:* ☐☐ *years*
**2**	What is your sex at birth?	☐ *Male*<br>☐ *Female*
**3**	How do you identify your gender?<br>*Mark all that apply.*	☐ *Male*<br>☐ *Female*<br>☐ *Other, specify:*<br>*Local language:* ________<br>*English:* ________
**4**	Are you currently a student?	☐ *Yes*<br>☐ *No*

This interviewer-administered form is used to collect participants' demographic information.

This form is completed for all participants who were assigned a Participant ID during screening. Submit this CRF with **Screening Outcome** form for those participants who do not enroll. Submit after the enrollment visit only for those participants who enroll.

Item-specific Instructions:

**Item 1**	If any portion of the date of birth is unknown, record age at time of enrollment. If age is unknown, record participant's estimate of their age. Do not complete both answers.
**Item 3**	This item must be self-reported by the participant. Site staff is encouraged to document in chart notes if the participant, during study participation, prefers to be referred to by a specific pronoun or gender.

(HPTN 074) DF/Net 074 (DEM-2) 002

Participant ID:

☐☐☐ – ☐☐☐☐ – ☐ – ☐☐

Site Number *Network Number* *Chk* *Cohort*

Demographics

5 What is the highest level of education you have completed?

- ☐ *No schooling*
- ☐ *Primary school, not complete*
- ☐ *Primary school, complete*
- ☐ *Secondary school, not complete*
- ☐ *Secondary school, complete*
- ☐ *Technical training, not complete*
- ☐ *Technical training, complete*
- ☐ *College or university, not complete*
- ☐ *College or university, complete*

6 What is your relationship status? Are you:

- ☐ *Married*
- ☐ *Living with a sexual partner but not married*
- ☐ *Separated*
- ☐ *Divorced*
- ☐ *Widowed*
- ☐ *Single*
- ☐ *Refuse to answer*

7 Have you been homeless at any time in the last 6 months?

- ☐ *Yes*
- ☐ *No*

This interviewer-administered form is used to collect participants' demographic information.

This form is completed for all participants who were assigned a Participant ID during screening. Submit this CRF with **Screening Outcome** form for those participants who do not enroll. Submit after the enrollment visit only for those participants who enroll.

(HPTN 074) DF/Net 074 (DEM-3) 003

Participant ID: ☐☐☐–☐☐☐☐–☐–☐☐

Site Number *Network Number* *Chk* *Cohort*

Demographics

8 How would you describe your current living situation? *Mark all that apply.*

- ☐ *8a. Live in a house that I own or rent*
- ☐ *8b. Live in parent's home*
- ☐ *8c. Live in other relative's home (e.g, sibling, cousin, aunt and/or uncle)*
- ☐ *8d. Rent a room or space in someone else's house or apartment*
- ☐ *8e. Stay with someone else for free (don't pay regular room and board)*
- ☐ *8f. Live on the street, in a park, alley or abandoned building*
- ☐ *8g. Live in a homeless shelter*
- ☐ *8h. Stay at more than two different places a week*
- ☐ *8i. Live in a drug recovery or any transitional house*
- ☐ *8j. Refuse to answer*
- ☐ *8k. Other place, specify:*

Local language: ______________________

English: ______________________

9 Who lives with you? *Mark all that apply.*

- ☐ *9a. Spouse/partner/girl-boyfriend*
- ☐ *9b. Own children*
- ☐ *9c. Parents*
- ☐ *9d. Other family members*
- ☐ *9e. Friends*
- ☐ *9f. Others (e.g., roommates, tenants)*
- ☐ *9g. Live alone*
- ☐ *9h. Refuse to answer*

This interviewer-administered form is used to collect participants’ demographic information.

This form is completed for all participants who were assigned a Participant ID during screening. Submit this CRF with **Screening Outcome** form for those participants who do not enroll. Submit after the enrollment visit only for those participants who enroll.

(HPTN 074) DF/Net 074 (DEM-4) 004

Participant ID:

☐☐☐–☐☐☐☐–☐–☐☐

Site Number *Network Number* *Chk* *Cohort*

Demographics

10 At any time during the past 3 months, have you been unemployed?

☐ *Yes*

☐ *No*

☐ *Refuse to answer*

11 Which of the following best describes your current employment status? Are you:

☐ *Employed full-time*

☐ *Employed part-time*

☐ *Unemployed but seeking work*

☐ *Unemployed—not seeking work*

☐ *Retired*

☐ *Refuse to answer*

12 In the past month, how many days have you been working for pay? ☐☐ *# days*

This interviewer-administered form is used to collect participants' demographic information.

This form is completed for all participants who were assigned a Participant ID during screening. Submit this CRF with **Screening Outcome** form for those participants who do not enroll. Submit after the enrollment visit only for those participants who enroll.

Item-specific Instructions:

**Items 11-12**	Employment is self-defined by the participant. Document any activity for which the participant is receiving payment for doing work, whether or not it is considered legal employment.

(HPTN 074) DF/Net 074 (DVI) 007

Participant ID: [ ][ ][ ] – [ ][ ][ ][ ] – [ ] – [ ][ ]
Site Number *Network Number* *Chk* *Cohort*

Form Completion Date: [ ][ ] [ ][ ][ ] [ ][ ]
dd *MMM* *yy*

Demographics - Vietnam

1 What does the participant report as his/her ethnicity/nationality? *Mark all that apply.*

- ☐ *1a. Kinh*
- ☐ *1b. Tay*
- ☐ *1c. Nung*
- ☐ *1d. San diu*
- ☐ *1e. H'Mong*
- ☐ *1f. San chi (Trail)*
- ☐ *1g. Hoa*
- ☐ *1h. Dao (Man)*
- ☐ *1i. Cao Lan*
- ☐ *1j. Ngai*
- ☐ *1k. Thai*
- ☐ *1l. Other, specify:* *Local language:* ______________________
  English: ______________________

No instructions necessary.

(HPTN 074) DF/Net 074 (DIN) 009

Participant ID: ☐☐☐ – ☐☐☐☐ – ☐ – ☐☐
Site Number *Network Number* *Chk* *Cohort*

Form Completion Date: ☐☐ ☐☐☐ ☐☐
dd *MMM* *yy*

Demographics - Indonesia

1 What does the participant report as his/her ethnicity/nationality? *Mark all that apply.*

- ☐ *1a. Betawi*
- ☐ *1b. Jawa*
- ☐ *1c. Sunda*
- ☐ *1d. Minang*
- ☐ *1e. Batak*
- ☐ *1f. Bugis*
- ☐ *1g. Ambon*
- ☐ *1h. Tionghoa*
- ☐ *1i. Other, specify:* *Local language:* ______________________
  English: ______________________

No additional instructions needed.

(HPTN 074) DF/Net 074 (DUK) 011

Participant ID: ☐☐☐–☐☐☐☐–☐–☐☐
Site Number *Network Number* *Chk* *Cohort*

Form Completion Date: ☐☐ ☐☐☐ ☐☐
dd *MMM* *yy*

Demographics - Ukraine

1 What does the participant report as his/her ethnicity/nationality? *Mark all that apply.*

- ☐ *1a. Ukrainian*
- ☐ *1b. Russian*
- ☐ *1c. Other, specify:* *Local language:* ______________________

English: ______________________

No additional instructions needed.

(HPTN 074) DF/Net 074 (IPT) 019

Participant ID: ☐☐☐–☐☐☐☐–☐–☐☐ Screening Visit Date: ☐☐ ☐☐☐ ☐☐

Site Number *Network Number* *Chk* *Cohort* *dd* *MMM* *yy*

Index PTID Tracker

1 Was the participant's identity checked (e.g. fingerprint, unique identification card, etc.)? ☐ *Yes* ☐ *No*

If yes, go to item 2.

1a. If no, reason: ____________________

2 Has this participant been seen before for HPTN 074? ☐ *Yes* ☐ *No* *If no, end of form.*

2a. List all prior PTIDs associated with this participant:

	Site Number	Network Number	Chk	Cohort
PTID #1:	☐☐☐	☐☐☐☐	☐	☐☐
PTID #2:	☐☐☐	☐☐☐☐	☐	☐☐
PTID #3:	☐☐☐	☐☐☐☐	☐	☐☐
PTID #4:	☐☐☐	☐☐☐☐	☐	☐☐
PTID #5:	☐☐☐	☐☐☐☐	☐	☐☐

This form is completed for all participants who were assigned a participant ID during screening and is submitted for all screened participants.

Item-specific Instructions:

**Screening Visit Date**	The screening visit date is the date which the participant provided a screening blood sample for HIV testing. A complete date is required.

(HPTN 074) DF/Net 074 (ISO-1) 020

Participant ID: ☐☐☐–☐☐☐☐–☐–☐☐

Site Number *Network Number* *Chk* *Cohort*

Screening Visit Date: ☐☐ ☐☐☐ ☐☐

dd *MMM* *yy*

Index Screening Outcome

1 Did the participant agree to have samples of blood collected at screening stored and used for future testing related to HIV infection?

- ☐ *Yes*
- ☐ *No*

2 How was this participant recruited? *Mark all that apply.*

- ☐ *2a. From another drug injector*
- ☐ *2b. From an HPTN 074 participant*
- ☐ *2c. From a drug user outside of their network*
- ☐ *2d. Social media (e.g., Facebook) or other internet*
- ☐ *2e. Print media*
- ☐ *2f. TV or radio media*
- ☐ *2g. Harm reduction sites/center*
- ☐ *2h. Hot spots*
- ☐ *2i. HIV clinics*
- ☐ *2j. MAT clinics*
- ☐ *2k. Drug sales locations*
- ☐ *2l. Outreach*
- ☐ *2m. Other, specify:* *Local language:* ______________________

  English: ______________________

3 Did the participant enroll in the study?

- ☐ *Yes* → *If yes, end of form. Do not submit page 2.*
- ☐ *No*

The **Index Screening Outcome** CRF documents participant screening and collects information about recruitment and reasons why participants may not enroll in the study.

This form is completed for all participants who were assigned a participant ID during screening and is submitted for all screened participants.

Item-specific Instructions:

**Screening Visit Date**	The screening visit date is the date which the participant provided a screening blood sample for HIV testing. A complete date is required.
**Item 1**	Document the participant's decision regarding screening specimen storage as documented on the screening consent form.

(HPTN 074) DF/Net 074 (ISO-2) 021

Participant ID:

☐☐☐–☐☐☐☐–☐–☐☐

Site Number *Network Number* *Chk* *Cohort*

Index Screening Outcome

4 Why was the participant not enrolled in the study? *Mark all that apply.*

- ☐ *4a. Current participation in an HIV prevention study or previous/current participation in an HIV vaccine study*
- ☐ *4b. Serious and active medical or mental illness*
- ☐ *4c. Previously screened as a potential network partner of another index participant in this study*
- ☐ *4d. Currently or previously a partner of an index participant*
- ☐ *4e. Unwilling to adhere to study procedures*
- ☐ *4f. Withdrew consent/refused to participate*
- ☐ *4g. Unable to recruit network partner within window after screening blood draw*
- ☐ *4h. Viral load < 1,000 copies/mL*
- ☐ *4i. Does not meet the criteria for active injection drug user per protocol*
- ☐ *4j. Not HIV positive*
- ☐ *4k. Does not report sharing needles/syringes or drug solutions at least once in the last month*
- ☐ *4l. Did not complete screening procedures*
- ☐ *4m. Other, specify:* *Local language:* ____________________

  English: ____________________

The **Index Screening Outcome** CRF documents participant screening and collects information about recruitment and reasons why participants may not enroll in the study.

This form is completed for all participants who were assigned a participant ID during screening and is submitted for all screened participants.

Item-specific Instructions:

**Screening Visit Date**	The screening visit date is the date which the participant provided a screening blood sample for HIV testing. A complete date is required.

(HPTN 074) DF/Net 074 (PPT) 024

Participant ID: ☐☐☐–☐☐☐☐–☐–☐☐
Site Number *Network Number* *Chk* *Cohort*

Screening Visit Date: ☐☐ ☐☐☐ ☐☐
dd *MMM* *yy*

Partner PTID Tracker

1 Was the participant's identity checked (e.g. fingerprint, unique identification card, etc.)? ☐ *Yes* ☐ *No*
If yes, go to item 2.

1a. If no, reason: ______________________________

2 Has this participant been seen before for HPTN 074? ☐ *Yes* ☐ *No* *If no, end of form.*

2a. List all prior PTIDs associated with this participant:

	Site Number	*Network Number*	*Chk*	*Cohort*
PTID #1:	☐☐☐	☐☐☐☐	☐	☐☐
PTID #2:	☐☐☐	☐☐☐☐	☐	☐☐
PTID #3:	☐☐☐	☐☐☐☐	☐	☐☐
PTID #4:	☐☐☐	☐☐☐☐	☐	☐☐
PTID #5:	☐☐☐	☐☐☐☐	☐	☐☐

This form is completed for all participants who were assigned a participant ID during screening and is submitted for all screened participants.

Item-specific Instructions:

**Screening Visit Date**	The screening visit date is the date which the participant provided a screening blood sample for HIV testing. A complete date is required.

(HPTN 074) DF/Net 074 (PSO) 025

Participant ID: [ ][ ][ ]-[ ][ ][ ][ ]-[ ]-[ ][ ]
Site Number *Network Number* *Chk* *Cohort*

Screening Visit Date: [ ][ ] [ ][ ][ ] [ ][ ]
dd *MMM* *yy*

Partner Screening Outcome

1 Did the participant agree to have samples of blood collected at screening stored and used for future testing related to HIV infection?

- ☐ *Yes*
- ☐ *No*

2 Did the participant enroll in the study?

- ☐ *Yes* → *If yes, end of form.*
- ☐ *No*

3 Why was the participant not enrolled in the study? *Mark all that apply.*

- ☐ *3a. Any reactive or positive HIV rapid or HIV EIA test*
- ☐ *3b. Current participation in an HIV prevention study or previous/current participation in an HIV vaccine study*
- ☐ *3c. Serious and active medical or mental illness*
- ☐ *3d. Previously screened as a potential network member of another index participant in this study*
- ☐ *3e. Previously named and enrolled as a partner of another index partner*
- ☐ *3f. Unwilling to adhere to study procedures*
- ☐ *3g. Withdrew consent/refused to participate*
- ☐ *3h. Does not meet the criteria for active injection drug user per protocol*
- ☐ *3i. Could not confirm injection relationship with index*
- ☐ *3j. Did not complete screening procedures*
- ☐ *3k. Other, specify:* *Local language:* ______________________

  English: ______________________

The **Partner Screening Outcome** CRF documents participant screening and reasons why participants may not enroll in the study.

This form is completed for all participants who were assigned a participant ID during screening and is submitted for all screened participants.

Item-specific Instructions:

**Screening Visit Date**	The screening visit date is the date which the participant provided a screening blood sample for HIV testing.

(HPTN 074) DF/Net 074 (IAI) 030

Visit Code: ☐☐.☐

Participant ID: ☐☐☐–☐☐☐☐–☐–☐☐

Site Number *Network Number* *Chk* *Cohort*

Visit Date: ☐☐ ☐☐☐ ☐☐ *dd* *MMM* *yy*

Index ART Initiation

1 When did the participant indicate that s/he had initiated ART? ☐☐ ☐☐☐ ☐☐ *dd* *MMM* *yy*

2 How was ART use determined? *Mark all that apply.*

- ☐ *2a. Participant self-report*
- ☐ *2b. Outside clinic report/documentation*
- ☐ *2c. Other, specify:* *Local language:* ____________________ *English:* ____________________

3 Is the participant willing/able to provide documentation?

- ☐ *Yes*
- ☐ *No* → *If no, end of form.*

4 Record ART medications prescribed for the participant:

	ART Medication Code	Date Started (dd MMM yy)
4a.	☐☐☐	☐☐ ☐☐☐ ☐☐
4b.	☐☐☐	☐☐ ☐☐☐ ☐☐
4c.	☐☐☐	☐☐ ☐☐☐ ☐☐
4d.	☐☐☐	☐☐ ☐☐☐ ☐☐
4e.	☐☐☐	☐☐ ☐☐☐ ☐☐

Comments *(Local language)*: ____________________

Comments *(English)*: ____________________

This form documents an Index participant's initiation of ART during the course of the study. This CRF should only be completed once.

Item-specific Instructions:

**Visit Date**	Record the date of the study visit.
**Item 1**	Record the date that the participant indicates that he/she initiated ART.
**Item 3**	Details about the use of ART means a specific listing of medications prescribed. Use source documentation (clinic notes, prescription bottles, etc.). If participant reports ART use by self-report, mark “no.”
**Items 4a-4e**	• **ART Medication Code:** Refer to the HPTN 074 website for the list of ART Medication Codes.<br>• **Date Started:** When possible, record the complete date (day, month, year). If the exact date is unavailable, record the month and year.

(HPTN 074) DF/Net 074 (SUT) 040

Visit Code: ☐☐.☐

Participant ID: ☐☐☐-☐☐☐☐-☐-☐☐

Site Number *Network Number* *Chk* *Cohort*

Visit Date: ☐☐ ☐☐☐ ☐☐
dd *MMM* *yy*

Substance Use Treatment Initiation

1 When did the participant indicate that s/he had initiated substance use treatment? ☐☐ ☐☐☐ ☐☐
dd *MMM* *yy*

2 How was substance use treatment determined? *Mark all that apply.*

☐ *2a. Participant self-report*

☐ *2b. Outside clinic report/documentation*

☐ *2c. Other, specify:* *Local language:* ______________________

English: ______________________

3 Is the participant willing/able to provide details about his/her substance use treatment?

☐ *Yes* ☐ *No* → *If no, end of form.*

4 Record the types of substance use treatment the participant is involved in.

		Yes	*No*	If yes, date of most recent visit: *dd* *MMM* *yy*
4a.	Methadone	☐	☐	☐☐ ☐☐☐ ☐☐
4b.	Buprenorphine	☐	☐	☐☐ ☐☐☐ ☐☐
4c.	Other medication (MAT), specify:<br>*Local Language:* ______________<br>*English:* ______________	☐	☐	☐☐ ☐☐☐ ☐☐
4d.	Detoxification	☐	☐	☐☐ ☐☐☐ ☐☐
4e.	Community treatment/religious, specify:<br>*Local Language:* ______________<br>*English:* ______________	☐	☐	☐☐ ☐☐☐ ☐☐
4f.	Other specify:<br>*Local Language:* ______________<br>*English:* ______________	☐	☐	☐☐ ☐☐☐ ☐☐

Comments *(Local language)*: ______________________

Comments *(English)*: ______________________

This form documents a participant's initiation of substance use treatment during the course of the study. This CRF should be completed only once.

Item-specific Instructions:

**Visit Date**	Record the date of the study visit.
**Item 1**	Record the date that the participant reports that s/he initiated substance use treatment. If the participant has started treatment more than once, indicate the most recent start date.
**Item 3**	Details about the use of substance use treatment means a specific listing of any intervention (MMT, detox, religious, etc.) the participant is engaged in. Use source documentation (clinic notes, prescription bottles, etc.) if possible.
**Items 4a-4f**	**Date Started:** When possible, record the complete date (day, month, year). If the exact date is unavailable, record the month and year.

(HPTN 074) DF/Net 074 (BBA-1) 071

Participant ID: ___ – ___ – ___ – ___
Site Number, Network Number, Chk, Cohort

Assessment Date: ___ ___ ___ (dd MMM yy)

Baseline Behavior Assessment

A broad range of participants are taking part in this study. So some questions may not apply to you. We ask the same questions of all participants.

In this interview, you will be asked about your sexual behaviors and possible alcohol and drug use. Some of the questions may seem very personal. Please remember that all of your answers will be kept private. Your responses are linked only to your study number, not to your name.

We need you to answer these questions honestly. *We do not judge about alcohol or drug use. We make no judgments about how you have sex or the number of times or with whom.*

These questions help us understand the type of behaviors our participants are doing while on study. Your honesty will help us understand these differences in behaviors.

HIV TESTING HISTORY

1 Prior to participating in this study, did you ever have an HIV test? ☐ *Yes* ☐ *No* → *If no, go to item 2.*

1a. How many times have you had an HIV test in your lifetime? ☐☐ *# of times*

1b. When was your most recent HIV test? ___ ___ ___ (*dd MMM yy*)

1c. What was the result of that test? ☐ *Negative* ☐ *Positive* ☐ *Don't know*

INCARCERATION

2 In the last 3 months, how many different times were you in...

2a. Jail/prison? ☐☐ *# of times*

2b. Involuntary detoxification/detention? ☐☐ *# of times*

2c. Other incarceration, specify: ☐☐ *# of times*

Local language: ______________________ *English:* ______________________

This assessment documents information on a participant's behaviors. These questions ask the participant to provide sensitive information. To increase honest and accurate responses it is important to demonstrate that all information will be kept private and confidential.

This is an interviewer-administered form.

Item-specific Instructions:

**Item 1b**	When possible record the complete date (day, month, year). If the exact date is unknown, record month and year.

(HPTN 074) DF/Net 074 (BBA-2) 072

Participant ID: ☐☐☐-☐☐☐☐-☐-☐☐

Site Number *Network Number* *Chk* *Cohort*

Baseline Behavior Assessment

ALCOHOL USE (Audit C) *Answer questions to reflect your behavior over the past 3 months.*

3 How often do you have a drink containing alcohol?

- ☐ *Never* → *If never, go to item 6.*
- ☐ *Monthly or less*
- ☐ *2–4 times a month*
- ☐ *2–3 times a week*
- ☐ *4 or more times a week*

4 How many standard drinks containing alcohol do you have on a typical day?

- ☐ *1 or 2*
- ☐ *3 or 4*
- ☐ *5 or 6*
- ☐ *7 to 9*
- ☐ *10 or more*

5 How often do you have six or more drinks on one occasion?

- ☐ *Never*
- ☐ *Less than monthly*
- ☐ *Monthly*
- ☐ *Weekly*
- ☐ *Daily or almost daily*

NON-INJECTION DRUG USE

6 In the **last 3 months**, did you...

	Yes	*No*
6a. Use marijuana?	☐	☐
6b. Use stimulants (cocaine, methamphetamines)?	☐	☐
6c. Use opiates (heroin, opium)?	☐	☐
6d. Use other drugs *(as indicated by sites)*, specify:	☐	☐

Local language: ______________________

English: ______________________

7 When was the last time you used marijuana, amphetamines, methadone, opiates or benzodiazepines that you did not inject?

☐☐ ☐☐☐ ☐☐ OR *Never* ☐

dd *MMM* *yy*

This assessment documents information on a participant's behaviors. These questions ask the participant to provide sensitive information. To increase honest and accurate responses it is important to demonstrate that all information will be kept private and confidential.

This is an interviewer-administered form.

Item-specific Instructions:

**Item 6**	Record any non-injected use of the drugs indicated (smoke, ingest, sniff, etc.). If appropriate, use local or street name of the drug.
**Item 7**	If the participant has never used these drugs in a non-injected method, mark “never.” When possible record the complete date (day, month, year), if the exact date is unknown record month and year. If appropriate, use local or street name of the drug.

(HPTN 074) DF/Net 074 (BBA-3) 073

Participant ID:

[ ][ ][ ] – [ ][ ][ ][ ] – [ ] – [ ][ ]

Site Number *Network Number* *Chk* *Cohort*

Baseline Behavior Assessment

INJECTION DRUG USE

8 How old were you when you first injected drugs? [ ][ ] *years*

9 What is the longest time you have not injected drugs, voluntarily (e.g. when you were not incarcerated), since you started using injection drugs?

months [ ][ ] *days* [ ][ ] *years* [ ][ ]

10 In the **last 3 months**, which of the following did you inject?

		Yes	*No*
10a.	Amphetamines	☐	☐
10b.	Methamphetamines	☐	☐
10c.	Cocaine	☐	☐
10d.	Heroin	☐	☐
10e.	Opium	☐	☐
10f.	Buprenorphine	☐	☐
10g.	Methadone	☐	☐
10h.	Benzodiazepines	☐	☐
10i.	Ketamine	☐	☐
10j.	Homemade opiods	☐	☐
10k.	Short and long action stimulants	☐	☐
10l.	Dezomorphine	☐	☐
10m.	Other, specify:	☐	☐

Local language: ______________________________

English: ______________________________

11 In **the last month**, on how many days did you inject drugs? [ ][ ] *# of days*

12 In **the last month**, on days that you injected, how many times a day did you usually inject drugs? [ ][ ] *# of times a day*

13 When was the last day you injected (including today)?

[ ][ ] [ ][ ][ ] [ ][ ] OR [ ][ ] *# of months ago*

dd *MMM* *yy*

This assessment documents information on a participant's behaviors. These questions ask the participant to provide sensitive information. To increase honest and accurate responses it is important to demonstrate that all information will be kept private and confidential.

This is an interviewer-administered form.

Item-specific Instructions:

**Item 9**	Record the longest period of time the participant has abstained from injecting drugs since he/she first began injecting drugs.
**Item 10**	If appropriate, use local or street name of the drug.
**Item 13**	When possible, record the complete date (day, month & year). If the exact date is unknown, record month and year.

(HPTN 074) DF/Net 074 (BBA-4) 074

Participant ID: ☐☐☐-☐☐☐☐-☐-☐☐

Site Number *Network Number* *Chk* *Cohort*

Baseline Behavior Assessment

SHARING

Often people share syringes and other equipment because they are in a rush, they are concerned about the police, feeling sick or for a variety of other reasons.

		Yes	No	Don't know
**14**	Have you shared rinse water in **the last** 3 **months**?	☐	☐ *If no, go to item 15.*	☐
	14a. The last time you injected, did you share rinse water?	☐	☐	☐
**15**	Have you shared a cooker/container in **the last** 3 **months**?	☐	☐ *If no, go to item 16.*	☐
	15a. The last time you injected, did you share a cooker/container?	☐	☐	☐
**16**	Have you shared filter cotton in **the last** 3 **months**?	☐	☐ *If no, go to item 17.*	☐
	16a. The last time you injected, did you share filter cotton?	☐	☐	☐

CLEANING

17 How often do you clean your needle before injecting?

☐ *Always* ☐ *More than half the time* ☐ *Less than half the time* ☐ *Never*

		Yes	No	Don't know
**18**	The last time you injected, did you clean the needle before you injected?	☐	☐	☐
**19**	The last time you injected, did you use a new needle?	☐	☐	☐

20 How often do you clean your needle after injecting?

☐ *Always* ☐ *More than half the time* ☐ *Less than half the time* ☐ *Never*

		Yes	No	Don't know
**21**	The last time you injected, did you clean the needle after you injected?	☐	☐	☐

This assessment documents information on a participant's behaviors. These questions ask the participant to provide sensitive information. To increase honest and accurate responses it is important to demonstrate that all information will be kept private and confidential.

This is an interviewer-administered form.

(HPTN 074) DF/Net 074 (BBA-5) 075

Participant ID:

☐☐☐-☐☐☐☐-☐-☐☐

Site Number *Network Number* *Chk* *Cohort*

Baseline Behavior Assessment

PRE-FILLING AND BACKLOADING SYRINGES

**22**	The last time you injected, did you use a pre-filled syringe?	☐ *Yes*	☐ *No*	☐ *Don't know*
**23**	The last time you injected, did you inject drugs that were frontloaded or backloaded into the syringe or needle that you used?	☐ *Yes*	☐ *No*	☐ *Don't know*
**24**	In the last 3 months, how many different people did you use injection drugs with?	☐☐☐	*# of people*	→ *If 000, go to item 30.*
**25**	In the last 3 months, how many times did you even once pass on a needle or syringe to someone else after you used it?	☐☐☐	*# of times*	→ *If 000, go to item 27.*
**26**	In the last 3 months, with how many different people did you pass on a needle or syringe after you had used it?	☐☐☐	*# of people*	
**27**	In the last 3 months, how many times did you ever, even once, use a needle or syringe after someone else used it?	☐☐☐	*# of times*	→ *If 000, go to item 29.*
**28**	In the last 3 months, with how many different people did you use a needle or syringe after someone else used it?	☐☐☐	*# of people*	

29 In the past 3 months, when you shared syringes, how often did you inject last?

☐ *Always* ☐ *Most times* ☐ *Sometimes*

☐ *Rarely* ☐ *Never* ☐ *I have not shared in the past 3 months*

30 In the last 3 months, did you get your new needles/syringes from a…

30a. ☐ *I did not get new needles in the last 3 months* → *Go to item 32 on page 6.*

		Yes	*No*
30b.	Pharmacy or health center?	☐	☐
30c.	Needle seller?	☐	☐
30d.	Needle exchange?	☐	☐
30e.	Other drug injectors?	☐	☐
30f.	Other, specify?	☐	☐

Local language: ______________________ *English:* ______________________

This assessment documents information on a participant's behaviors. These questions ask the participant to provide sensitive information. To increase honest and accurate responses it is important to demonstrate that all information will be kept private and confidential.

This is an interviewer-administered form.

(HPTN 074) DF/Net 074 (BBA-6) 076

Participant ID: [ ][ ][ ]-[ ][ ][ ][ ]-[ ]-[ ][ ]

Site Number *Network Number* *Chk* *Cohort*

Baseline Behavior Assessment

PRE-FILLING AND BACKLOADING SYRINGES

31 In **the last 3 months**, when you obtained new needles, typically, how many did you buy at one time? [ ][ ][ ] *# of needles*

DRUG TREATMENT PROGRAMS *Indicate which types of treatment you have participated in.*

32 Have you ever participated in any type of drug treatment program? ☐ *Yes* ☐ *No* → *If no, go to item 36 on page 7.*

33 Methadone maintenance or any other medication assisted treatment program (MMT/MAT)? ☐ *Yes* ☐ *No* → *If no, go to item 34 on page 7.*

33a. What type of MMT/MAT program have you been on? *Mark all that apply.*

☐ *33a1. Methadone*

☐ *33a2. Buprenorphine*

☐ *33a3. Other, specify:*
(Local language) ______________________
(English) ______________________

33b. How many times have you enrolled in MMT/MAT? [ ][ ][ ] *# of times*

33c. What is the longest period of time you have been in MMT/MAT? [ ][ ] *months* [ ][ ] *days*

33d. Have you participated in MMT/MAT in **the last 3 months**? ☐ *Yes* ☐ *No* → *If no, go to item 34 on page 7.*

33e. What type of MMT/MAT program have you been on in **the last 3 months**? *Mark all that apply.*

☐ *33e1. Methadone*

☐ *33e2. Buprenorphine*

☐ *33e3. Other, specify:*
(Local language) ______________________
(English) ______________________

33f. Are you currently in MMT/MAT? ☐ *Yes* ☐ *No*

→ *If yes, complete the Substance Use Treatment Initiation CRF.*

This assessment documents information on a participant's behaviors. These questions ask the participant to provide sensitive information. To increase honest and accurate responses it is important to demonstrate that all information will be kept private and confidential.

This is an interviewer-administered form.

Item-specific Instructions:

**Item 31**	If the participant has not obtained his/her own needles or cannot remember, enter “000.”
**Item 33c**	Enter both the number of months and days participant was in MMT/MAT. Do not leave any boxes blank.<br>For example, 3 years is 36 months and 00 days. 6 weeks would be recorded as 01 months and 14 days.

(HPTN 074) DF/Net 074 (BBA-7) 077

Participant ID: ☐☐☐–☐☐☐☐–☐–☐☐

Site Number *Network Number* *Chk* *Cohort*

Baseline Behavior Assessment

DRUG TREATMENT PROGRAMS

**34**	A health care provider supported drug detoxification program?	☐ *Yes*	☐ *No*	→ *If no, go to item 35.*
	34a. How many times have you enrolled in detoxification?	☐☐☐ *# of times*		
	34b. What is the longest period of time you have been in detoxification?	☐☐ *months*	☐☐ *days*	
	34c. Have you participated in detoxification in the last 3 months?	☐ *Yes*	☐ *No*	→ *If no, go to item 35.*
	34d. Are you currently in detoxification?	☐ *Yes*	☐ *No*	
**35**	Any type of other drug treatment program (e.g. community treatment/religious)?	☐ *Yes*	☐ *No*	→ *If no, go to item 36.*
	35a. How many times have you enrolled in any other type of drug treatment?	☐☐☐ *# of times*		
	35b. What is the longest period of time you have been in this type of program?	☐☐ *months*	☐☐ *days*	
	35c. Have you participated in this type of program in the last 3 months?	☐ *Yes*	☐ *No*	→ *If no, go to item 36.*
	35d. Are you currently in this type of program?	☐ *Yes*	☐ *No*	

SEXUAL BEHAVIOR QUESTIONS

The next few questions are about sexual behavior. These are very personal questions. Please remember that all the information you give us is confidential. We ask that you answer the questions as honestly as you can.

**36**	In the last 3 months, did you have vaginal or anal sex?	☐ *Yes*	☐ *No*	→ *If no, go to item 42 on page 8.*
**37**	In the last month, how many different female sex partners have you had?	☐☐☐ *# of female sex partners*		
**38**	In the last month, how many different male sex partners have you had?	☐☐☐ *# of male sex partners*		

This assessment documents information on a participant's behaviors. These questions ask the participant to provide sensitive information. To increase honest and accurate responses it is important to demonstrate that all information will be kept private and confidential.

This is an interviewer-administered form.

Item-specific Instructions:

**Item 34**	Health care provider supported drug detoxification program can be voluntary or involuntary, in-patient or out-patient. Do not include alcohol-only detoxification programs.
**Item 34b**	Enter both the number of months and days participant was in detoxification. Do not leave any boxes blank.<br>For example, 3 years is 36 months and 00 days. 6 weeks would be recorded as 01 months and 14 days.
**Item 35**	Other includes any secular, religious-based, or home based programs that are not supported by a healthcare provider.
**Item 35b**	Enter both the number of months and days participant was in MMT/MAT. Do not leave any boxes blank.<br>For example, 3 years is 36 months and 00 days. 6 weeks would be recorded as 01 months and 14 days.

(HPTN 074) DF/Net 074 (BBA-8) 078

Participant ID: ___-____-__-__

Site Number *Network Number* *Chk* *Cohort*

Baseline Behavior Assessment

39 Do you have a main partner such as a husband/wife or boyfriend/girlfriend? ☐ *Yes* ☐ *No* → *If no, go to item 41.*

39a. Is your main partner male or female?

☐ *Male* ☐ *Female* ☐ *Other, specify:* *(Local language)* ____________

(English) ____________

40 In **the last month**, how many times did you have vaginal or anal sex with your main partner? ☐☐☐ *# of times* → *If 000, go to item 41.*

40a. How many of these times did you (or your partner) use a condom? ☐☐☐ *# of times*

41 In **the last month**, how many times did you have vaginal or anal sex with someone other than a main partner? ☐☐☐ *# of times* → *If 000, go to item 42.*

41a. How many of these times did you (or your partner) use a condom? ☐☐☐ *# of times*

Next I am going to ask about your sex trade (paid or paying) partners. By sex trade partners, I mean people that you had oral, vaginal or anal sex with in exchange for money, drugs, food or a place to stay.

42 In **the last month**, how many times did you give sex partners money or drugs in exchange for sex? ☐☐☐ *# of times*

43 In **the last month**, how many times did a sex partner give you money or drugs in exchange for sex? ☐☐☐ *# of times*

This assessment documents information on a participant's behaviors. These questions ask the participant to provide sensitive information. To increase honest and accurate responses it is important to demonstrate that all information will be kept private and confidential.

This is an interviewer-administered form.

Item-specific Instructions:

**Item 39**	"Main partner" is someone you consider your wife/husband/boyfriend/girlfriend, have oral, vaginal or anal sex with, have been in a relationship with for at least 3 weeks. Do not include anyone you have had sex with casually or in exchange for money, drugs, food or a place to stay.

(HPTN 074) DF/Net 074 (IBH-1) 085

Participant ID: ☐☐☐–☐☐☐☐–☐–☐☐ Assessment Date: ☐☐ ☐☐☐ ☐☐

Site Number / Network Number / Chk / Cohort — *dd MMM yy*

Index Baseline HIV Care and ART

The next few questions are about anti-HIV medicines called antiretroviral therapy that you may be taking. These questions are not about drugs you may be taking to treat substance use. We need to know what is really happening, not what you think we "want to hear." Many people find it hard to take their medications all of the time.

1 When did you first find out that you were HIV positive? ☐☐ ☐☐☐ ☐☐ (*dd MMM yy*)

2 With whom have you shared that you are living with HIV? *Mark all that apply.*

- ☐ *2a. Spouse/partner/girl-boyfriend*
- ☐ *2b. Own children*
- ☐ *2c. Parents*
- ☐ *2d. Other family members*
- ☐ *2e. Friends*
- ☐ *2f. Others*
- ☐ *2g. No one*
- ☐ *2h. Refuse to answer*

3 How many of your drug partners (the people you inject drugs with) know that you have HIV?

- ☐ *All of my partners*
- ☐ *More than half of my partners*
- ☐ *Less than half of my partners*
- ☐ *None of my partners*

This is an interviewer-administered form.

(HPTN 074) DF/Net 074 (IBH-2) 086

Participant ID: ☐☐☐-☐☐☐☐-☐-☐☐

Site Number *Network Number* *Chk* *Cohort*

Index Baseline HIV Care and ART

4 Who is the most supportive in helping you with your HIV care and treatment?

- ☐ *Health care providers (such as doctors or nurses)*
- ☐ *Your spouse/live-in partner/long-term partner*
- ☐ *Casual sexual partner (i.e., friends or associates who you just met and had sex with)*
- ☐ *Injecting partners*
- ☐ *Commercial sex workers*
- ☐ *Your mother and father*
- ☐ *Your in-laws*
- ☐ *Other family members*
- ☐ *Priests/religious leaders*
- ☐ *Your neighbors or community members*
- ☐ *Friends*
- ☐ *Your employers*
- ☐ *Other, specify:* *(Local language)* ____________________
  (English) ____________________

5 How often have you talked to your family or support person about your HIV medications?

☐ *More than once per week*	☐ *2–3 times a month*	☐ *One time*
☐ *Once a week*	☐ *Once a month*	☐ *Never*

6 Since you found out you were living with HIV, have you seen an HIV clinician? ☐ *Yes* ☐ *No* → *If no, go to item 8 on page 3.*

6a. If yes, on what date was your most recent visit? ☐☐ (*dd*) ☐☐☐ (*MMM*) ☐☐ (*yy*)

7 Has any HIV clinician ever told you that you should initiate ART treatment for your own health? ☐ *Yes* ☐ *No*

This is an interviewer-administered form.

(HPTN 074) DF/Net 074 (IBH-3) 087

Participant ID: ☐☐☐-☐☐☐☐-☐-☐☐

Site Number *Network Number* *Chk* *Cohort*

Index Baseline HIV Care and ART

**8**	Have you ever visited an HIV clinic or pharmacy with the intention of starting ART?	☐ *Yes*	☐ *No*
**9**	Have you ever been on ART treatment?	☐ *Yes*	☐ *No*
**10**	Are you currently taking ART medications? *If yes, complete Index ART Initiation CRF.*	☐ *Yes*	☐ *No* → *If no, go to Item 16 on page 5.*
**11**	We know that it is very difficult to take your HIV medications all the time. When was the last time you missed taking any doses of your ART medication?<br>☐ *Within the past week* ☐ *3–4 weeks ago* ☐ *Never missed taking medications in past 3 months* → *End of form.*<br>☐ *1–2 weeks ago* ☐ *1–3 months ago*		
**12**	In **the last month**, on about how many days did you miss at least one tablet?	☐☐ *days*	
**13**	Did you miss at least one tablet in **the last four days**?	☐ *Yes*	☐ *No* → *If no, go to Item 15 on page 4.*
	13a. In **the last four days**, on how many days did you miss at least one tablet?	☐☐ *days*	
**14**	Did you miss at least one tablet **yesterday**?	☐ *Yes*	☐ *No*

This is an interviewer-administered form.

(HPTN 074) DF/Net 074 (IBH-4) 088

No data recorded on this page ☐

Participant ID: ☐☐☐–☐☐☐☐–☐–☐☐

Site Number *Network Number* *Chk* *Cohort*

Index Baseline HIV Care and ART

15 The last time you missed at least one tablet, which of these best describes the reason why? *Mark only one. End of form.*

- ☐ *I forgot*
- ☐ *I did not have the medication with me at the time I needed to take it*
- ☐ *I did not want person(s) nearby to see me taking the medication*
- ☐ *I was trying to avoid side effects*
- ☐ *I felt healthy*
- ☐ *I was drunk*
- ☐ *I am not engaging in any behaviors that can transmit HIV to others*
- ☐ *I do not believe the medicines are beneficial*
- ☐ *Not applicable*
- ☐ *Other, specify:* *(Local language)* ____________________

(English) ____________________

This is an interviewer-administered form.

Item-specific Instructions:

**No data recorded on this page**	Mark this box if no data is recorded on this page other than the Participant ID and the Staff Initials/ Date.

(HPTN 074) DF/Net 074 (IBH-5) 089

No data recorded on this page ☐

Participant ID: ☐☐☐-☐☐☐☐-☐-☐☐

Site Number *Network Number* *Chk* *Cohort*

Index Baseline HIV Care and ART

		Agree	*Disagree*	*Not relevant/ Not Applicable*
**16**	Sometimes people have trouble starting HIV treatment (ART) because of personal situations or circumstances. What are the reasons you have not started taking ART?			
	16a. You don't think that you need it.	☐	☐	☐
	16b. You don't have time to go to the clinic.	☐	☐	☐
	16c. You don't have the energy or motivation to go to the clinic.	☐	☐	☐
	16d. You missed an appointment, so it is difficult to go back.	☐	☐	☐
	16e. You have been too sick to go to the clinic.	☐	☐	☐
	16f. Your drug use got in the way of going to the clinic.	☐	☐	☐
	16g. You are worried about the unwanted side effects or complications.	☐	☐	☐
	16h. You don't understand when you are supposed to take each pill.	☐	☐	☐
	16i. You sold or lost your medicine or your medicine was stolen.	☐	☐	☐
	16j. Your drinking (alcohol) got in the way of you going to the clinic.	☐	☐	☐
	16k. You had to wait too long, so you left without being seen.	☐	☐	☐
**17**	Sometimes people find it difficult to start taking ART because of their family or friends. What are the reasons you have not started taking ART?	*Agree*	*Disagree*	*Not relevant/ Not Applicable*
	17a. You don't have enough support from family or friends.	☐	☐	☐
	17b. You don't want others to know you have HIV (e.g., see me take medication).	☐	☐	☐

This is an interviewer-administered form.

Item-specific Instructions:

**No data recorded on this page**	Mark this box if no data is recorded on this page other than the Participant ID and the Staff Initials/ Date.

(HPTN 074) DF/Net 074 (IBH-6) 090

No data recorded on this page ☐

Participant ID: ☐☐☐ – ☐☐☐☐ – ☐ – ☐☐

Site Number | Network Number | Chk | Cohort

Index Baseline HIV Care and ART

		Agree	*Disagree*	*Not relevant/ Not Applicable*
**18**	Many people find it difficult to start taking ART, even if they have previously tried, because of challenges in the health systems/health care process. What were the reasons you have not started taking ART?			
	18a. You do not have the necessary materials (e.g. paperwork, identification, test results) needed by the clinic.	☐	☐	☐
	18b. ART is too expensive.	☐	☐	☐
	18c. ART was not available.	☐	☐	☐
	18d. HIV care provider would not continue your medicine or prescription.	☐	☐	☐
	18e. You do not know how or where to get ART.	☐	☐	☐
	18f. You find transport/travel to the clinic is difficult.	☐	☐	☐
	18g. You have been traveling.	☐	☐	☐
	18h. You have been in jail or incarcerated.	☐	☐	☐
	18i. You are scared of being arrested for your drug use.	☐	☐	☐
	18j. You will need to register with the government as being a drug user.	☐	☐	☐

19 Are there any other reasons you find it difficult to start taking ART?

Local language: ______________________________

English: ______________________________

This is an interviewer-administered form.

Item-specific Instructions:

**No data recorded on this page**	Mark this box if no data is recorded on this page other than the Participant ID and the Staff Initials/ Date.

(HPTN 074) DF/Net 074 (IE) 091

Participant ID: ___ – ____ – _ – __
Site Number, Network Number, Chk, Cohort

Enrollment Date: __ ___ __
dd MMM yy

Index Enrollment

1 Did the participant agree to have their samples of blood collected at enrollment used for future testing related to HIV infection?

- ☐ *Yes*
- ☐ *No*

2 Did the participant agree to have their samples of urine collected at enrollment used for future testing related to drug use?

- ☐ *Yes*
- ☐ *No*

3 To which study arm is the participant assigned?

- ☐ *Standard of care*
- ☐ *Intervention*

This form is completed at Enrollment only.

Item-specific Instructions:

**Items 1 and 2**	Document the index participant’s decision regarding enrollment specimen storage as documented on the enrollment consent form.

(HPTN 074) DF/Net 074 (PE) 095

Participant ID: ☐☐☐–☐☐☐☐–☐–☐☐

Site Number *Network Number* *Chk* *Cohort*

Enrollment Date: ☐☐ ☐☐☐ ☐☐ (*dd* *MMM* *yy*)

Partner Enrollment

1 Did the participant agree to have their samples of blood collected at enrollment used for future testing related to HIV infection?

- ☐ *Yes*
- ☐ *No*

2 Did the participant agree to have their samples of urine collected at enrollment used for future testing related to drug use?

- ☐ *Yes*
- ☐ *No*

3 At which index visit was this network partner enrolled? Index Visit Code: ☐☐.☐

3a. Index enrollment date: Date: ☐☐ ☐☐☐ ☐☐ (*dd* *MMM* *yy*)

This form is completed at Enrollment only.

Item-specific Instructions:

**Items 1 and 2**	Document the participant’s decision regarding enrollment specimen storage as documented on the enrollment consent form.
**Items 3 and 3a**	For network partners that are enrolled after their index partners enrollment visit, indicate the index visit code at which the partner enrolled and enter the index participant's enrollment date.

(HPTN 074) DF/Net 074 (INS) 111

Visit Code: ☐☐.☐

Participant ID: ☐☐☐–☐☐☐☐–☐–☐☐
Site Number *Network Number* *Chk* *Cohort*

Visit Date: ☐☐ ☐☐☐ ☐☐
dd *MMM* *yy*

Index Network Status

1 How many total injection partners are listed on the Social/Sexual Network List? ☐☐☐ *# of partners*

2 How many new injection partners were added to the partner list as of this visit? By new, we mean they had not been in the list prior to this visit. ☐☐☐ *# of new partners*

3 Is a new partner enrolling at this visit? ☐ *Yes* ☐ *No*

3a. Does the index currently have an enrolled partner with whom they are currently (within the last month) sharing needles, water, cookers, etc? ☐ *Yes* ☐ *No*

→ *If both 3 and 3a are no, end of form.*

4 List the enrolled partners with whom the participant has shared needles, water, cookers etc. in the last 3 months (include partners enrolled at this visit).

	Partner's Initials or Nickname:	Participant ID: *Site Number* – *Network Number* – *Chk* – *Cohort*
4a.	______________	☐☐☐–☐☐☐☐–☐–☐☐
4b.	______________	☐☐☐–☐☐☐☐–☐–☐☐
4c.	______________	☐☐☐–☐☐☐☐–☐–☐☐
4d.	______________	☐☐☐–☐☐☐☐–☐–☐☐
4e.	______________	☐☐☐–☐☐☐☐–☐–☐☐
4f.	______________	☐☐☐–☐☐☐☐–☐–☐☐
4g.	______________	☐☐☐–☐☐☐☐–☐–☐☐
4h.	______________	☐☐☐–☐☐☐☐–☐–☐☐
4i.	______________	☐☐☐–☐☐☐☐–☐–☐☐

Comments *(Local Language)*: ______________

Comments *(English)*: ______________

This form is used to document new or returning network partners at follow-up visits.

The index may enroll additional network partners over the course of the study if the relationship and the partner meet protocol eligibility criteria. The index may end a relationship with a network partner, begin a relationship with a network partner or re-establish a relationship with a previous network partner. However, an index participant may only have up to 5 active network partners at any given time.

Item-specific Instructions:

**Item 1**	If no partners are listed on the Social/Sexual Network List (non-CRF), enter 000.
**Item 2**	If no new partners were added, enter 000.
**Item 4**	Returning partners who had previously enrolled in the study maintain his/her original Partner ID.

(HPTN 074) DF/Net 074 (FUV) 120

Visit Code: ☐☐.☐

Participant ID: ☐☐☐–☐☐☐☐–☐–☐☐ (Site Number, Network Number, Chk, Cohort)

Visit Date: ☐☐ ☐☐☐ ☐☐ (dd MMM yy)

Follow-up Visit

1 Is this an interim visit? ☐ *Yes* ☐ *No* → *If no, go to item 2.*

1a. Reason for interim visit. *Mark all that apply.*

- ☐ *1a1. Report a new AE(s)*
- ☐ *1a2. Report a social impact*
- ☐ *1a3. HIV testing*
- ☐ *1a4. New injection partner*
- ☐ *1a5. Repeat laboratory work*
- ☐ *1a6. Other, specify:*
  - *(Local language)* ______________________
  - *(English)* ______________________

1b. Besides this form, what other DataFax forms were completed at this visit? *Mark all that apply.*

- ☐ *1b1. Partner HIV Test Results*
- ☐ *1b2. Index ART Initiation*
- ☐ *1b3. Substance Use Treatment Initiation*
- ☐ *1b4. Urine Drug Screen*
- ☐ *1b5. Specimen Storage*
- ☐ *1b6. CD4+/Viral Load Results*
- ☐ *1b7. Adverse Experience Log (new)*
- ☐ *1b8. Social Impact Log (new)*
- ☐ *1b9. Other, specify:*
  - *(Local language)* ______________________
  - *(English)* ______________________

2 At this visit, how many new Adverse Experiences (AEs) have been reported? ☐☐ → *Complete a separate AE Log page for each AE. If none, enter 00.*

3 At this visit, how many new social impacts have been reported? ☐☐ → *Complete a separate Social Impact Log page for each event. If none, enter 00.*

This form is used to summarize information from each participant follow-up study visit (including interim visits).

Item-specific Instructions:

**Visit Code**	If this form is used for an interim visit, use an interim visit code. If it is used for a scheduled visit, use the visit code required for that visit. If this form is used for an interim visit, all other forms completed for this visit must have the same visit code as the one on this Follow-up Visit form.
**Item 1b**	Mark the newly completed forms (in addition to this form) that are being submitted for the interim visit/contact. If "other, specify" is marked, record the form acronym(s) in the space provided.

(HPTN 074) DF/Net 074 (SIL) 151

Page: ☐☐

Participant ID:

☐☐☐–☐☐☐☐–☐–☐☐

Site Number *Network Number* *Chk* *Cohort*

Social Impact Log

**1**	Concisely describe social impact: ______________________________	
**2**	Onset date:	☐☐ ☐☐☐ ☐☐ *dd* *MMM* *yy*
**3**	Reported at visit:	☐☐.☐
**4**	Social impact code:	☐☐ → *See back for definitions of Social Impact Codes.*

Complete this form when recording the occurrence and resolution of adverse social impacts reported on scheduled Social Impact Assessment Questionnaire forms and those reported spontaneously at any time during the study.

Social impacts are events that the participant thinks are related to participation in this study.

Item-specific Instructions:

<table>
<tr><td>Item 3</td><td>If a participant reports a negative social impact outside of a regularly scheduled visit, complete this Log and the Interim Visit form. Record the same interim visit code on both forms.</td></tr>
<tr><td>Item 4</td><td>Use the following definitions to code the social impact.
<table>
<tr><th colspan="2">Code</th><th>Definition</th></tr>
<tr><td>01</td><td>Personal Relationships</td><td>Had negative experiences with family, friends, significant others, or sex partners.</td></tr>
<tr><td>02</td><td>Travel/Immigration</td><td>Had problems obtaining formal permission to travel to or enter another country, such as being denied a visa, or had a problem with immigration/naturalization.</td></tr>
<tr><td>03</td><td>Employment</td><td>Been turned down for a new job, lost a job, or experienced other problems at work.</td></tr>
<tr><td>04</td><td>Education</td><td>Been turned down by an educational program, told to leave an educational program, or experienced other problems at school.</td></tr>
<tr><td>05</td><td>Medical/Dental</td><td>Been refused medical or dental treatment, or treated negatively by a health care provider.</td></tr>
<tr><td>06</td><td>Health Insurance</td><td>Lost health insurance, had a problem getting new health insurance, or experienced other problems related to health insurance.</td></tr>
<tr><td>07</td><td>Life Insurance</td><td>Lost life insurance, had a problem getting new life insurance, or experienced other problems related to life insurance.</td></tr>
<tr><td>08</td><td>Housing</td><td>Had trouble getting or keeping housing, or had other problems related to housing.</td></tr>
<tr><td>09</td><td>Military/Other Government Agency</td><td>Had a problem with the military or any other government agencies.</td></tr>
<tr><td>10</td><td>Other</td><td>Had other problems not covered in the codes above.</td></tr>
</table>
</td></tr>
</table>

(HPTN 074) DF/Net 074 (SIA-1) 152

Visit Code: ☐☐.☐

Participant ID: ☐☐☐–☐☐☐☐–☐–☐☐

Site Number / Network Number / Chk / Cohort

Contact Date: ☐☐ ☐☐☐ ☐☐ (dd MMM yy)

Social Impact Assessment

1	Because of your participation in this study, did anything negative or bad happen to you that you have not reported to us already?	☐ *Yes* ☐ *No* →	*If no, go to item 4.*
2	Because of your participation in this study, have you...		
	2a. been arrested or had trouble with the police or other legal problems?	☐ *Yes* ☐ *No* →	*If no, go to item 2b.*
	2a1. If yes how many times?	☐☐ *# of times*	
	2b. had trouble getting or keeping housing?	☐ *Yes* ☐ *No* →	*If no, go to item 2c.*
	2b1. If yes how many times?	☐☐ *# of times*	
	2c. had trouble getting or keeping a job or trouble with income or economic support?	☐ *Yes* ☐ *No* →	*If no, go to item 2d.*
	2c1. If yes how many times?	☐☐ *# of times*	
	2d. had trouble getting health care or with health insurance?	☐ *Yes* ☐ *No* →	*If no, go to item 2e.*
	2d1. If yes how many times?	☐☐ *# of times*	
	2e. had personal trouble with friends, family, or acquaintances?	☐ *Yes* ☐ *No* →	*If no, go to item 2f.*
	2e1. If yes how many times?	☐☐ *# of times*	
	2f. had any other type of problem?	☐ *Yes, specify:* ☐ *No* →	*If no, go to item 3.*
	Local language: ____________________	*English:* ____________________	
3	Indicate the total number of impacts:	☐☐ *# of impacts*	*Complete a separate Social Impact Log (SIL) for each impact.*
4	Has your participation in this study had any positive or beneficial impact on your life?	☐ *Yes* ☐ *No* ☐ *Don't know*	*If no, end of form. Do not fax page 2.*

Complete this form when documenting information on any social impact, whether positive or negative, that a participant experiences as a result of being in this HIV prevention trial.

Adverse social impacts associated with study participation must be reported and followed until their resolution using the Social Impact Log. Only report new experiences on this form that have not already been documented on a Social Impact Log. Worsening or continuation of a situation previously reported is not considered a “new” experience.

Item-specific Instructions:

**Items 1-3**	Read all sub-items to the participant and mark a response for each. If the participant refuses any, write “refused” and line through the response box.<br>• If the participant answers “yes” to any responses in item 2, a Social Impact Log page must be completed.<br>• Total the number of social impacts reported and enter the number in the boxes labeled “total number of impacts.” Complete one Social Impact Log page for each impact reported.<br>• If two separate occurrences of the same type of social impact are reported, (e.g., two separate episodes of personal relationship problems), mark the “yes” box for that social impact, and include both episodes in the total. Complete one Social Impact Log page for each episode.
**Item 4**	A “beneficial” impact means anything good or that has improved the participant’s quality of life.

(HPTN 074) DF/Net 074 (SIA-2) 153

Visit Code: ☐☐.☐

Participant ID: ☐☐☐–☐☐☐☐–☐–☐☐

Site Number *Network Number* *Chk* *Cohort*

Social Impact Assessment

5 Because of your participation in this study, have you experienced...

	Yes	No
5a. Employment improvement?	☐	☐
5b. Financial improvement?	☐	☐
5c. Reduction in drug use?	☐	☐
5d. Reduction in cravings/withdrawal?	☐	☐
5e. Gained knowledge?	☐	☐
5f. Life improvement?	☐	☐
5g. Physical health improvement?	☐	☐
5h. Improved relationships?	☐	☐
5i. Reduced stigma?	☐	☐
5j. Improved mental health?	☐	☐
5k. Other, specify:	☐	☐

Local language: ____________________

English: ____________________

Complete this form when documenting information on any social impact, whether positive or negative, that a participant experiences as a result of being in this HIV prevention trial.

Adverse social impacts associated with study participation must be reported and followed until their resolution using the Social Impact Log. Only report new experiences on this form that have not already been documented on a Social Impact Log. Worsening or continuation of a situation previously reported is not considered a “new” experience.

(HPTN 074) DF/Net 074 (STG-1) 155

Visit Code: ☐☐.☐

Participant ID: ☐☐☐–☐☐☐☐–☐–☐☐
Site Number Network Number Chk Cohort

Contact Date: ☐☐ ☐☐☐ ☐☐
dd MMM yy

Social Support and Stigma Questionnaire

In this section, I am going to read you statements about how you may feel about yourself, your injection drug use, and your HIV/AIDS infection. I would like you to tell me if you agree strongly, agree, disagree, or disagree strongly with each statement.

INTERNALIZED STIGMA AND SHAME

		Agree strongly	*Agree*	*Disagree*	*Disagree strongly*
**1**	I think less of myself or I have felt ashamed because:				
	1a. I inject drugs.	☐	☐	☐	☐
	1b. I am living with HIV.	☐	☐	☐	☐

EXPERIENCED STIGMA

		Agree strongly	*Agree*	*Disagree*	*Disagree strongly*
**2**	I have become isolated from my family because:				
	2a. I inject drugs.	☐	☐	☐	☐
	2b. I am living with HIV.	☐	☐	☐	☐

ANTICIPATED STIGMA

		Agree strongly	*Agree*	*Disagree*	*Disagree strongly*
**3**	I fear that if I disclosed my HIV status to my family, they would exclude me from usual family activities.	☐	☐	☐	☐
**4**	I fear that if I disclosed my HIV status to others, I would find it hard to find work.	☐	☐	☐	☐
**5**	I fear that if I go to an MMT or buprenorphine clinic to treat my substance use I will be treated poorly.	☐	☐	☐	☐
**6**	I fear that if I go to an HIV clinic I will be treated poorly because I inject drugs.	☐	☐	☐	☐
**7**	I do not feel comfortable talking to health care providers as I feel they would judge me.	☐	☐	☐	☐

No instructions necessary.

(HPTN 074) DF/Net 074 (STG-2) 156

Visit Code: ☐☐.☐

Participant ID: ☐☐☐-☐☐☐☐-☐-☐☐

Site Number *Network Number* *Chk* *Cohort*

Social Support and Stigma Questionnaire

8 Over the last 2 weeks, how often have you been bothered by any of the following problems?

	Not at all	*Several days*	*More than half of the days*	*Nearly every day*
8a. Little interest or pleasure in doing things	☐	☐	☐	☐
8b. Feeling down, depressed or hopeless	☐	☐	☐	☐
8c. Trouble falling or staying asleep or sleeping too much	☐	☐	☐	☐
8d. Feeling tired or having little energy	☐	☐	☐	☐
8e. Poor appetite or overeating	☐	☐	☐	☐
8f. Feeling bad about yourself—or that you are a failure or have let yourself or your family down	☐	☐	☐	☐
8g. Trouble concentrating on things, such as reading the newspaper or watching television	☐	☐	☐	☐
8h. Moving or speaking so slowly that other people have noticed; or the opposite—being so fidgety or restless that you have to be moving around a lot more than usual	☐	☐	☐	☐
8i. Thoughts that you would be better off dead or of hurting yourself in some way	☐	☐	☐	☐

9 People sometimes look to others for companionship, assistance, or other types of support. Please tell me how often each of the following kinds of support are available to you if you need them.

9a. Someone to have a good time with:

☐ *None of the time* ☐ *A little of the time* ☐ *Some of the time* ☐ *Most of the time*

9b. Someone to get together with for relaxation:

☐ *None of the time* ☐ *A little of the time* ☐ *Some of the time* ☐ *Most of the time*

9c. Someone to do something enjoyable with:

☐ *None of the time* ☐ *A little of the time* ☐ *Some of the time* ☐ *Most of the time*

No instructions necessary.

(HPTN 074) DF/Net 074 (ITB) 158

Visit Code: ☐☐.☐

Participant ID: ☐☐☐–☐☐☐☐–☐–☐☐

Site Number *Network Number* *Chk* *Cohort*

Contact Date: ☐☐ ☐☐☐ ☐☐

dd *MMM* *yy*

Index Treatment Belief Questionnaire

		Agree strongly	*Agree*	*Disagree*	*Disagree strongly*	
**1**	If you have side effects, you should continue to take your HIV treatment.	☐	☐	☐	☐	
**2**	HIV treatment is safe.	☐	☐	☐	☐	
**3**	HIV treatment is effective (by effective we mean, does it work).	☐	☐	☐	☐	
**4**	If you miss your medication one day, what should you do?<br>☐ *Take double dose* ☐ *Take regular dose* ☐ *Take one and half doses*					
**5**	In the past 3 months, have you set personal goals for your HIV treatment?	☐ *Yes*	☐ *No*			
**6**	In the past 3 months, have you asked questions to your medical provider about your HIV?	☐ *Yes*	☐ *No*			
		Agree strongly	*Agree*	*Disagree*	*Disagree strongly*	
**7**	If you have side effects, you should continue to take your substance use treatment.	☐	☐	☐	☐	
**8**	Substitution therapy (site specific, only ask if available) therapy is safe.	☐	☐	☐	☐	*N/A* ☐
**9**	Substance use treatment is effective (by effective we mean, does it work).	☐	☐	☐	☐	
**10**	If you miss your substance use treatment medication one day, what should you do?<br>☐ *Take double dose* ☐ *Take regular dose* ☐ *Take one and half doses*					

Item-specific Instructions:

**Item 8**	If substitution therapy is not available at the site, mark N/A.

(HPTN 074) DF/Net 074 (UDS) 165

Visit Code: ☐☐.☐

Participant ID: ☐☐☐–☐☐☐☐–☐–☐☐

Site Number *Participant Number* *Chk* *Cohort*

Specimen Collection Date: ☐☐ ☐☐☐ ☐☐

dd *MMM* *yy*

Urine Drug Screen

☐ *Not done/Not collected* → *End of form.*

		Yes	*No*	*Not done/ Not collected*
**1**	Was drug detected?			
1a.	Amphetamines	☐	☐	☐
1b.	Methamphetamines	☐	☐	☐
1c.	Methadone	☐	☐	☐
1d.	Opiates/morphine	☐	☐	☐
1e.	Benzodiazepines	☐	☐	☐
1f.	Buprenorphine	☐	☐	☐
1g.	Cannabinoids	☐	☐	☐
1h.	Cocaine metabolite	☐	☐	☐
1i.	Other, specify:	☐	☐	

(Local language) ______________________________

(English) ______________________________

Comments *(Local language)*: ______________________________

Comments *(English)*: ______________________________

Results from tests performed on urine samples (drug screening) are recorded on this form.

Item-specific Instructions:

**Specimen Collection Date**	Record the date that the specimen was collected (not the date results were reported or recorded on the form) for this visit. A complete date is required.
**Not done/Not collected**	If the urine sample was not collected (e.g. the participant refused or forgot), mark "Not done/ Not collected", then end of form.

(HPTN 074) DF/Net 074 (FBA-1) 171

Visit Code: ☐☐.☐

Participant ID: ☐☐☐–☐☐☐☐–☐–☐☐
Site Number / Network Number / Chk / Cohort

Assessment Date: ☐☐ ☐☐☐ ☐☐
dd / MMM / yy

Follow-up Behavior Assessment

A broad range of participants are taking part in this study. So some questions may not apply to you. We ask the same questions of all participants.

In this interview, you will be asked about your sexual behaviors and possible alcohol and drug use. Some of the questions may seem very personal. Please remember that all of your answers will be kept private. Your responses are linked only to your study number, not to your name.

***We need you to answer these questions honestly.** We do not judge about alcohol or drug use. We make no judgments about how you have sex or the number of times or with whom.*

These questions help us understand the type of behaviors our participants are doing while on study. Your honesty will help us understand these differences in behaviors.

FOLLOW-UP DEMOGRAPHICS

1 How would you describe your current living situation? *Mark all that apply.*

- ☐ *1a. Live in a house that I own or rent*
- ☐ *1b. Live in parent's home*
- ☐ *1c. Live in other relative's home (e.g, sibling, cousin, aunt and/or uncle)*
- ☐ *1d. Rent a room or space in someone else's house or apartment*
- ☐ *1e. Stay with someone else for free (don't pay regular room and board)*
- ☐ *1f. Live on the street, in a park, alley or abandoned building*
- ☐ *1g. Live in a homeless shelter*
- ☐ *1h. Stay at more than two different places a week*
- ☐ *1i. Live in a drug recovery or any transitional house*
- ☐ *1j. Other place, specify:*
  - *Local language:* ____________________
  - *English:* ____________________
- ☐ *1k. Refuse to answer*

This assessment documents information on a participant's behaviors. These questions ask the participant to provide sensitive information. To increase honest and accurate responses it is important to demonstrate that all information will be kept private and confidential.

This is an interviewer-administered form.

(HPTN 074) DF/Net 074 (FBA-2) 172

Visit Code: ☐☐.☐

Participant ID: ☐☐☐-☐☐☐☐-☐-☐☐

Site Number *Network Number* *Chk* *Cohort*

Follow-up Behavior Assessment

2 Who lives with you? *Mark all that apply.*

- ☐ *2a. Spouse/partner/girl-boyfriend*
- ☐ *2b. Own children*
- ☐ *2c. Parents*
- ☐ *2d. Other family members*
- ☐ *2e. Friends*
- ☐ *2f. Others (e.g., roommates, tenants)*
- ☐ *2g. Live alone*
- ☐ *2h. Refuse to answer*

3 What is your relationship status? Are you:

- ☐ *Married*
- ☐ *Living with a sexual partner but not married*
- ☐ *Separated*
- ☐ *Divorced*
- ☐ *Widowed*
- ☐ *Single*
- ☐ *Refuse to answer*

4 At any time during the last 3 months, that is from today, have you been unemployed?

- ☐ *Yes*
- ☐ *No*
- ☐ *Refuse to answer*

This assessment documents information on a participant's behaviors. These questions ask the participant to provide sensitive information. To increase honest and accurate responses it is important to demonstrate that all information will be kept private and confidential. Answer these questions to reflect your behavior for the past 3 months.

This is an interviewer-administered form.

(HPTN 074) DF/Net 074 (FBA-3) 173

Visit Code: ☐☐.☐

Participant ID: ☐☐☐-☐☐☐☐-☐-☐☐

Site Number | Network Number | Chk | Cohort

Follow-up Behavior Assessment

5 Which of the following best describes your current employment status? Are you:

- ☐ *Employed full-time*
- ☐ *Employed part-time*
- ☐ *Unemployed but seeking work*
- ☐ *Unemployed—not seeking work*
- ☐ *Retired*
- ☐ *Refuse to answer*

6 In the past month, how many days have you been working for pay? ☐☐ *# days*

7 *(Index: Intervention ONLY)* Do you have a designated support person? ☐ *Yes* ☐ *No* → *If no, go to item 8.*

7a. What is the relationship of the support person to you?

- ☐ *Spouse/partner/girl-boyfriend*
- ☐ *Father/mother*
- ☐ *Sibling*
- ☐ *Other family member*
- ☐ *Friend*
- ☐ *Other, specify:* *Local language:* ______________________

English: ______________________

INCARCERATION

8 In the last 3 months, how many different times were you in...

8a. Jail/prison? ☐☐ *# of times*

8b. Involuntary detoxification/detention? ☐☐ *# of times*

8c. Other incarceration, specify: ☐☐ *# of times*

Local language: ______________________ *English:* ______________________

This assessment documents information on a participant's behaviors. These questions ask the participant to provide sensitive information. To increase honest and accurate responses it is important to demonstrate that all information will be kept private and confidential. Answer these questions to reflect your behavior for the past 3 months.

This is an interviewer-administered form.

(HPTN 074) DF/Net 074 (FBA-4) 174

Visit Code: ☐☐.☐

Participant ID: ☐☐☐-☐☐☐☐-☐-☐☐

Site Number Network Number Chk Cohort

Follow-up Behavior Assessment

ALCOHOL USE (Audit C) *Answer questions to reflect your behavior over the past 3 months.*

9 How often do you have a drink containing alcohol?

- ☐ *Never* → *If never, go to item 12.*
- ☐ *Monthly or less*
- ☐ *2–4 times a month*
- ☐ *2–3 times a week*
- ☐ *4 or more times a week*

10 How many standard drinks containing alcohol do you have on a typical day?

- ☐ *1 or 2*
- ☐ *3 or 4*
- ☐ *5 or 6*
- ☐ *7 to 9*
- ☐ *10 or more*

11 How often do you have six or more drinks on one occasion?

- ☐ *Never*
- ☐ *Less than monthly*
- ☐ *Monthly*
- ☐ *Weekly*
- ☐ *Daily or almost daily*

NON-INJECTION DRUG USE

12 In the last 3 months, did you...

	Yes	*No*
12a. Use marijuana?	☐	☐
12b. Use stimulants (cocaine, methamphetamines)?	☐	☐
12c. Use opiates (heroin, opium)?	☐	☐
12d. Use other drugs *(as indicated by sites)*, specify:	☐	☐

Local language: ______________________

English: ______________________

13 When was the last time you used marijuana, amphetamines, methadone, opiates or benzodiazepines that you did not inject?

☐☐ ☐☐☐ ☐☐ OR *Never* ☐

dd MMM yy

This assessment documents information on a participant's behaviors. These questions ask the participant to provide sensitive information. To increase honest and accurate responses it is important to demonstrate that all information will be kept private and confidential.

This is an interviewer-administered form.

Item-specific Instructions:

**Item 12**	If appropriate, use local or street name of the drug.
**Item 12d**	Record any non-injected use of the drugs indicated (smoke, ingest, sniff, etc.).
**Item 13**	When possible record the complete date (day, month, and year). If the exact date is unknown, record month and year. If the participant has never used these drugs in a non-injected method, mark “never.”

(HPTN 074) DF/Net 074 (FBA-5) 175

Visit Code: ☐☐.☐

Participant ID: ☐☐☐-☐☐☐☐-☐-☐☐

Site Number *Network Number* *Chk* *Cohort*

Follow-up Behavior Assessment

INJECTION DRUG USE

14 In the **last 3 months**, did you use a needle to inject any drugs under your skin or into a vein? ☐ *Yes* ☐ *No* → *If no, go to item 33 on page 8.*

15 In the **last 3 months**, which of the following did you inject?

	Yes	*No*		*Yes*	*No*
15a. Amphetamines	☐	☐	15i. Ketamine	☐	☐
15b. Methamphetamines	☐	☐	15j. Homemade opiods	☐	☐
15c. Cocaine	☐	☐	15k. Short and long action stimulants	☐	☐
15d. Heroin	☐	☐	15l. Dezomorphine	☐	☐
15e. Opium	☐	☐	15m. Other, specify:	☐	☐
15f. Buprenorphine	☐	☐	*Local language:* ______		
15g. Methadone	☐	☐	*English:* ______		
15h. Benzodiazepines	☐	☐			

16 In the **last month**, on how many days did you inject drugs? ☐☐ *# of days*

17 In the **last month**, on days that you injected, how many times a day did you usually inject drugs? ☐☐ *# of times a day*

18 When was the last day you injected (including today)? ☐☐ ☐☐☐ ☐☐ (*dd* *MMM* *yy*) OR ☐☐ *# of months ago*

This assessment documents information on a participant's behaviors. These questions ask the participant to provide sensitive information. To increase honest and accurate responses it is important to demonstrate that all information will be kept private and confidential.

This is an interviewer-administered form.

Item-specific Instructions:

**Item 15m**	If appropriate, use local or street name of the drug. Only record the names of drugs not already marked in 15a–15l.
**Item 18**	When possible record the complete date (day, month, and year). If the exact date is unknown, record month and year.

(HPTN 074) DF/Net 074 (FBA-6) 176

Visit Code: ☐☐.☐

No data recorded on this page ☐

Participant ID: ☐☐☐-☐☐☐☐-☐-☐☐

Site Number *Network Number* *Chk* *Cohort*

Follow-up Behavior Assessment

SHARING

Often people share syringes and other equipment because they are in a rush, they are concerned about the police, feeling sick or for a variety of other reasons.

		Yes	*No*	*Don't know*
**19**	Have you shared rinse water in the last 3 months?	☐	☐	☐
	If no, go to item 20.			
	19a. The last time you injected, did you share rinse water?	☐	☐	☐
**20**	Have you shared a cooker/container in the last 3 months?	☐	☐	☐
	If no, go to item 21.			
	20a. The last time you injected, did you share a cooker/container?	☐	☐	☐
**21**	Have you shared filter cotton in the last 3 months?	☐	☐	☐
	If no, go to item 22.			
	21a. The last time you injected, did you share filter cotton?	☐	☐	☐

CLEANING

22 How often do you clean your needle before injecting?

☐ *Always* ☐ *More than half the time* ☐ *Less than half the time* ☐ *Never*

	Yes	*No*	*Don't know*
22a. The last time you injected, did you clean the needle before you injected?	☐	☐	☐
22b. The last time you injected, did you use a new needle?	☐	☐	☐

22c. How often do you clean your needle after injecting?

☐ *Always* ☐ *More than half the time* ☐ *Less than half the time* ☐ *Never*

	Yes	*No*	*Don't know*
22d. The last time you injected, did you clean the needle after you injected?	☐	☐	☐

This assessment documents information on a participant's behaviors. These questions ask the participant to provide sensitive information. To increase honest and accurate responses it is important to demonstrate that all information will be kept private and confidential.

This is an interviewer-administered form.

Item-specific Instructions:

**No data recorded on this page**	Mark this box if no data is recorded on this page other than the Participant ID, Visit Code, and the Staff Initials/Date.

(HPTN 074) DF/Net 074 (FBA-7) 177

Visit Code: ☐☐.☐

No data recorded on this page ☐

Participant ID: ☐☐☐–☐☐☐☐–☐–☐☐

Site Number *Network Number* *Chk* *Cohort*

Follow-up Behavior Assessment

PRE-FILLING AND BACKLOADING SYRINGES

**23**	The last time you injected, did you use a pre-filled syringe?	☐ *Yes* ☐ *No* ☐ *Don't know*	
**24**	The last time you injected, did you inject drugs that were frontloaded or backloaded into the syringe or needle that you used?	☐ *Yes* ☐ *No* ☐ *Don't know*	
**25**	In the last 3 months, how many different people did you use injection drugs with?	☐☐☐ *# of people*	→ *If 000, go to item 31.*
**26**	In the last 3 months, how many times did you even once pass on a needle or syringe to someone else after you used it?	☐☐☐ *# of times*	→ *If 000, go to item 28.*
**27**	In the last 3 months, with how many different people did you pass on a needle or syringe after you had used it?	☐☐☐ *# of people*	
**28**	In the last 3 months, how many times did you ever, even once, use a needle or syringe after someone else used it?	☐☐☐ *# of times*	→ *If 000, go to item 30.*
**29**	In the last 3 months, with how many different people did you use a needle or syringe after someone else used it?	☐☐☐ *# of people*	

30 In the past 3 months, when you shared syringes, how often did you inject last?

☐ *Always* ☐ *Most times* ☐ *Sometimes*

☐ *Rarely* ☐ *Never* ☐ *I have not shared in the past 3 months*

31 In the last 3 months, did you get your new needles/syringes from a…

31a. ☐ *I did not get new needles in the last 3 months* → *Go to item 33 on page 8.*

		Yes	*No*
31b.	Pharmacy or health center?	☐	☐
31c.	Needle seller?	☐	☐
31d.	Needle exchange?	☐	☐
31e.	Other drug injectors?	☐	☐
31f.	Other, specify?	☐	☐

Local language: ______________________ *English:* ______________________

32 In the last 3 months, when you obtained new needles, typically, how many did you buy at one time? ☐☐☐ *# of needles*

This assessment documents information on a participant's behaviors. These questions ask the participant to provide sensitive information. To increase honest and accurate responses it is important to demonstrate that all information will be kept private and confidential.

This is an interviewer-administered form.

Item-specific Instructions:

**No data recorded on this page**	Mark this box if no data is recorded on this page other than the Participant ID, Visit Code, and the Staff Initials/Date.
**Item 32**	If the participant has not obtained his/her own needles or cannot remember, enter “000.”

(HPTN 074) DF/Net 074 (FBA-8) 178

Visit Code: ☐☐.☐

Participant ID: ☐☐☐-☐☐☐☐-☐-☐☐

Site Number *Network Number* *Chk* *Cohort*

Follow-up Behavior Assessment

SEXUAL BEHAVIOR QUESTIONS

The next few questions are about sexual behavior. These are very personal questions. Please remember that all the information you give us is confidential. We ask that you answer the questions as honestly as you can.

**33**	In **the last month**, did you have vaginal or anal sex?	☐ *Yes* ☐ *No* → *If no, go to item 39.*
**34**	In **the last month**, how many different **female** sex partners have you had?	☐☐☐ *# of female sex partners*
**35**	In **the last month**, how many different **male** sex partners have you had?	☐☐☐ *# of male sex partners*
**36**	Do you have a main partner such as a husband/wife or boyfriend/girlfriend?<br>36a. Is your main partner male or female?<br>☐ *Male* ☐ *Female* ☐ *Other, specify:* *(Local language)* ____________<br>*(English)* ____________	☐ *Yes* ☐ *No* → *If no, go to item 38.*
**37**	In **the last month**, how many times did you have vaginal or anal sex with your main partner?<br>37a. How many of these times did you (or your partner) use a condom?	☐☐☐ *# of times* → *If 000, go to item 38.*<br>☐☐☐ *# of times*
**38**	In **the last month**, how many times did you have vaginal or anal sex with someone other than a main partner?<br>38a. How many of these times did you (or your partner) use a condom?	☐☐☐ *# of times* → *If 000, go to item 39.*<br>☐☐☐ *# of times*

Next I am going to ask about your sex trade (paid or paying) partners. By sex trade partners, I mean people that you had oral, vaginal or anal sex with in exchange for money, drugs, food or a place to stay.

**39**	In **the last month**, how many times did you give sex partners money or drugs in exchange for sex?	☐☐☐ *# of times*
**40**	In **the last month**, how many times did a sex partner give you money or drugs in exchange for sex?	☐☐☐ *# of times*

This assessment documents information on a participant's behaviors. These questions ask the participant to provide sensitive information. To increase honest and accurate responses it is important to demonstrate that all information will be kept private and confidential.

This is an interviewer-administered form.

Item-specific Instructions:

**Item 36**	"Main partner" is someone you consider your wife/husband/boyfriend/girlfriend, have oral, vaginal or anal sex with, have been in a relationship with for at least 3 weeks. Do not include anyone you have had sex with casually or in exchange for money, drugs, food or a place to stay.

(HPTN 074) DF/Net 074 (PHT) 181

Visit Code: ☐☐.☐

Participant ID: ☐☐☐-☐☐☐☐-☐-☐☐

Site Number *Network Number* *Chk* *Cohort*

Partner HIV Test Results

HIV TEST RESULTS

**1**	Specimen #1 Collection Date: ☐☐ ☐☐☐ ☐☐ (*dd* *MMM* *yy*) OR *Not done/ Not collected* ☐ → *If not done/not collected at a visit where HIV testing is required by the protocol, provide reason in Comments.*<br>1a. Were any of the HIV test results positive or Reactive? ☐ *Yes* ☐ *No*	
**2**	Specimen #2 Collection Date: ☐☐ ☐☐☐ ☐☐ (*dd* *MMM* *yy*) OR *Not done/ Not collected* ☐ → *If not done/not collected at a visit where HIV testing is required by the protocol, provide reason in Comments.*<br>2a. Were any of the HIV test results positive or Reactive? ☐ *Yes* ☐ *No*	
**3**	Final HIV Status: ☐ *Negative* ☐ *Positive* ☐ *Indeterminate*	

Comments *(Local language)*: ______________________________

____

Comments *(English)*: ______________________________

____

The **Partner HIV Test Results** CRF documents the results of HIV testing performed at the site at scheduled and interim visits. Record test results on this form as they become available from the local lab. Fax this form to DataFax when the final test results are available and recorded.

Item-specific Instructions:

**Specimen Collection Date**	Record the date that the specimen(s) was collected (NOT the date results were reported or recorded on the form) for this visit.

(HPTN 074) DF/Net 074 (IFH-1) 185

Visit Code: ☐☐.☐

Participant ID: ☐☐☐-☐☐☐☐-☐-☐☐

Site Number / Network Number / Chk / Cohort

Assessment Date: ☐☐ ☐☐☐ ☐☐ (dd MMM yy)

Index Follow-up HIV Care and ART

The next few questions are about anti-HIV medicines called antiretroviral therapy that you may be taking. These questions are not about drugs you may be taking to treat substance use. We need to know what is really happening, not what you think we "want to hear." Many people find it hard to take their medications all of the time.

1 Since your last visit, have you seen an HIV clinician? ☐ *Yes* ☐ *No* → ***If no, go to item 2.***

1a. If yes, on what date? ☐☐ ☐☐☐ ☐☐ (*dd MMM yy*)

1b. How many total clinical visits have you had since your last study visit? ☐☐ *# of clinical visits*

2 Since your last study visit, with whom have you shared that you are living with HIV? *Mark all that apply.*

- ☐ *2a. Spouse/Partner/Girl-Boyfriend*
- ☐ *2b. Own children*
- ☐ *2c. Parents*
- ☐ *2d. Other family members*
- ☐ *2e. Friends*
- ☐ *2f. Others*
- ☐ *2g. No one*
- ☐ *2h. Refuse to answer*

3 How many of your drug partners (the people you inject drugs with) know that you have HIV?

- ☐ *All of my partners*
- ☐ *More than half of my partners*
- ☐ *Less than half of my partners*
- ☐ *None of my partners*

This is an interviewer-administered form.

Item-specific Instructions:

**Item 1a**	When possible record the complete date (day, month, year). If the exact date is unknown, record month and year.

(HPTN 074) DF/Net 074 (IFH-2) 186

Visit Code: ☐☐.☐

Participant ID: ☐☐☐-☐☐☐☐-☐-☐☐

Site Number | Network Number | Chk | Cohort

Index Follow-up HIV Care and ART

4 Who is the most supportive in helping you with your HIV care and treatment?

- ☐ *Health care providers (such as doctors or nurses)*
- ☐ *Your spouse/live-in partner/long-term partner*
- ☐ *Injecting partners*
- ☐ *Commercial sex workers*
- ☐ *Your mother or father*
- ☐ *Your in-laws*
- ☐ *Other family members*
- ☐ *Priests/religious leaders*
- ☐ *Your neighbors or community members*
- ☐ *Friends*
- ☐ *Your employers*
- ☐ *Other, specify:*

Local language: ____________ *English:* ____________

5 How often have you talked to your family or support person about your HIV medications?

- ☐ *More than once per week*
- ☐ *Once a week*
- ☐ *2–3 times a month*
- ☐ *Once a month*
- ☐ *One time*
- ☐ *Never*

6 Since your last visit, has any HIV clinician told you that you should initiate ART treatment for your own health?

- ☐ *Yes*
- ☐ *No*
- ☐ *Not applicable*

7 Since your last visit, have you visited an HIV clinic or pharmacy with the intention of starting ART? ☐ *Yes* ☐ *No*

This is an interviewer-administered form.

Item-specific Instructions:

**Item 6**	If the participant has not seen an HIV clinician since their last visit, mark “Not applicable.”

(HPTN 074) DF/Net 074 (IFH-3) 187

Visit Code: ☐☐.☐

Participant ID: ☐☐☐-☐☐☐☐-☐-☐☐

Site Number *Network Number* *Chk* *Cohort*

Index Follow-up HIV Care and ART

8 Are you currently taking HIV medications (ART)? ☐ *Yes* ☐ *No* → ***If no, go to item 16 on page 4.***

8a. Since what date have you been on HIV medications (ART) treatment? ☐☐ (*dd*) ☐☐☐ (*MMM*) ☐☐ (*yy*) ***OR*** ☐ *Since last study visit*

9 Since your last study visit, with whom have you shared that you are taking ART? *Mark all that apply.*

- ☐ *9a. Spouse/Partner/Girl-Boyfriend*
- ☐ *9b. Own children*
- ☐ *9c. Parents*
- ☐ *9d. Other family members*
- ☐ *9e. Friends*
- ☐ *9f. Others*
- ☐ *9g. No one*
- ☐ *9h. Refuse to answer*
- ☐ *9i. I've not taken ART since my last study visit*

10 Since your last study visit, has your doctor changed your HIV medications (ART) drugs? ☐ *Yes* ☐ *No* → ***If no, go to item 11.***

10a. Were your HIV medications (ART) drugs changed due to side effects or some other reason? ☐ *side effects* ☐ *something else*

11 We know that it is very difficult to take your HIV medications all the time. When was the last time you missed taking any doses of your ART medication?

- ☐ *Within the past week*
- ☐ *1–2 weeks ago*
- ☐ *3–4 weeks ago*
- ☐ *1–3 months ago*
- ☐ *Never missed taking medications in past 3 months* → ***End of form.***

12 In the **last month**, on about how many days did you miss at least one tablet? ☐☐ *days*

13 Did you miss at least one tablet in **the last four days**? ☐ *Yes* ☐ *No* → ***If no, go to item 15 on page 4.***

13a. In **the last four days**, on how many days did you miss at least one tablet? ☐☐ *days*

14 Did you miss at least one tablet **yesterday**? ☐ *Yes* ☐ *No*

This is an interviewer-administered form.

(HPTN 074) DF/Net 074 (IFH-4) 188

Visit Code: ☐☐.☐

No data recorded on this page ☐

Participant ID: ☐☐☐–☐☐☐☐–☐–☐☐

Site Number | Network Number | Chk | Cohort

Index Follow-up HIV Care and ART

15 The last time you missed at least one tablet, which of these best describes the reason why? *Mark only one.* ***End of form.***

- ☐ *I forgot*
- ☐ *I did not have the medication with me at the time I needed to take it*
- ☐ *I did not want person(s) nearby to see me taking the medication*
- ☐ *I was trying to avoid side effects*
- ☐ *I felt healthy*
- ☐ *I was drunk*
- ☐ *I am not engaging in any behaviors that can transmit HIV to others*
- ☐ *I do not believe the medicines are beneficial*
- ☐ *not applicable—no missed tablets*
- ☐ *Other, specify:*

Local language: ________________ *English:* ________________

16 If you were on ART since your last visit, have you stopped? By stopped we mean that you have not taken any of your medication for the last 2 weeks. ☐ *Yes* ☐ *No* → ***If no, go to item 18.***

17 What date did you stop taking ART? ☐☐ ☐☐☐ ☐☐ (*dd* *MMM* *yy*)

This is an interviewer-administered form.

Item-specific Instructions:

**No data recorded on this page**	Mark this box if no data is recorded on this page other than the Participant ID, Visit Code, and the Staff Initials/Date.

(HPTN 074) DF/Net 074 (IFH-5) 189

Visit Code: ☐☐.☐

No data recorded on this page ☐

Participant ID: ☐☐☐-☐☐☐☐-☐-☐☐

Site Number *Network Number* *Chk* *Cohort*

Index Follow-up HIV Care and ART

		Agree	Disagree	Not relevant/ Not Applicable
**18**	Sometimes people have trouble taking HIV medications (ART) because of personal situations or circumstances. What are the reasons you have stopped taking ART or have not yet started to take ART?			
	18a. You don't think that you need it.	☐	☐	☐
	18b. You don't have time to go to the clinic.	☐	☐	☐
	18c. You don't have the energy or motivation to go to the clinic.	☐	☐	☐
	18d. You missed an appointment, so it is difficult to go back.	☐	☐	☐
	18e. You have been too sick to go to the clinic.	☐	☐	☐
	18f. Your drug use got in the way of going to the clinic.	☐	☐	☐
	18g. You had unwanted side effects or complications.	☐	☐	☐
	18h. You don't understand when you are supposed to take each pill.	☐	☐	☐
	18i. You sold or lost your medicine or your medicine was stolen.	☐	☐	☐
	18j. Your drinking (alcohol) got in the way of going back to the clinic.	☐	☐	☐
	18k. You had to wait too long, so you left without being seen.	☐	☐	☐
**19**	Sometimes people find it difficult to take HIV medications (ART) because of their family or friends. What are the reasons you have stopped taking ART or have not yet started to take ART?	*Agree*	*Disagree*	*Not relevant/ Not Applicable*
	19a. You don't have enough support from family or friends.	☐	☐	☐
	19b. You don't want others to know you have HIV (e.g., see me take medication).	☐	☐	☐

This is an interviewer-administered form.

Item-specific Instructions:

**No data recorded on this page**	Mark this box if no data is recorded on this page other than the Participant ID, Visit Code, and the Staff Initials/Date.

(HPTN 074) DF/Net 074 (IFH-6) 190

Visit Code: __ __ . __

No data recorded on this page ☐

Participant ID: ___ - ____ - _ - __

Site Number | Network Number | Chk | Cohort

Index Follow-up HIV Care and ART

		Agree	Disagree	Not relevant/ Not applicable
**20**	Many people find it difficult to take HIV medications (ART), even if they have previously taken ART, because of challenges in the health systems/health care process. What were the reasons you have stopped taking ART or have not yet started to take ART?			
	20a. You do not have the necessary materials (e.g. paperwork, identification, test results) needed by the clinic.	☐	☐	☐
	20b. ART is too expensive.	☐	☐	☐
	20c. ART was not available.	☐	☐	☐
	20d. HIV care provider would not continue your medicine or prescription.	☐	☐	☐
	20e. You do not know how or where to get ART.	☐	☐	☐
	20f. You find transport/travel to the clinic is difficult.	☐	☐	☐
	20g. You have been traveling.	☐	☐	☐
	20h. You have been in jail or incarcerated.	☐	☐	☐
	20i. You are scared of being arrested for your drug use.	☐	☐	☐
	20j. You will need to register with the government as being a drug user.	☐	☐	☐

21 Are there any other reasons you find it difficult to continue taking ART or why you have not yet started to take ART?

Local language: ______________________________

English: ______________________________

This is an interviewer-administered form.

Item-specific Instructions:

**No data recorded on this page**	Mark this box if no data is recorded on this page other than the Participant ID, Visit Code, and the Staff Initials/Date.

(HPTN 074) DF/Net 074 (FSU-1) 201

Visit Code: __ __ . __

Participant ID: ___ – ____ – _ – __

Site Number *Network Number* *Chk* *Cohort*

Assessment Date: __ ___ __

dd *MMM* *yy*

Follow-up Substance Use Treatment

1 Since your last visit, have you tried to start MAT (methadone or buprenorphine) to treat your substance use?

☐ *Yes* ☐ *No* → *If no, go to item 2.*

1a. When was your last visit? __ ___ __ (*dd* *MMM* *yy*)

2 Are you currently taking methadone or buprenorphine?

☐ *Yes* ☐ *No* → *If no, go to item 3.*

→ *If yes, and this is the participant's first time in MAT during the study, complete the Substance Use Treatment Initiation CRF.*

2a. How long have you currently been on MAT (methadone or buprenorphine)? __ *months*

3 If you were on substance use treatment since your last visit, have you stopped taking MAT or buprenorphine? By stopped, we mean that you have not been taking it for at least 2 weeks.

☐ *Yes* ☐ *No* → *If no, end of form.*

3a. What date did you stop taking MMT or buprenorphine? __ ___ __ (*dd* *MMM* *yy*)

This CRF monitors the participant's status in substance use treatment throughout the study.

This is an interviewer-administered form.

(HPTN 074) DF/Net 074 (FSU-2) 202

Visit Code: ☐☐.☐

No data recorded on this page ☐

Participant ID: ☐☐☐–☐☐☐☐–☐–☐☐

Site Number *Network Number* *Chk* *Cohort*

Follow-up Substance Use Treatment

4 Sometimes people find it difficult to continue MMT or buprenorphine because of personal situations or circumstances. What are the reasons you have stopped MMT or buprenorphine?

	Agree	*Disagree*	*Not relevant/ Not applicable*
4a. You want to keep injecting.	☐	☐	☐
4b. You want to handle your drug problem or stop injecting on your own.	☐	☐	☐
4c. You had side effects or withdrawal symptoms.	☐	☐	☐
4d. Your injecting got in the way of going to the clinic.	☐	☐	☐
4e. Your drug use got in the way of going to the clinic.	☐	☐	☐
4f. You have been too sick to go to the clinic.	☐	☐	☐
4g. You missed an appointment, so it is difficult to go back.	☐	☐	☐
4h. You can handle your drug problem yourself.	☐	☐	☐
4i. Your drinking (alcohol) got in the way of going to the clinic.	☐	☐	☐

5 Sometimes people find it difficult to continue MMT or buprenorphine because of their family or friends. What are the reasons you have stopped MMT or buprenorphine?

	Agree	*Disagree*	*Not relevant/ Not applicable*
5a. You don't have enough support from family or friends.	☐	☐	☐
5b. You don't want to stop seeing the friends you inject with.	☐	☐	☐
5c. Your family objects to drug treatment.	☐	☐	☐
5d. You don't feel you have enough support from friends and family.	☐	☐	☐

This CRF monitors the participant's status in substance use treatment throughout the study.

This is an interviewer-administered form.

Item-specific Instructions:

**No data recorded on this page**	Mark this box if no data is recorded on this page other than the Participant ID, Visit Code, and the Staff Initials/Date.

(HPTN 074) DF/Net 074 (FSU-3) 203

Visit Code: ☐☐.☐

No data recorded on this page ☐

Participant ID: ☐☐☐-☐☐☐☐-☐-☐☐

Site Number *Network Number* *Chk* *Cohort*

Follow-up Substance Use Treatment

6 Many people find it difficult to continue MMT or buprenorphine, even if they have previously tried, because of challenges in the health systems/health care process. What were the reasons you have not continued MMT or buprenorphine?

		Agree	*Disagree*	*Not relevant/ Not applicable*
6a.	The waiting list is too long.	☐	☐	☐
6b.	You don't have the necessary materials (ID card, paperwork).	☐	☐	☐
6c.	You find getting on drug treatment is too much of a hassle.	☐	☐	☐
6d.	You do not know how or where to get drug treatment.	☐	☐	☐
6e.	You find transport/travel to the clinic is difficult.	☐	☐	☐
6f.	You have been traveling.	☐	☐	☐
6g.	You have been in jail or incarcerated.	☐	☐	☐
6h.	You are scared of being arrested for your drug use.	☐	☐	☐
6i.	You are scared of losing your job.	☐	☐	☐
6j.	You were asked to leave MAT.	☐	☐	☐

7 Are there any other reasons you find it difficult to continue MAT or buprenorphine?

Local language: ______________________________

English: ______________________________

This CRF monitors the participant's status in substance use treatment throughout the study.

This is an interviewer-administered form.

Item-specific Instructions:

**No data recorded on this page**	Mark this box if no data is recorded on this page other than the Participant ID, Visit Code, and the Staff Initials/Date.

(HPTN 074) DF/Net 074 (SNE-1) 210

Encounter #: ☐☐☐

Note: Number encounters sequentially (001, 002, 003) for each participant.

Participant ID: ☐☐☐–☐☐☐☐–☐–☐☐
Site Number Network Number Chk Cohort

Assessment Date: ☐☐ ☐☐☐ ☐☐
dd MMM yy

Systems Navigator Encounter

1 Staff ID: ______________

2 Activity type: ☐ *Initial* ☐ *Weekly/Monthly* ☐ *Interim*

3 Contact initiated/requested by:

- ☐ *Systems navigator*
- ☐ *Psychosocial counselor*
- ☐ *Agency/clinic*
- ☐ *Participant's designated support person*
- ☐ *Participant*
- ☐ *Other, specify:* *Local language:* ______________ *English:* ______________

4 Type of encounter:

- ☐ *In person (not at home)*
- ☐ *Home contact*
- ☐ *Telephone*
- ☐ *Social media*
- ☐ *E-mail/Internet*
- ☐ *Text/SMS*
- ☐ *Other, specify:* *Local language:* ______________ *English:* ______________

5 Is the participant currently taking antiretroviral therapy (ART)? ☐ *Yes* ☐ *No*

6 Is the participant currently receiving methadone or buprenorphine? ☐ *Yes* ☐ *No*

7 Does the participant have a designated support person? ☐ *Yes* ☐ *No* → *If no, go to item 8 on page 2.*

7a. Did the support person participate in this contact activity? ☐ *Yes* ☐ *No*

7b. What is the relationship of the support person to the participant?

- ☐ *Spouse/partner/girl- boyfriend*
- ☐ *Father/Mother*
- ☐ *Sibling*
- ☐ *Other family member*
- ☐ *Friend*
- ☐ *Other, specify:* *Local language:* ______________ *English:* ______________

Use this CRF in conjunction with the Intervention Manual to document participant encounters with the system navigator. Complete this CRF any time the participant has contact with a systems navigator.

Item-specific Instructions:

**Encounter Number**	Number encounters sequentially throughout the study starting with 001. Do not repeat encounter numbers. Do not re-number any encounters after faxing, unless instructed by the SDMC.
**Staff ID**	Identify the staff member conducting the encounter. Do not use full names, document only the staff member's initials (last, first). If more than one staff members share initials utilize a middle initial as well.
**Activity Type**	Indicate whether this communication is the initial, weekly or monthly contact as outlined in the protocol. If the contact is not a scheduled encounter, or due to an ongoing crisis period, indicate "interim."

(HPTN 074) DF/Net 074 (SNE-2) 211

Encounter #: ☐☐☐

Note: Number encounters sequentially (001, 002, 003) for each participant.

Participant ID: ☐☐☐ – ☐☐☐☐ – ☐ – ☐☐

Site Number *Network Number* *Chk* *Cohort*

Systems Navigator Encounter

8 Total number of minutes of activity:

- ☐ *0–5 minutes*
- ☐ *6–10 minutes*
- ☐ *11–15 minutes*
- ☐ *16–30 minutes*
- ☐ *31–60 minutes*
- ☐ *60+ minutes*

9 Primary focus of contact:

- ☐ *Substance use treatment*
- ☐ *HIV care*
- ☐ *Needle and syringe exchange programs*
- ☐ *ART initiation/Management*
- ☐ *Assistance with primary designated support person*
- ☐ *Social network management*
- ☐ *Additional counseling*
- ☐ *Legal/Judicial*
- ☐ *Sexual health services*
- ☐ *Social services*
- ☐ *Priests/religious leaders*
- ☐ *Your neighbors or community members*
- ☐ *Clinical/medical management*
- ☐ *Other, specify:*

Local language: ______________________________

English: ______________________________

10 Additional areas addressed: *Mark all that apply.*

- ☐ *10a. Substance use treatment*
- ☐ *10b. HIV care*
- ☐ *10c. Needle and syringe exchange programs*
- ☐ *10d. ART initiation/Management*
- ☐ *10e. Assistance with primary designated support person*
- ☐ *10f. Social network management*
- ☐ *10g. Additional counseling*
- ☐ *10h. Legal/Judicial*
- ☐ *10i. Sexual health services*
- ☐ *10j. Social services*
- ☐ *10k. Priests/religious leaders*
- ☐ *10l. Your neighbors or community members*
- ☐ *10m. Clinical/medical management*
- ☐ *10n. Other, specify:*

Local language: ______________________________

English: ______________________________

Use this CRF in conjunction with the Intervention Manual to document participant encounters with the system navigator. Complete this CRF any time the participant has contact with a systems navigator.

Item-specific Instructions:

**Encounter Number**	Number encounters sequentially throughout the study starting with 001. Do not repeat encounter numbers. Do not re-number any encounters after faxing, unless instructed by the SDMC.

(HPTN 074) DF/Net 074 (SNE-3) 212

Encounter #: ☐☐☐

Note: Number encounters sequentially (001, 002, 003) for each participant.

Participant ID: ☐☐☐-☐☐☐☐-☐-☐☐

Site Number *Network Number* *Chk* *Cohort*

Systems Navigator Encounter

11 Assessment of client status:

- ☐ *Client has no immediate complicating psychosocial issues*
- ☐ *Client has complicating psychosocial issues that post minimal risk to ART or substance use adherence*
- ☐ *Client has complicating psychosocial issues that pose moderate risk to ART or substance use adherence*
- ☐ *Client has complicating psychosocial issues that pose severe risk to ART or substance use adherence*

12 Was a referral made at this encounter? ☐ *Yes* ☐ *No* → *If no, go to item 13.*

12a. What was the referral made for? ***Mark all that apply.***

- ☐ *HIV care (ART)*
- ☐ *Needle/syringe exchange program*
- ☐ *Initial substance use treatment*
- ☐ *Mental health counseling*
- ☐ *Reentry into substance use treatment*

13 Was a social impact reported at this encounter? ☐ *Yes* ☐ *No* → *If yes, complete Social Impact Log for each impact. If this is an interim visit, also complete a Follow-up Visit form.*

14 Was a Serious Adverse Experience reported at this encounter? ☐ *Yes* ☐ *No* → *If yes, complete AE Log for each reported AE. If this is an interim visit, also complete a Follow-up Visit form.*

15 Did the participant report initiating any antiretroviral medications for treatment at this encounter? ☐ *Yes* ☐ *No* → *If yes, complete Index ART Initiation CRF. If this is an interim visit, also complete a Follow-up Visit form.*

16 Did the participant report initiating substance use treatment (MAT) at this encounter? ☐ *Yes* ☐ *No* → *If yes, complete Substance Use Treatment Initiation CRF. If this is an interim visit, also complete a Follow-up Visit form.*

Use this CRF in conjunction with the Intervention Manual to document participant encounters with the system navigator. Complete this CRF any time the participant has contact with a systems navigator.

Item-specific Instructions:

**Encounter Number**	Number encounters sequentially throughout the study starting with 001. Do not repeat encounter numbers. Do not re-number any encounters after faxing, unless instructed by the SDMC.

(HPTN 074) DF/Net 074 (PSY-1) 225

Encounter #: ☐☐☐

Note: Number encounters sequentially (001, 002, 003) for each participant.

Participant ID: ☐☐☐–☐☐☐☐–☐–☐☐ (Site Number, Network Number, Chk, Cohort)

Assessment Date: ☐☐ ☐☐☐ ☐☐ (dd MMM yy)

Psychosocial Encounter

1 Counselor ID: ____________________

2 Contact type:

- ☐ *Study site visit*
- ☐ *Telephone contact*
- ☐ *Off-site contact, specify:* *Local language:* ____________________ *English:* ____________________

3 Activity type:

- ☐ *Introductory session*
- ☐ *First session*
- ☐ *Second session*
- ☐ *Booster session*
- ☐ *Index and supporter session*

4 Does the participant have a designated support person? ☐ *Yes* ☐ *No* → *If no, go to item 5 on page 2.*

4a. Did the support person participate in this contact activity? ☐ *Yes* ☐ *No*

4b. What is the relationship of the support person to the participant?

- ☐ *Spouse/partner/girl- boyfriend*
- ☐ *Father/Mother*
- ☐ *Sibling*
- ☐ *Other family member*
- ☐ *Friend*
- ☐ Other, specify: *Local language:* ____________________ *English:* ____________________

Use this CRF in conjunction with the Intervention Manual to document participant encounters with the psychosocial counselor. Complete this CRF any time the participant has contact with a psychosocial counselor.

Item-specific Instructions:

**Counselor ID**	Identify the staff member conducting the encounter. Do not use full names, document only the staff member's initials.

(HPTN 074) DF/Net 074 (PSY-2) 226

Encounter #: ☐☐☐

Note: Number encounters sequentially (001, 002, 003) for each participant.

Participant ID: ☐☐☐–☐☐☐☐–☐–☐☐

Site Number *Network Number* *Chk* *Cohort*

Psychosocial Encounter

5 Indicate focus of session. *Mark all that apply.*

- ☐ *5a. Dealing with HIV infection*
- ☐ *5b. HIV disclosure*
- ☐ *5c. Risk reduction*
- ☐ *5d. ART adherence and/or development of medication adherence plan*
- ☐ *5e. Engagement into care*
- ☐ *5f. Adherence communication skills*
- ☐ *5g. Sexual risk communication skills*
- ☐ *5h. Injection risk communication skills*
- ☐ *5i. Relationships/Social support*
- ☐ *5j. HIV literacy*
- ☐ *5k. Other, specify:* *Local language:* ______________________

English: ______________________

Use this CRF in conjunction with the Intervention Manual to document participant encounters with the psychosocial counselor. Complete this CRF any time the participant has contact with a psychosocial counselor.

(HPTN 074) DF/Net 074 (PSY-3) 227

Encounter #: ☐☐☐

Note: Number encounters sequentially (001, 002, 003) for each participant.

Participant ID: ☐☐☐ – ☐☐☐☐ – ☐ – ☐☐

Site Number *Network Number* *Chk* *Cohort*

Psychosocial Encounter

6 Barriers to ART addressed. *Mark all that apply.*

- ☐ *6a. Need for ART*
- ☐ *6b. Hassle of taking medication*
- ☐ *6c. Time to go to the clinic*
- ☐ *6d. Challenges getting to the clinic*
- ☐ *6e. Challenges/Issues in the clinic*
- ☐ *6f. Previously missed appointments*
- ☐ *6g. Too sick*
- ☐ *6h. Alcohol use interference*
- ☐ *6i. Drug use interference*
- ☐ *6j. Don't have food/water to take medicine*
- ☐ *6k. Don't understand when/how to take medicine*
- ☐ *6l. Side effects/complications*
- ☐ *6m. Family or friend support*
- ☐ *6n. Disclosure issues, including fear of inadvertent disclosure, strategies for intentional disclosure*
- ☐ *6o. Navigating structural issues–referral to systems navigator*
- ☐ *6p. Other, specify:* *Local language:* ____________________ *English:* ____________________
- ☐ *6q. No barriers addressed*

Use this CRF in conjunction with the Intervention Manual to document participant encounters with the psychosocial counselor. Complete this CRF any time the participant has contact with a psychosocial counselor.

(HPTN 074) DF/Net 074 (PSY-4) 228

Encounter #: ☐☐☐

Note: Number encounters sequentially (001, 002, 003) for each participant.

Participant ID: ☐☐☐-☐☐☐☐-☐-☐☐

Site Number *Network Number* *Chk* *Cohort*

Psychosocial Encounter

7 *Barriers to MMT/buprenorphine addressed. Mark all that apply.*

- ☐ *7a. Need for MMT/buprenorphine*
- ☐ *7b. Hassle of taking medication*
- ☐ *7c. Time to go to the clinic*
- ☐ *7d. Going to the clinic is a hassle*
- ☐ *7e. Previously missed appointments*
- ☐ *7f. Too sick*
- ☐ *7g. Alcohol use interference*
- ☐ *7h. Drug use interference*
- ☐ *7i. Don't have food/water to take medicine*
- ☐ *7j. Don't understand when/how to take medicine*
- ☐ *7k. Side effects/complications*
- ☐ *7l. Family or friend support*
- ☐ *7m. Navigating structural issues–referral to systems navigator*
- ☐ *7n. Other, specify:* *Local language:* ____________________ *English:* ____________________
- ☐ *7o. No barriers addressed*

8 Total number of minutes of activity:

0–5 minutes ☐	*6–10 minutes* ☐	*11–15 minutes* ☐
16–30 minutes ☐	*31–60 minutes* ☐	*60+ minutes* ☐

9 As a result of this counseling, were systems navigator services provided? ☐ *Yes* ☐ *No*

Use this CRF in conjunction with the Intervention Manual to document participant encounters with the psychosocial counselor. Complete this CRF any time the participant has contact with a psychosocial counselor.

(HPTN 074) DF/Net 074 (SS) 230

Visit Code: ☐☐.☐

Participant ID: ☐☐☐-☐☐☐☐-☐-☐☐

Site Number *Network Number* *Chk* *Cohort*

Specimen Storage

1 PLASMA

Specimen Collection Date: ☐☐ ☐☐☐ ☐☐ (*dd* *MMM* *yy*)

☐ *Stored* ☐ *Not stored* ☐ *Not collected* ☐ *Not required*

1a. Reason not stored or not collected: *Local language:* ______ *English:* ______

2 FROZEN URINE

Specimen Collection Date: ☐☐ ☐☐☐ ☐☐ (*dd* *MMM* *yy*)

☐ *Stored* ☐ *Not stored* ☐ *Not collected* ☐ *Not required*

2a. Reason not stored or not collected: *Local language:* ______ *English:* ______

3 DRIED URINE CARTRIDGE

Specimen Collection Date: ☐☐ ☐☐☐ ☐☐ (*dd* *MMM* *yy*)

☐ *Stored* ☐ *Not stored* ☐ *Not collected* ☐ *Not required*

3a. Reason not stored or not collected: *Local language:* ______ *English:* ______

4 DRIED URINE FILTER PAPER

Specimen Collection Date: ☐☐ ☐☐☐ ☐☐ (*dd* *MMM* *yy*)

☐ *Stored* ☐ *Not stored* ☐ *Not collected* ☐ *Not required*

4a. Reason not stored or not collected: *Local language:* ______ *English:* ______

Comments (*Local language):* ______

Comments (*English):* ______

This form is used to document the storage of specimens that will be tested at a lab other than the site local laboratory.

Item-specific Instructions:

**Items 1-4**	• Mark “not stored” if the specimen was collected as required at this visit but was not stored.<br>• Mark “not collected” if the specimen is required to be collected and stored at this visit but was not collected.<br>• Mark “not required” if a specimen is not required and was not collected and stored at this visit.

(HPTN 074) DF/Net 074 (ACL) 232

Page: ☐☐

Participant ID: ☐☐☐–☐☐☐☐–☐–☐☐
Site Number | Network Number | Chk | Cohort

Date of Contact: ☐☐ ☐☐☐ ☐☐
dd | MMM | yy

Agency Contact

1 Contact initiated by:

- ☐ *Systems navigator*
- ☐ *Counselor*
- ☐ *Agency/clinic*
- ☐ *Other, specify: (Local Language)* ____________ *(English)* ____________

2 Type of facility contacted:

- ☐ *HIV clinic*
- ☐ *Substance use treatment facility*
- ☐ *Needle and syringe exchange program*
- ☐ *Mental health counselor/facility*
- ☐ *Sexual health services*
- ☐ *Social services*
- ☐ *Clinical/medical services*
- ☐ *Legal/judicial*
- ☐ *Other, specify: (Local Language)* ____________ *(English)* ____________

3 Type of contact:

- ☐ *In person*
- ☐ *Telephone*
- ☐ *Email/internet*
- ☐ *Text message/SMS*
- ☐ *Other, specify: (Local Language)* ____________ *(English)* ____________

This form documents each contact between HPTN 074 study staff and any referral sites/agencies that are made on the behalf of an index participant. One **Agency Contact** CRF should be submitted for each contact that is made.

(HPTN 074) DF/Net 074 (CDF) 330

Visit Code: ☐☐.☐

Participant ID: ☐☐☐-☐☐☐☐-☐-☐☐

Site Number *Network Number* *Chk* *Cohort*

Initial Specimen Collection Date: ☐☐ ☐☐☐ ☐☐ (*dd* *MMM* *yy*)

CD4+ Count and Viral Load

1 CD4 + Count: ☐ *Not done/ Not collected*

Alternate Collection Date: ☐☐ ☐☐☐ ☐☐ (*dd* *MMM* *yy*)

1a. Absolute CD4+: ☐ *Unable to analyze* ☐☐☐☐ *cells/mm3*

2 HIV-1 RNA PCR: ☐ *Not done/ Not collected*

Alternate Collection Date: ☐☐ ☐☐☐ ☐☐ (*dd* *MMM* *yy*)

2a. HIV RNA PCR (plasma): **<** ☐ **=** ☐ **>** ☐ *viral copies/mL* ☐☐☐☐☐☐☐☐ *OR* ☐ *Undetectable*

2b. RNA PCR kit code: ☐☐☐☐ *kit code*

Comments (*Local language):* ______________________________

Comments (*English):* ______________________________

This form is to document a participant's CD4+ and Viral Load results as collected per protocol.

Item-specific Instructions:

<table>
<tr><td>Initial Specimen Collection Date</td><td>Record the date that the first specimen(s) was collected (not the date results were reported or recorded on the form) for this visit. A complete date is required.</td></tr>
<tr><td>Not done/Not collected</td><td>Mark this box in the event that a specimen was not collected, or if the specimen was collected, but a result is not available due to specimen loss or damage.</td></tr>
<tr><td>Alternate Collection Date</td><td>This date is to be completed ONLY if the specimen was collected after the Initial Specimen Collection Date for this same visit. A specimen collected for the same visit but on a different day should be recorded on the same form only when obtained within the same visit window. A complete date is required.</td></tr>
<tr><td>Results Reporting</td><td>If a specimen was collected but results are not available because the specimen was lost or damaged, line through the results box(es), provide initials and date, and write an explanation in Comments.
If the site lab does not produce test results in the units used on this form, the results must be converted before the laboratory CRF is faxed to SCHARP. Refer to Study Specific Procedures (SSP) for conversion instructions.
It may be necessary to round the result reported by the lab up or down to the level of precision allowed on the CRF.
If the site lab does not produce test results in the units used on this form, first perform the conversion, then round the converted result if necessary.</td></tr>
<tr><td>Item 2a</td><td>Mark the less than (<), equal to (=), or greater than (>) box as appropriate and record the value. For example, if the value is 3100 copies, mark the equal to (=) box and record “0003100” as the value. If the value reported is < 400 copies, mark the less than (<) box and record “0000400” as the value.</td></tr>
<tr><td>Item 2b</td><td>Record the four-digit RNA PCR kit code from the table below.<table><tr><th>RNA PCR Kit</th><th>Code</th></tr><tr><td>Abbott m2000 Real-time</td><td>0406</td></tr><tr><td>Roche Cobas AmpliPrep/Cobas Tagman Ver. 1.0</td><td>0108</td></tr><tr><td>Roche Cobas AmpliPrep/Cobas Tagman Ver. 2.0</td><td>0110</td></tr></table></td></tr>
</table>

(HPTN 074) DF/Net 074 (AE) 460

Page: [ ][ ]

Participant ID: [ ][ ][ ]–[ ][ ][ ][ ]–[ ]–[ ][ ]
Site Number *Network Number* *Chk* *Cohort*

Date Reported to Site: [ ][ ] [ ][ ][ ] [ ][ ]
dd *MMM* *yy*

Adverse Experience Log

**1**	Adverse Experience (AE): *Record diagnosis if available. Include anatomical location, if applicable.*	
**2**	Onset date:	[ ][ ] [ ][ ][ ] [ ][ ] *dd MMM yy*
**3**	Severity Grade:	☐ *Grade 1—mild* ☐ *Grade 3—severe* ☐ *Grade 5—death* ☐ *Grade 2—moderate* ☐ *Grade 4—potentially life-threatening*
**4**	Relationship to study product:	☐ *Related* ☐ *Not related* *If not related, record rationale or alternative etiology in Comments.*
**5**	Study product administration:	☐ *No change* ☐ *Held* ☐ *Permanently discontinued* ☐ *N/A*
**6**	Status or Outcome of AE:	☐ *Continuing* ☐ *Resolved* ☐ *Death* ☐ *Severity/frequency increased (Report as new AE)* ☐ *Continuing at end of study participation* → 6a. Status/Outcome Date *(Leave blank if Status/ Outcome is "continuing".)* [ ][ ] [ ][ ][ ] [ ][ ] *dd MMM yy*
**7**	Treatment: *Mark "none" or all that apply.*	☐ *None* ☐ *Medication(s)* ☐ *Procedure/surgery Comment below.* ☐ *New/prolonged hospitalization Comment below.* ☐ *Other, specify: Comment below.* ____________
**8**	Is this an SAE according to ICH guidelines?	☒ *Yes* ☐ *No*
**9**	Has or will this AE be reported as an EAE? (if a SUSAR)?	☐ *Yes* ☐ *No*
**10**	At which visit was this AE first reported? *Visit code required (regular or interim).*	[ ][ ] . [ ] *visit code*

Comments *(Local language):* ______________________________

Comments *(English):* ______________________________

To document Grade 3 or higher Adverse Experience (AE) and only SAEs.

If a cluster of symptoms reported on separate AE Log pages is later attributed to a single diagnosis, change the earliest reported symptom to the final diagnosis. In addition, mark the AE Log pages for the other symptoms with the words “Delete due to diagnosis on AE page #” (specify page number of diagnosis AE).

Item-specific Instructions:

**Page**	Number pages sequentially throughout the study, starting with 001. Do not repeat page numbers. Do not renumber any AE Log pages after faxing, unless instructed by SCHARP.
**Item 1**	Whenever possible, provide a diagnosis instead of listing a cluster of symptoms. If no diagnosis is identified, each symptom must be recorded on a separate page of the AE Log. If an abnormal lab value is reported, record the lab assay with the direction (i.e., increased or decreased) of the abnormality.
**Item 2**	At minimum, month and year are required. Record one of the following, as appropriate: the date on which the participant reports first experiencing the AE; if the AE is discovered during the study visit exam, record the date of the study visit exam; if the AE is an abnormal lab result, record the date on which the specimen was collected.
**Item 3**	Report only Grade 3 or higher AEs and only SAEs. To grade the severity of an AE, consult the *Division of AIDS (DAIDS) Table for Grading the Severity of Adult and Pediatric Adverse Experiences.*
**Item 4**	Study agent is defined as the participant's current or last known ART and/or opiate substitution therapy. Mark the assessment of the relationship between the AE and the study agent. Mark “related” if there is a reasonable possibility that the AE may be related to the study agent. Mark “not related” if there is not a reasonable possibility that the AE is related to the study agent. If “not related,” record an alternative etiology, diagnosis, or explanation in Comments. For more information, refer to the *Manual for Expedited Reporting of Adverse Events to DAIDS, Version 2*.
**Item 5**	• **No change:** Mark if the participant is expected to continue to use study product and the AE does NOT result in a study product hold or permanent discontinuation.<br>• **Held**: Mark if the AE results in a study product hold. If multiple AEs are reported at the same visit, mark “held” for the AE(s) that contributed to the product hold.<br>• **Permanently discontinued:** Mark if the AE results in permanent discontinuation of study product. If multiple AEs are reported at the same visit, only mark “permanently discontinued” for the AE that contributed to the permanent discontinuation.<br>• **N/A (not applicable):** Mark if the AE occurred after the participant had completed all administration of the study product, or the study product is held or permanently discontinued for a different AE or other reason, or the AE is grade 5-death.
**Item 6**	• **Continuing:** AE is continuing at the time it is reported.<br>• **Resolved:** Condition is no longer present, or returned to the pre-enrollment severity/frequency. If a participant is taking a medication to control an AE that arose during study participation, it is not considered resolved.<br>• **Death:** Mark only if the severity of this AE is grade 5. Any other AEs continuing at the time of death should be changed to “continuing at end of study participation.”<br>• **Severity/frequency increased:** If an AE increases in severity or frequency after it has been reported on the AE Log, line through the “continuing” box previously marked and mark “severity/frequency increased.” Record the date of increase in the “Status/Outcome Date.” Report the increase in severity or frequency as a new AE. For this new AE, the “Onset Date” will be the date that the severity or frequency increased. Update EAE form if applicable. Note that decreases in severity should not be recorded as new AEs.<br>• **Continuing at end of study participation:** Mark this box whenever an AE is continuing at the time of participant study termination.
**Item 6a**	At minimum, month and year are required. Record one of the following, as appropriate: the date on which the participant no longer experienced the AE; or the date of the study visit or specimen collection at which the change in status/outcome is first noted.
**Item 7**	Indicate all treatments administered for this AE, including treatment provided by a health care professional and participant self-treatment. Do not indicate treatments that were clinically indicated or prescribed but not administered.
**Items 8 and 9**	For questions about ICH guidelines and EAE reporting, refer to the *Manual for Expedited Reporting of Adverse Events to DAIDS, Version 2.*

(HPTN 074) DF/Net 074 (MV) 463

Visit Code: [ ][ ] . [ ]

Participant ID: [ ][ ][ ] – [ ][ ][ ][ ] – [ ] – [ ][ ]
Site Number *Network Number* *Chk* *Cohort*

Form Completion Date: [ ][ ] [ ][ ][ ] [ ][ ]
dd *MMM* *yy*

Missed Visit

1 Target Visit Date: [ ][ ] [ ][ ][ ] [ ][ ]
dd *MMM* *yy*

2 Reason visit was missed. *Mark only one.*

- ☐ 2a. Unable to contact participant
- ☐ 2b. Unable to schedule appointment(s) within window
- ☐ 2c. Participant refused visit
- ☐ 2d. Participant incarcerated
- ☐ 2e. Participant admitted to a health care facility
- ☐ 2f. Participant withdrew from the study → *Complete Termination form.*
- ☐ 2g. Participant deceased → *Complete Termination form. Complete Adverse Event Log.*
- ☐ 2h. Participant travelled outside the province for work
- ☐ 2i. Other, specify: *(Local language)* ____________________
  (English) ____________________

Comments (*Local language*): ____________________

____

Comments (*English*): ____________________

____

Complete this form whenever an enrolled participant misses a required visit according to the visit window outlined in the protocol or Study-specific Procedures (SSP).

If the QC Report indicates that a visit is overdue, confirm that the visit was missed before completing a Missed Visit form. Fax this form when it is determined that a visit has been missed and cannot be completed within the visit window. Record the Visit Code of the visit that was missed. Record the date that the form was completed. This will not necessarily be the date of the missed visit.

Item-specific Instructions:

**Item 1**	Record the target date of the visit. A complete date is required.
**Item 2**	Record the reason the participant missed the visit.

(HPTN 074) DF/Net 074 (TM) 490

Participant ID: ☐☐☐ – ☐☐☐☐ – ☐ – ☐☐

Site Number *Network Number* *Chk* *Cohort*

Termination

1 Termination Date: ☐☐ ☐☐☐ ☐☐ (*dd* *MMM* *yy*)

Date the site determined that the participant was no longer in the study.

2 Reason for termination. *Mark only one.*

- ☐ 2a. Scheduled exit visit/end of study → *End of form.*
- ☐ 2b. Death (Indicate date and cause if known.)
  - 2b1. Date of death: ☐☐ ☐☐☐ ☐☐ (*dd* *MMM* *yy*) *OR* ☐ date unknown
  - 2b2. Cause of death: ____________ *OR* ☐ cause unknown
  - → *Complete or update Adverse Experience Log.*
- ☐ 2c. Participant refused further participation, specify: ____________
- ☐ 2d. NOT APPLICABLE FOR THIS PROTOCOL
- ☐ 2e. Participant relocated, no follow-up planned
- ☐ 2f. Investigator decision, specify ____________
- ☐ 2g. Unable to contact participant
- ☐ 2h. NOT APPLICABLE FOR THIS PROTOCOL
- ☐ 2i. Inappropriate enrollment → *End of form.*
- ☐ 2j. Invalid ID due to duplicate screening/enrollment → *End of form.*
- ☐ 2k. Other, specify ____________
- ☐ 2l. Early study closure → *End of form.*
- ☐ 2m. Index Termination (Network Partners only) → *End of form.*

3 Was termination associated with an adverse experience? *yes* ☐ *no* ☐ *don't know* ☐ → *If no or don't know, end of form.*

3a. Record AE Log page number: ☐☐☐ (*page #*) *OR* Specify: *(Local language)* ____________ *(English)* ____________

Comments *(Local language)*: ____________

Comments *(English)*: ____________

This form should be completed for every enrolled participant at either the scheduled exit/end of study visit or when the participant is no longer participating in the study.

Item-specific Instructions:

**Item 1**	Document the date that the participant declares that he/she does not want to continue in the study. If the termination is due to death, indicate the date that the site is notified that the participant is deceased. A complete date is required.
**Item 2**	Mark only the primary reason for termination.
**Item 2a**	**Scheduled exit visit/end of study:** Only mark 2a if the participant completes the protocol-defined final visit.
**Item 2b1**	If date is recorded, at a minimum, the month and year are required.
**Item 2l**	**Early study closure:** Only mark 2l when instructed by SCHARP.
**Item 2m**	Only mark item 2m for Network Partners.
**Item 3a**	Record the page number of the Adverse Experience Log on which the AE was recorded. In situations where more than one AE is associated with termination, record the AE that most strongly influenced the decision to terminate. If termination is associated with a non-reportable AE, record the event on the “specify” line.

(HPTN 074) DF/Net 074 (PDL) 495

Page: ☐☐

Participant ID: ☐☐☐–☐☐☐☐–☐–☐☐

Site Number *Network Number* *Chk* *Cohort*

Form Completion Date: ☐☐ ☐☐☐ ☐☐

dd *MMM* *yy*

Protocol Deviation Log

**1**	Site awareness date: ☐☐ ☐☐☐ ☐☐ *dd MMM yy*	**2**	Deviation date: ☐☐ ☐☐☐ ☐☐ *dd MMM yy*
**3**	Has or will this deviation be reported to local IRB/EC? ☐ *Yes* ☐ *No*		
**4**	Has or will this deviation be reported to DAIDS as a critical event? ☐ *Yes* ☐ *No*		
**5**	Type of deviation: ☐☐ *deviation code (See back of form for code listing.)*		
**6**	Description of deviation *(Local language):* ____________		
	Description of deviation *(English):* ____________		
**7**	Plans and/or action taken to address the deviation *(Local language):* ____________		
	Plans and/or action taken to address the deviation *(English):* ____________		
**8**	Plans and/or action taken to prevent future occurrences of the deviation *(Local language):* ____________		
	Plans and/or action taken to prevent future occurrences of the deviation *(English):* ____________		
**9**	Deviation reported by (staff name): ____________		

Item-specific Instructions:

**Purpose**	This form documents and reports protocol deviations identified for study participants.
**General Information/ Instructions**	• Consult HPTN 074 Management Group (074mgmt@hptn.org) to confirm whether the event qualifies as reportable deviation.<br>• After confirmed, complete this form each time a protocol deviation is identified.<br>• Reportable protocol deviations are defined by the HPTN as individual incidents, trends or omissions that result in:<br>- Significant added risk to the participant<br>- Non-adherence to significant protocol requirements<br>- Significant non-adherence to GCP<br>• If a deviation needs to be reported but is not associated with a participant (for example, necessary test kits are not available on site), use a PTID that follows the format below on the PDL CRF:<br>- XXX-00000-0<br>- Enter your site DataFax ID number in the first 3 digits followed by zeros. Assign page numbers sequentially when completely new PDLs using this PTID.
**Page**	Number pages sequentially for each participant, starting with 01. Do not re-assign page numbers if a form is marked for deletion.
**Item 2**	Record the date the event occurred (start date).
**Item 5**	Record the two-digit category code that best describes the type of deviation. Use “99” (other) if none of the listed categories match. Describe the specifics of the deviation in item 6.
**Item 6**	Briefly describe the specific details of the deviation.
**Item 9**	Record name of site staff person who completed the form.

Code	Description
01	**Inappropriate enrollment:** The participant enrolled and not all eligibility requirements were met.
02	**Failure to follow trial randomization or blinding procedures:** Include instances where randomization procedures were not followed by site staff, or product blinding procedures were not followed by pharmacy staff.
03	**Study product management deviation:** Site staff did not instruct the participant to hold, permanently discontinue, or resume study product use per protocol requirements.
04	**Study product dispensing error:** The wrong study product was dispensed to a participant, or study product was dispensed to a participant on product hold. Do not include any information related to study product assignment (product codes) on this form.
05	**Conduct of non-protocol procedure:** A clinical or administrative procedure was performed that was not specified in the protocol, and was not covered under local standard of care practice.
06	**Breach of confidentiality:** Include potential and actual cases where participant confidentiality is breached. For example, a staff member puts a participant’s name on a case report form.
07	**Physical assessment deviation:** Examples include a protocolspecified exam or assessment consistently not being performed (a single missed exam during one participant visit would not be considered a reportable protocol deviation).
08	**Lab assessment deviation:** Examples include a protocol-specified laboratory assay consistently not being performed (a single missed assay during one participant visit would not be considered a reportable protocol deviation).
09	**Use of non-IRB/EC-approved materials:** Examples include use of ANY study-related material that has not received IRB or EC approval for use per site requirements.
10	**Informed assent/consent process deviation:** Examples include failure to accurately execute and/or document any part of the informed consent process.
99	**Other**

(HPTN 074) DF/Net 074 (DEM-1) 001

6 visit

Participant ID: 7 ptid

Site Number | Network Number | Chk | Cohort

Form Completion Date: 8 Date

dd MMM yy

Header [1]
Demographics [1]

Demographics

9 DEMstid

Thank you for being a part of our research. People live in different places, with different customs, cultures, sexual practices and beliefs. We hope to include people from different communities in our research. We respect all people, regardless of their differences. Not all questions we ask in our research will apply to you. Because we do not want to make assumptions, we ask the same questions of everyone. We want you to be comfortable in speaking with us. You do not have to answer any question that makes you uncomfortable.

Now I am going to ask you some questions about yourself. The answer to these questions will tell us more about who you are, such as your age and ethnicity (what cultural group you come from). I will also ask you about your sex and gender. Please feel free to ask any questions about things that you don't understand.

1	What is your date of birth?	10 DEMbthdt (dd MMM yy) **OR** ***If unknown, record age:*** 11 DEMage *years*
2	What is your sex at birth?	12 DEMsex: *Male* / *Female*
3	How do you identify your gender? *Mark all that apply.*	13 DEMgenm *Male*<br>14 DEMgenf *Female*<br>15 DEMgeno *Other, specify:*<br>*Local language:* ____<br>*English:* 16 DEMgenox
4	Are you currently a student?	17 DEMcs: *Yes* / *No*

VersionInitDate [1]

Version 18 19 VersDt | 20 formlang | Completed by: 21 InitDate *(initials/date)*

(HPTN 074) DF/Net 074 (PHT) 181

Visit Code: 6 visit

Participant ID: 7 ptid

Site Number | Network Number | Chk | Cohort

Header [1]

Partner HIV Test Results

HIV TEST RESULTS

PartnerHIVTest [1]

1 Specimen #1 Collection Date: 8 PHTcdt (dd MMM yy) OR Not done/ Not collected 9 PHTnd → *If not done/not collected at a visit where HIV testing is required by the protocol, provide reason in Comments.*

1a. Were any of the HIV test results positive or Reactive? 10 PHTpos No

PartnerHIVTest [2]

2 Specimen #2 Collection Date: 11 PHTcdt (dd MMM yy) OR Not done/ Not collected 12 PHTnd → *If not done/not collected at a visit where HIV testing is required by the protocol, provide reason in Comments.*

2a. Were any of the HIV test results positive or Reactive? 13 PHTpos No

3 Final HIV Status: 14 PHTfinal *Indeterminate*

Comments *(Local language)*: ______

Comments *(English)*: 15 PHTcomm

VersionInitDate [1]

Version 1 [illegible] 16 [illegible] 17 VersDt 18 formlang Completed by: 19 InitDate *(initials/date)*

Participant ID:

7 ptid

Site Number | Network Number | Chk | Cohort

Header [1]

Demographics

mographics [1]

5 What is the highest level of education you have completed?

8 DEMedu

- *No schooling*
- *Primary school, not complete*
- *Primary school, complete*
- *Secondary school, not complete*
- *Secondary school, complete*
- *Technical training, not complete*
- *Technical training, complete*
- *College or university, not complete*
- *College or university, complete*

6 What is your relationship status? Are you:

9 DEMmar

- *Married*
- *Living with a sexual partner but not married*
- *Separated*
- *Divorced*
- *Widowed*
- *Single*
- *Refuse to answer*

7 Have you been homeless at any time in the last 6 months?

10 DEMhl6mo

- *Yes*
- *No*

VersionInitDate [1

Version 3 11 12 VersDt | 13 formlang | Completed by: 14 InitDate (initials/date)

(HPTN 074) DF/Net 074 (DEM-3) 003

6 visit

Participant ID:

7 ptid

Site Number | Network Number | Chk | Cohort

Header [1]

Demographics

8 How would you describe your current living situation? *Mark all that apply.*

Demographics [1]

- [ ] 8 DEMlshor *8a. Live in a house that I own or rent*
- [ ] 9 DEMlspar *8b. Live in parent's home*
- [ ] 10 DEMlsrel *8c. Live in other relative's home (e.g, sibling, cousin, aunt and/or uncle)*
- [ ] 11 DEMlsrnt *8d. Rent a room or space in someone else's house or apartment*
- [ ] 12 DEMlsfre *8e. Stay with someone else for free (don't pay regular room and board)*
- [ ] 13 DEMlsst *8f. Live on the street, in a park, alley or abandoned building*
- [ ] 14 DEMlshlt *8g. Live in a homeless shelter*
- [ ] 15 DEMlsdif *8h. Stay at more than two different places a week*
- [ ] 16 DEMlstrn *8i. Live in a drug recovery or any transitional house*
- [ ] 17 DEMlsref *8j. Refuse to answer*
- [ ] 18 DEMlso *8k. Other place, specify:*

Local language: ______

English: 19 DEMlsox

9 Who lives with you? *Mark all that apply.*

- [ ] 20 DEMlvspo *9a. Spouse/partner/girl-boyfriend*
- [ ] 21 DEMlvch *9b. Own children*
- [ ] 22 DEMlvpar *9c. Parents*
- [ ] 23 DEMlvfam *9d. Other family members*
- [ ] 24 DEMlvfr *9e. Friends*
- [ ] 25 DEMlvoth *9f. Others (e.g., roommates, tenants)*
- [ ] 26 DEMlvalo *9g. Live alone*
- [ ] 27 DEMlvref *9h. Refuse to answer*

VersionInitDate [1]

Version 28 29 VersDt | 30 formlang | Completed by: 31 InitDate (initials/date)

(HPTN 074) DF/Net 074 (DEM-4) 004

6 visit

Participant ID:

7 ptid

Site Number | Network Number | Chk | Cohort

Header [1]

Demographics

10 At any time during the past 3 months, have you been unemployed?

Demographics [1]

8 DEMun3mo

- *Yes*
- *No*
- *Refuse to answer*

11 Which of the following best describes your current employment status? Are you:

9 DEMemp

- *Employed full-time*
- *Employed part-time*
- *Unemployed but seeking work*
- *Unemployed—not seeking work*
- *Retired*
- *Refuse to answer*

12 In the past month, how many days have you been working for pay? 10 DEMwrkdy *# days*

VersionInitDate [1]

Version 3 22- 11 V 12 VersDt 13 formlang **Completed by:** 14 InitDate *(initials/date)*

6 visit

(HPTN 074) DF/Net 074 (DVI) 007

Participant ID: 7 ptid

Site Number | Network Number | Chk | Cohort

Form Completion Date: 8 Date

dd MMM yy

Header [1]

Demographics - Vietnam

1 What does the participant report as his/her ethnicity/nationality? *Mark all that apply.*

Demographics [1]

- 9 DVIkinh — *1a. Kinh*
- 10 DVItay — *1b. Tay*
- 11 DVInung — *1c. Nung*
- 12 DVIsandi — *1d. San diu*
- 13 DVIhmong — *1e. H'Mong*
- 14 DVIsanch — *1f. San chi (Trail)*
- 15 DVIhoa — *1g. Hoa*
- 16 DVIdao — *1h. Dao (Man)*
- 17 DVIcaola — *1i. Cao Lan*
- 18 DVIngai — *1j. Ngai*
- 19 DVIthai — *1k. Thai*
- 20 DVIoth — *1l. Other, specify:* *Local language:* ______ *English:* 21 DVIothx

VersionInitDate [1]

Version 1 22 23 VersDt | 24 formlang | Completed by: 25 InitDate *(initials/date)*

(HPTN 074) DF/Net 074 (DIN) 009

6 visit

Participant ID:

7 ptid

Site Number | Network Number | Chk | Cohort

Form Completion Date: 8 Date

dd MMM yy

Header [1]

Demographics - Indonesia

1 What does the participant report as his/her ethnicity/nationality? *Mark all that apply.*

Demographics [1]

- ☐ 9 DINbetaw *1a. Betawi*
- ☐ 10 DINjawa *1b. Jawa*
- ☐ 11 DINsunda *1c. Sunda*
- ☐ 12 DINminan *1d. Minang*
- ☐ 13 DINbatak *1e. Batak*
- ☐ 14 DINbugis *1f. Bugis*
- ☐ 15 DINambon *1g. Ambon*
- ☐ 16 DINtiong *1h. Tionghoa*
- ☐ 17 DINoth *1i. Other, specify:* *Local language:* *English:* 18 DINothx

VersionInitDate [1]

Version 19 V 21 20 VersDt | 21 formlang | **Completed by:** 22 InitDate *(initials/date)*

(HPTN 074) DF/Net 074 (DUK) 011

6 visit

Participant ID:

7 ptid

Site Number | Network Number | Chk | Cohort

Form Completion Date: 8 Date

dd MMM yy

Header [1]

Demographics - Ukraine

1 What does the participant report as his/her ethnicity/nationality? *Mark all that apply.*

Demographics [1]

- 9 DUKukrai ☐ *1a. Ukrainian*
- 10 DUKrussi ☐ *1b. Russian*
- 11 DUKoth ☐ *1c. Other, specify:* *Local language:* ________ *English:* 12 DUKothx

Version 13 V 21- 14 VersDt | 15 formlang | VersionInitDate [1] Completed by: 16 InitDate *(initials/date)*

(HPTN 074) DF/Net 074 (IPT) 019

6 visit

Participant ID: 7 ptid

Site Number | Network Number | Chk | Cohort

Screening Visit Date: 8 Date

dd MMM yy

Header [1]

Index PTID Tracker

IndexTracker [1]

1 Was the participant's identity checked (e.g. fingerprint, unique identification card, etc.)? 9 IPTidck No

If yes, go to item 2.

1a. If no, reason: 10 IPTidx

2 Has this participant been seen before for HPTN 074? 11 IPTprev No *If no, end of form.*

2a. List all prior PTIDs associated with this participant:

	Site Number / Network Number / Chk / Cohort		Site Number / Network Number / Chk / Cohort
PTID #1:	12 IPTptid1	PTID #4:	15 IPTptid4
PTID #2:	13 IPTptid2	PTID #5:	16 IPTptid5
PTID #3:	14 IPTptid3		

VersionInitDate [1]

Version 1 17 18 VersDt 0- | 19 formlang | Completed by: 20 InitDate (initials/date)

(HPTN 074) DF/Net 074 (ISO-1) 020

6 visit

Participant ID: 7 ptid

Site Number | Network Number | Chk | Cohort

Screening Visit Date: 8 Date

dd MMM yy

Header [1]

Index Screening Outcome

1 Did the participant agree to have samples of blood collected at screening stored and used for future testing related to HIV infection?

ScreeningOutc [1]

9 ISOblsam
- Yes
- No

2 How was this participant recruited? *Mark all that apply.*

- 10 ISOrdi *2a. From another drug injector*
- 11 ISOrpart *2b. From an HPTN 074 participant*
- 12 ISOrdnet *2c. From a drug user outside of their network*
- 13 ISOrsoc *2d. Social media (e.g., Facebook) or other internet*
- 14 ISOrprnt *2e. Print media*
- 15 ISOrmedi *2f. TV or radio media*
- 16 ISOrharm *2g. Harm reduction sites/center*
- 17 ISOrhot *2h. Hot spots*
- 18 ISOrhivc *2i. HIV clinics*
- 19 ISOrmatc *2j. MAT clinics*
- 20 ISOrdloc *2k. Drug sales locations*
- 21 ISOroutr *2l. Outreach*
- 22 ISOroth *2m. Other, specify:*

Local language:

English: 23 ISOrothx

3 Did the participant enroll in the study?

24 ISOenr
- *Yes* → ***If yes, end of form. Do not submit page 2.***
- *No*

VersionInitDate [1]

Version 2 25 26 VersDt | 27 formlang | Completed by: 28 InitDate *(initials/date)*

6 visit

(HPTN 074) DF/Net 074 (ISO-2) 021

Participant ID:

7 ptid

Site Number | Network Number | Chk | Cohort

Header [1]

Index Screening Outcome

4 Why was the participant not enrolled in the study? *Mark all that apply.*

ScreeningOutc [1]

- 8 ISOnacti *4a. Current participation in an HIV prevention study or previous/current participation in an HIV vaccine study*
- 9 ISOnmed *4b. Serious and active medical or mental illness*
- 10 ISOnscrp *4c. Previously screened as a potential network partner of another index participant in this study*
- 11 ISOnpart *4d. Currently or previously a partner of an index participant*
- 12 ISOnadh *4e. Unwilling to adhere to study procedures*
- 13 ISOnref *4f. Withdrew consent/refused to participate*
- 14 ISOnunab *4g. Unable to recruit network partner within window after screening blood draw*
- 15 ISOnvl *4h. Viral load < 1,000 copies/mL*
- 16 ISOncrit *4i. Does not meet the criteria for active injection drug user per protocol*
- 17 ISOnhivn *4j. Not HIV positive*
- 18 ISOnshar *4k. Does not report sharing needles/syringes or drug solutions at least once in the last month*
- 19 ISOnscrn *4l. Did not complete screening procedures*
- 20 ISOnoth *4m. Other, specify:* *Local language:* ____________

English: 21 ISOnothx

VersionInitDate [1]

Version 2 22 23 VersDt | 24 formlang | Completed by: 25 InitDate *(initials/date)*

(HPTN 074) DF/Net 074 (PPT) 024

6 visit

Participant ID: 7 ptid

Site Number | Network Number | Chk | Cohort

Screening Visit Date: 8 Date

dd MMM yy

Header [1]

Partner PTID Tracker

PartnerTracker [1]

1 Was the participant's identity checked (e.g. fingerprint, unique identification card, etc.)? 9 PPTidck No

If yes, go to item 2.

1a. If no, reason: 10 PPTidx

2 Has this participant been seen before for HPTN 074? 11 PPTprev No

If no, end of form.

2a. List all prior PTIDs associated with this participant:

	Site Number / Network Number / Chk / Cohort		Site Number / Network Number / Chk / Cohort
PTID #1:	12 PPTptid1	PTID #4:	15 PPTptid4
PTID #2:	13 PPTptid2	PTID #5:	16 PPTptid5
PTID #3:	14 PPTptid3		

VersionInitDate [1]

Version 1 17 18 VersDt 19 formlang Completed by: 20 InitDate (initials/date)

6 visit

(HPTN 074) DF/Net 074 (PSO) 025

Participant ID: 7 ptid

Site Number | Network Number | Chk | Cohort

Screening Visit Date: 8 Date

dd MMM yy

Header [1]

Partner Screening Outcome

1 Did the participant agree to have samples of blood collected at screening stored and used for future testing related to HIV infection?

ScreeningOutc [1]

9 PSOblsam
- Yes
- No

2 Did the participant enroll in the study?

10 PSOoenr
- Yes → ***If yes, end of form.***
- No

3 Why was the participant not enrolled in the study? *Mark all that apply.*

- 11 PSOnhivn *3a. Any reactive or positive HIV rapid or HIV EIA test*
- 12 PSOnacti *3b. Current participation in an HIV prevention study or previous/current participation in an HIV vaccine study*
- 13 PSOnmed *3c. Serious and active medical or mental illness*
- 14 PSOnscrp *3d. Previously screened as a potential network member of another index participant in this study*
- 15 PSOnpart *3e. Previously named and enrolled as a partner of another index partner*
- 16 PSOnadh *3f. Unwilling to adhere to study procedures*
- 17 PSOnref *3g. Withdrew consent/refused to participate*
- 18 PSOncrit *3h. Does not meet the criteria for active injection drug user per protocol*
- 19 PSOnrel *3i. Could not confirm injection relationship with index*
- 20 PSOnscrn *3j. Did not complete screening procedures*
- 21 PSOnoth *3k. Other, specify:* *Local language:* ____

English: 22 PSOnothx

Version 23 24 VersDt | 25 formlang | VersionInitDate [1] Completed by: 26 InitDate *(initials/date)*

(HPTN 074) DF/Net 074 (IAI) 030

Visit Code: 6 visit

Participant ID: 7 ptid

Site Number | Network Number | Chk | Cohort

Visit Date: 8 Date

dd MMM yy

Header [1]

Index ART Initiation

ARTInitiation [1]

1 When did the participant indicate that s/he had initiated ART? 9 IAIindt

dd MMM yy

2 How was ART use determined? *Mark all that apply.*

10 IAIartsr *2a. Participant self-report*

11 IAIartcr *2b. Outside clinic report/documentation*

12 IAIarto *2c. Other, specify:* *Local language:* ______

English: 13 IAIartx

3 Is the participant willing/able to provide documentation?

14 IAIpdoc

Yes

No → ***If no, end of form.***

4 Record ART medications prescribed for the participant:

	ART Medication Code	Date Started (dd MMM yy)
4a.	15 IAImed	16 IAImdt
4b.	17 IAImed	18 IAImdt
4c.	19 IAImed	20 IAImdt
4d.	21 IAImed	22 IAImdt
4e.	23 IAImed	24 IAImdt

ARTInitiation [2]

ARTInitiation [3]

ARTInitiation [4]

ARTInitiation [5]

Comments *(Local language)*: ______

Comments *(English)*: 25 IAIcomm

VersionInitDate [1]

Version 1 26 27 VersDt 28 formlang **Completed by:** 29 InitDate *(initials/date)*

(HPTN 074) DF/Net 074 (SUT) 040

Visit Code: 6 visit

Participant ID: 7 ptid

Site Number | Network Number | Chk | Cohort

Visit Date: 8 Date

dd MMM yy

Header [1]

Substance Use Treatment Initiation

SubstanceUse [1]

1 When did the participant indicate that s/he had initiated substance use treatment? 9 SUTtxdt (dd MMM yy)

2 How was substance use treatment determined? *Mark all that apply.*

10 SUTdself *2a. Participant self-report*

11 SUTdouts *2b. Outside clinic report/documentation*

12 SUTdoth *2c. Other, specify:* *Local language:* ____

English: 13 SUTdothx

3 Is the participant willing/able to provide details about his/her substance use treatment?

14 SUTdetls *No* → ***If no, end of form.***

4 Record the types of substance use treatment the participant is involved in.

		Yes / No	If yes, date of most recent visit: dd MMM yy
4a.	Methadone	15 SUTmmt	24 SUTmmtdt
4b.	Buprenorphine	16 SUTbup	25 SUTbupdt
4c.	Other medication (MAT), specify: *Local Language:* *English:* 21 SUTomatx	17 SUTomat	26 SUTomadt
4d.	Detoxification	18 SUTdetox	27 SUTdtxdt
4e.	Community treatment/religious, specify: *Local Language:* *English:* 22 SUTctx	19 SUTct	28 SUTctdt
4f.	Other specify: *Local Language:* *English:* 23 SUTothx	20 SUToth	29 SUTothdt

Comments *(Local language)*: ____

Comments (English): 30 SUTcomm

VersionInitDate [1]

Version 2 31 32 VersDt | 33 formlang | Completed by: 34 InitDate *(initials/date)*

6 visit

Participant ID: 7 ptid

Site Number | Network Number | Chk | Cohort

Assessment Date: 8 Date

dd MMM yy

Header [1]

Baseline Behavior Assessment

A broad range of participants are taking part in this study. So some questions may not apply to you. We ask the same questions of all participants.

In this interview, you will be asked about your sexual behaviors and possible alcohol and drug use. Some of the questions may seem very personal. Please remember that all of your answers will be kept private. Your responses are linked only to your study number, not to your name.

***We need you to answer these questions honestly.** We do not judge about alcohol or drug use. We make no judgments about how you have sex or the number of times or with whom.*

These questions help us understand the type of behaviors our participants are doing while on study. Your honesty will help us understand these differences in behaviors.

HIV TESTING HISTORY

BaseBehavAssess [1]

1 Prior to participating in this study, did you ever have an HIV test? 9 BBAhivt — No → ***If no, go to item 2.***

1a. How many times have you had an HIV test in your lifetime? 10 BBAhivnm *# of times*

1b. When was your most recent HIV test? 11 BBAhivdt *dd MMM yy*

1c. What was the result of that test? 12 BBAhivr *Don't know*

INCARCERATION

2 In the last 3 months, how many different times were you in...

2a. Jail/prison? 13 BBAjail *# of times*

2b. Involuntary detoxification/detention? 14 BBAdetox *# of times*

2c. Other incarceration, specify: 15 BBAoinc *# of times*

Local language: ______________ *English:* 16 BBAoincx

VersionInitDate [1]

Version 2.5 | 17 | 18 VersDt | 19 formlang | **Completed by:** 20 InitDate *(initials/date)*

Participant ID:

7 ptid

Site Number | Network Number | Chk | Cohort

Header [1]

Baseline Behavior Assessment

ALCOHOL USE (Audit C) *Answer questions to reflect your behavior over the past 3 months.*

aseBehavAssess [1

3 How often do you have a drink containing alcohol?

8 BBAafreq

2–3 times a week

4 or more times a week

4 How many standard drinks containing alcohol do you have on a typical day?

9 BBAanum

7 to 9

10 or more

5 How often do you have six or more drinks on one occasion?

10 BBA6drnk

Weekly

Daily or almost daily

NON-INJECTION DRUG USE

6 In the **last 3 months**, did you... *Yes* *No*

6a. Use marijuana? 11 BBAnimj

6b. Use stimulants (cocaine, methamphetamines)? 12 BBAnist

6c. Use opiates (heroin, opium)? 13 BBAniop

6d. Use other drugs *(as indicated by sites)*, specify: 14 BBAnioth

Local language: ____________________

English: 15 BBAniox

7 When was the last time you used marijuana, amphetamines, methadone, opiates or benzodiazepines that you did not inject? 16 BBAnidt (*dd MMM yy*) OR *Never* 17 BBAninev

VersionInitDate [1

Version 2 18 5- 19 VersDt | 20 formlang | **Completed by:** 21 InitDate *(initials/date)*

Baseline Behavior Assessment

INJECTION DRUG USE

aseBehavAssess [1

8	How old were you when you first injected drugs?	8 BBAiage years
9	What is the longest time you have not injected drugs, voluntarily (e.g. when you were not incarcerated), since you started using injection drugs?	months 9 BBAiclnm; days 10 BBAiclnd; years 11 BBAiclny
10	In the **last 3 months**, which of the following did you inject?	

		Yes / No			Yes / No
10a.	Amphetamines	12 BBAiamp	10i.	Ketamine	20 BBAiket
10b.	Methamphetamines	13 BBAimamp	10j.	Homemade opiods	21 BBAihop
10c.	Cocaine	14 BBAicoc	10k.	Short and long action stimulants	22 BBAisas
10d.	Heroin	15 BBAiher	10l.	Dezomorphine	23 BBAidez
10e.	Opium	16 BBAiop	10m.	Other, specify:	24 BBAioth
10f.	Buprenorphine	17 BBAibup		*Local language:* ______	
10g.	Methadone	18 BBAimeth		*English:* 25 BBAiotx	
10h.	Benzodiazepines	19 BBAibenz			

11	In **the last month**, on how many days did you inject drugs?	26 BBAinjd # of days
12	In **the last month**, on days that you injected, how many times a day did you usually inject drugs?	27 BBAinjt # of times a day
13	When was the last day you injected (including today)?	28 BBAidt (dd MMM yy) OR 29 BBAima # of months ago

/ersionInitDate [1

(HPTN 074) DF/Net 074 (BBA-4) 074

6 visit

Participant ID:

7 ptid

Site Number | Network Number | Chk | Cohort

Header [1]

Baseline Behavior Assessment

SHARING

Often people share syringes and other equipment because they are in a rush, they are concerned about the police, feeling sick or for a variety of other reasons.

aseBehavAssess [1

		Yes / No / Don't know
14	Have you shared rinse water in **the last 3 months**?	8 BBAsrw
	If no, go to item 15.	
	14a. The last time you injected, did you share rinse water?	9 BBAsrwls
15	Have you shared a cooker/container in **the last 3 months**?	10 BBAscc
	If no, go to item 16.	
	15a. The last time you injected, did you share a cooker/container?	11 BBAsccls
16	Have you shared filter cotton in **the last 3 months**?	12 BBAsfc
	If no, go to item 17.	
	16a. The last time you injected, did you share filter cotton?	13 BBAsfcls

CLEANING

17 How often do you clean your needle before injecting?

14 BBAclnf — *Never*

		Yes / No / Don't know
18	The last time you injected, did you clean the needle before you injected?	15 BBAndlc
19	The last time you injected, did you use a new needle?	16 BBAndln

20 How often do you clean your needle after injecting?

17 BBAclna — *Never*

		Yes / No / Don't know
21	The last time you injected, did you clean the needle after you injected?	18 BBAclnls

/ersionInitDate [1

Version 19, 20 VersDt | 21 formlang | Completed by: 22 InitDate *(initials/date)*

Participant ID:

7 ptid

Site Number | Network Number | Chk | Cohort

Header [1]

Baseline Behavior Assessment

PRE-FILLING AND BACKLOADING SYRINGES

aseBehavAssess [1

22	The last time you injected, did you use a pre-filled syringe?	8 BBApfs	Don't know
23	The last time you injected, did you inject drugs that were frontloaded or backloaded into the syringe or needle that you used?	9 BBAfbs	Don't know
24	In **the last 3 months**, how many different people did you use injection drugs with?	10 BBApeop # of people	**If 000, go to item 30.**
25	In **the last 3 months**, how many times did you even once pass on a needle or syringe to someone else after you used it?	11 BBApass # of times	**If 000, go to item 27.**
26	In **the last 3 months**, with how many different people did you pass on a needle or syringe after you had used it?	12 BBApassn # of people	
27	In **the last 3 months**, how many times did you ever, even once, use a needle or syringe after someone else used it?	13 BBAused # of times	**If 000, go to item 29.**
28	In **the last 3 months**, with how many different people did you use a needle or syringe after someone else used it?	14 BBAusedn # of people	

29 In **the past 3 months**, when you shared syringes, how often did you inject last?

15 BBAilast

Sometimes

I have not shared in the past 3 months

30 In **the last 3 months**, did you get your new needles/syringes from a…

30a. 16 BBAnonew *I did not get new needles in the last 3 months* → **Go to item 32 on page 6.**

		Yes / No
30b.	Pharmacy or health center?	17 BBApharm
30c.	Needle seller?	18 BBAsellr
30d.	Needle exchange?	19 BBAnexch
30e.	Other drug injectors?	20 BBAodinj
30f.	Other, specify?	21 BBAosrc

Local language: ______________________ *English:* 22 BBAosrcx

VersionInitDate [1

Version 2 23 24 VersDt | 25 formlang | **Completed by:** 26 InitDate *(initials/date)*

Participant ID:

7 ptid

Site Number | Network Number | Chk | Cohort

Header [1]

Baseline Behavior Assessment

PRE-FILLING AND BACKLOADING SYRINGES

BaseBehavAssess [1

31 In **the last 3 months**, when you obtained new needles, typically, how many did you buy at one time?

8 BBAbuy *# of needles*

DRUG TREATMENT PROGRAMS

Indicate which types of treatment you have participated in.

32 Have you ever participated in any type of drug treatment program?

9 BBAtx *No* → *If no, go to item 36 on page 7.*

33 Methadone maintenance or any other medication assisted treatment program (MMT/MAT)?

10 BBAmat *No* → *If no, go to item 34 on page 7.*

33a. What type of MMT/MAT program have you been on? ***Mark all that apply.***

11 BBAtmeth *33a1. Methadone*

12 BBAtbup *33a2. Buprenorphine*

13 BBAtoth *33a3. Other, specify:*

(Local language)

(English) 14 BBAtothx

33b. How many times have you enrolled in MMT/MAT?

15 BBAmatt *# of times*

33c. What is the longest period of time you have been in MMT/MAT?

16 BBAmatm *months* 17 BBAmatd *days*

33d. Have you participated in MMT/MAT in **the last 3 months**?

18 BBAmat3m *No* → *If no, go to item 34 on page 7.*

33e. What type of MMT/MAT program have you been on in **the last 3 months**? ***Mark all that apply.***

19 BBAtm3m *33e1. Methadone*

20 BBAtb3m *33e2. Buprenorphine*

21 BBAto3m *33e3. Other, specify:*

(Local language)

(English) 22 BBAto3mx

33f. Are you currently in MMT/MAT?

23 BBAmatc *No*

→ ***If yes, complete the Substance Use Treatment Initiation CRF.***

VersionInitDate [1

Version 24 25 VersDt 26 formlang **Completed by:** 27 InitDate *(initials/date)*

(HPTN 074) DF/Net 074 (BBA-7) 077

6 visit

Participant ID:

7 ptid

Site Number | Network Number | Chk | Cohort

Header [1]

Baseline Behavior Assessment

DRUG TREATMENT PROGRAMS

aseBehavAssess [1

**34**	A health care provider supported drug detoxification program?	8 BBAhcpd No → *If no, go to item 35.*
	34a. How many times have you enrolled in detoxification?	9 BBAdtxt # of times
	34b. What is the longest period of time you have been in detoxification?	10 BBAmatlm months 11 BBAmatld days
	34c. Have you participated in detoxification in the last 3 months?	12 BBAdtx3m No → *If no, go to item 35.*
	34d. Are you currently in detoxification?	13 BBAdtxc No
**35**	Any type of other drug treatment program (e.g. community treatment/religious)?	14 BBAotrt No → *If no, go to item 36.*
	35a. How many times have you enrolled in any other type of drug treatment?	15 BBAotrtt # of times
	35b. What is the longest period of time you have been in this type of program?	16 BBAotrtm months 17 BBAotrtd days
	35c. Have you participated in this type of program in the last 3 months?	18 BBAot3m No → *If no, go to item 36.*
	35d. Are you currently in this type of program?	19 BBAotrtc No

SEXUAL BEHAVIOR QUESTIONS

The next few questions are about sexual behavior. These are very personal questions. Please remember that all the information you give us is confidential. We ask that you answer the questions as honestly as you can.

**36**	In **the last 3 months**, did you have vaginal or anal sex?	20 BBAsex3m No → *If no, go to item 42 on page 8.*
**37**	In **the last month**, how many different **female** sex partners have you had?	21 BBAsexf # of female sex partners
**38**	In **the last month**, how many different **male** sex partners have you had?	22 BBAsexm # of male sex partners

VersionInitDate [1

Version 2 23 24 VersDt 25 formlang Completed by: 26 InitDate (initials/date)

(HPTN 074) DF/Net 074 (BBA-8) 078

6 visit

Participant ID:

7 ptid

Site Number | Network Number | Chk | Cohort

Header [1]

Baseline Behavior Assessment

aseBehavAssess [1

39 Do you have a main partner such as a husband/wife or boyfriend/girlfriend? 8 BBAmp No → *If no, go to item 41.*

39a. Is your main partner male or female?

9 BBAmpgen *Other, specify:* *(Local language)* ____

(English) 10 BBAmpgnx

40 In **the last month**, how many times did you have vaginal or anal sex with your main partner? 11 BBAsexmp *# of times* → *If 000, go to item 41.*

40a. How many of these times did you (or your partner) use a condom? 12 BBAmpcon *# of times*

41 In **the last month**, how many times did you have vaginal or anal sex with someone other than a main partner? 13 BBAsexop *# of times* → *If 000, go to item 42.*

41a. How many of these times did you (or your partner) use a condom? 14 BBAopcon *# of times*

Next I am going to ask about your sex trade (paid or paying) partners. By sex trade partners, I mean people that you had oral, vaginal or anal sex with in exchange for money, drugs, food or a place to stay.

42 In **the last month**, how many times did you give sex partners money or drugs in exchange for sex? 15 BBAexchy *# of times*

43 In **the last month**, how many times did a sex partner give you money or drugs in exchange for sex? 16 BBAexchp *# of times*

VersionInitDate [1

Version 17 18 VersDt 19 formlang **Completed by:** 20 InitDate *(initials/date)*

(HPTN 074) DF/Net 074 (IBH-1) 085

6 visit

Participant ID: 7 ptid

Site Number | Network Number | Chk | Cohort

Assessment Date: 8 Date

dd MMM yy

Header [1]

Index Baseline HIV Care and ART

The next few questions are about anti-HIV medicines called antiretroviral therapy that you may be taking. These questions are not about drugs you may be taking to treat substance use. We need to know what is really happening, not what you think we "want to hear." Many people find it hard to take their medications all of the time.

IndexBaselnHIV [1

1 When did you first find out that you were HIV positive? 9 IBHhivdt

dd MMM yy

2 With whom have you shared that you are living with HIV? *Mark all that apply.*

- 10 IBHspous *2a. Spouse/partner/girl-boyfriend*
- 11 IBHchild *2b. Own children*
- 12 IBhparen *2c. Parents*
- 13 IBHofm *2d. Other family members*
- 14 IBHfrnds *2e. Friends*
- 15 IBHshoth *2f. Others*
- 16 IBHnoone *2g. No one*
- 17 IBHref *2h. Refuse to answer*

3 How many of your drug partners (the people you inject drugs with) know that you have HIV?

18 IBHptnr

- *All of my partners*
- *More than half of my partners*
- *Less than half of my partners*
- *None of my partners*

VersionInitDate [1

Version 19 20 VersDt

21 formlang

Completed by: 22 InitDate ________ *(initials/date)*

Index Baseline HIV Care and ART

4 Who is the most supportive in helping you with your HIV care and treatment?

ndexBaselnHIV [1

8 IBHsuprt

- *Health care providers (such as doctors or nurses)*
- *Your spouse/live-in partner/long-term partner*
- *Casual sexual partner (i.e., friends or associates who you just met and had sex with)*
- *Injecting partners*
- *Commercial sex workers*
- *Your mother and father*
- *Your in-laws*
- *Other family members*
- *Priests/religious leaders*
- *Your neighbors or community members*
- *Friends*
- *Your employers*
- *Other, specify:* *(Local language)* ____________
  (English) 9 IBHsothx

5 How often have you talked to your family or support person about your HIV medications?

10 IBHtalk

One time

Never

6 Since you found out you were living with HIV, have you seen an HIV clinician? 11 IBHcln *No* → ***If no, go to item 8 on page 3.***

6a. If yes, on what date was your most recent visit? 12 IBHclndt (*dd* *MMM* *yy*)

7 Has any HIV clinician ever told you that you should initiate ART treatment for your own health? 13 IBHainit *No*

/ersionInitDate [1

Version 14 15 VersDt 16 formlang Completed by: 17 InitDate *(initials/date)*

(HPTN 074) DF/Net 074 (IBH-3) 087

6 visit

Participant ID:

7 ptid

Site Number | Network Number | Chk | Cohort

Header [1]

Index Baseline HIV Care and ART

IndexBaseInHIV [1

8	Have you ever visited an HIV clinic or pharmacy with the intention of starting ART?	8 IBHastrt	No
9	Have you ever been on ART treatment?	9 IBHatrt	No
10	Are you currently taking ART medications? *If yes, complete Index ART Initiation CRF.*	10 IBHacurr	No *If no, go to Item 16 on page 5.*
11	We know that it is very difficult to take your HIV medications all the time. When was the last time you missed taking any doses of your ART medication? 11 IBHalast	*Never missed taking medications in past 3 months* *End of form.*	
12	In **the last month**, on about how many days did you miss at least one tablet?	12 IBHadays days	
13	Did you miss at least one tablet in **the last four days**?	13 IBHa4day	No *If no, go to Item 15 on page 4.*
	13a. In **the last four days**, on how many days did you miss at least one tablet?	14 IBHa4num days	
14	Did you miss at least one tablet **yesterday**?	15 IBHayest	No

VersionInitDate [1

Version 3 16 17 VersDt | 18 formlang | Completed by: 19 InitDate (initials/date)

Participant ID:

7 ptid

Site Number | Network Number | Chk | Cohort

Index Baseline HIV Care and ART

15 The last time you missed at least one tablet, which of these best describes the reason why? *Mark only one.* ***End of form.***

9 IBHarsn

- *I forgot*
- *I did not have the medication with me at the time I needed to take it*
- *I did not want person(s) nearby to see me taking the medication*
- *I was trying to avoid side effects*
- *I felt healthy*
- *I was drunk*
- *I am not engaging in any behaviors that can transmit HIV to others*
- *I do not believe the medicines are beneficial*
- *Not applicable*
- *Other, specify:* *(Local language)* ____________ *(English)* 10 IBHarsx

VersionInitDate [1]

Version 11 12 VersDt | 13 formlang | Completed by: 14 InitDate *(initials/date)*

Participant ID:
7 ptid
Site Number Network Number Chk Cohort

Index Baseline HIV Care and ART

		Agree	Disagree	Not relevant/ Not Applicable
16	Sometimes people have trouble starting HIV treatment (ART) because of personal situations or circumstances. What are the reasons you have not started taking ART?			
	16a. You don't think that you need it.	9 IBHpneed		
	16b. You don't have time to go to the clinic.	10 IBHptime		
	16c. You don't have the energy or motivation to go to the clinic.	11 IBHpenrg		
	16d. You missed an appointment, so it is difficult to go back.	12 IBHpappt		
	16e. You have been too sick to go to the clinic.	13 IBHpsick		
	16f. Your drug use got in the way of going to the clinic.	14 IBHpdrug		
	16g. You are worried about the unwanted side effects or complications.	15 IBHpsaff		
	16h. You don't understand when you are supposed to take each pill.	16 IBHpunds		
	16i. You sold or lost your medicine or your medicine was stolen.	17 IBHplost		
	16j. Your drinking (alcohol) got in the way of you going to the clinic.	18 IBHpalc		
	16k. You had to wait too long, so you left without being seen.	19 IBHpwait		
17	Sometimes people find it difficult to start taking ART because of their family or friends. What are the reasons you have not started taking ART?	Agree	Disagree	Not relevant/ Not Applicable
	17a. You don't have enough support from family or friends.	20 IBHfnosp		
	17b. You don't want others to know you have HIV (e.g., see me take medication).	21 IBHfknow		

6 visit

ndexBaselnHIV [1

No data recorded on this page 8 IBH6ndr

Participant ID:

7 ptid

Site Number *Network Number* *Chk* *Cohort*

Header [1]

Index Baseline HIV Care and ART

		Agree	*Disagree*	*Not relevant/ Not Applicable*
**18**	Many people find it difficult to start taking ART, even if they have previously tried, because of challenges in the health systems/health care process. What were the reasons you have not started taking ART?			
	18a. You do not have the necessary materials (e.g. paperwork, identification, test results) needed by the clinic.	9 IBHhmat		
	18b. ART is too expensive.	10 IBHhexp		
	18c. ART was not available.	11 IBHhna		
	18d. HIV care provider would not continue your medicine or prescription.	12 IBHhmed		
	18e. You do not know how or where to get ART.	13 IBHhget		
	18f. You find transport/travel to the clinic is difficult.	14 IBHhtran		
	18g. You have been traveling.	15 IBHhtrav		
	18h. You have been in jail or incarcerated.	16 IBHhjail		
	18i. You are scared of being arrested for your drug use.	17 IBHharst		
	18j. You will need to register with the government as being a drug user.	18 IBHhgov		

19 Are there any other reasons you find it difficult to start taking ART?

Local language: ______________________________

English: 19 IBHhothx

/ersionInitDate [1

Version 20 21 VersDt 22 formlang **Completed by:** 23 InitDate *(initials/date)*

(HPTN 074) DF/Net 074 (IE) 091

6 visit

Participant ID: 7 ptid

Site Number | Network Number | Chk | Cohort

Enrollment Date: 8 Date

dd MMM yy

Header [1]

Index Enrollment

IndexEnroll [1]

1	Did the participant agree to have their samples of blood collected at enrollment used for future testing related to HIV infection?<br>9 IEcolbl<br>*Yes*<br>*No*
2	Did the participant agree to have their samples of urine collected at enrollment used for future testing related to drug use?<br>10 IEcolurn<br>*Yes*<br>*No*
3	To which study arm is the participant assigned?<br>11 IEarm<br>*Standard of care*<br>*Intervention*

VersionInitDate [1]

Version 1 12 13 VersDt | 14 formlang | Completed by: 15 InitDate *(initials/date)*

(HPTN 074) DF/Net 074 (PE) 095

6 visit

Participant ID: 7 ptid

Site Number | Network Number | Chk | Cohort

Enrollment Date: 8 Date

dd MMM yy

Header [1]

Partner Enrollment

1 Did the participant agree to have their samples of blood collected at enrollment used for future testing related to HIV infection?

PartnerEnroll [1]

9 PEcolbl

- Yes
- No

2 Did the participant agree to have their samples of urine collected at enrollment used for future testing related to drug use?

10 PEcolurn

- Yes
- No

3 At which index visit was this network partner enrolled? Index Visit Code: 11 PEienrvs

3a. Index enrollment date: Date: 12 PEienrdt

dd MMM yy

VersionInitDate [1]

Version 13 14 VersDt | 15 formlang | Completed by: 16 InitDate (initials/date)

(HPTN 074) DF/Net 074 (INS) 111

Visit Code: 6 visit

Participant ID: 7 ptid

Site Number | Network Number | Chk | Cohort

Visit Date: 8 Date

dd MMM yy

Header [1]

Index Network Status

IndexNetwork [1]

1	How many total injection partners are listed on the Social/Sexual Network List?	9 INSipnum # of partners
2	How many new injection partners were added to the partner list as of this visit? By new, we mean they had not been in the list prior to this visit.	10 INSnpnum # of new partners
3	Is a new partner enrolling at this visit?	11 INSpnenr No
	3a. Does the index currently have an enrolled partner with whom they are currently (within the last month) sharing needles, water, cookers, etc?	12 INSpenr No

If both 3 and 3a are no, end of form.

4 List the enrolled partners with whom the participant has shared needles, water, cookers etc. in the last 3 months (include partners enrolled at this visit).

	Partner's Initials or Nickname:	Participant ID: Site Number / Network Number / Chk / Cohort
4a.	13 INSpart	14 INSpid
4b.	15 INSpart	16 INSpid
4c.	17 INSpart	18 INSpid
4d.	19 INSpart	20 INSpid
4e.	21 INSpart	22 INSpid
4f.	23 INSpart	24 INSpid
4g.	25 INSpart	26 INSpid
4h.	27 INSpart	28 INSpid
4i.	29 INSpart	30 INSpid

IndexNetwork [2] IndexNetwork [3] IndexNetwork [4] IndexNetwork [5] IndexNetwork [6] IndexNetwork [7] IndexNetwork [8] IndexNetwork [9]

Comments *(Local Language)*: ____

Comments *(English)*: 31 INScomm

VersionInitDate [1]

Version 32 V 20 33 VersDt | 34 formlang | Completed by: 35 InitDate *(initials/date)*

(HPTN 074) DF/Net 074 (FUV) 120

Visit Code: 6 visit

Participant ID: 7 ptid

Site Number | Network Number | Chk | Cohort

Visit Date: 8 Date

dd MMM yy

Header [1]

Follow-up Visit

Follow-upVisit [1]

1 Is this an interim visit? 9 FUVinvis No → *If no, go to item 2.*

1a. Reason for interim visit. ***Mark all that apply.***

- 10 FUVrae *1a1. Report a new AE(s)*
- 11 FUVrsoim *1a2. Report a social impact*
- 12 FUVhivlt *1a3. HIV testing*
- 13 FUVninjp *1a4. New injection partner*
- 14 FUVrlabw *1a5. Repeat laboratory work*
- 15 FUVaoth *1a6. Other, specify:*
  - *(Local language)* ______
  - *(English)* 16 FUVaothx

1b. Besides this form, what other DataFax forms were completed at this visit? ***Mark all that apply.***

- 17 FUVpht *1b1. Partner HIV Test Results*
- 18 FUViai *1b2. Index ART Initiation*
- 19 FUVsut *1b3. Substance Use Treatment Initiation*
- 20 FUVuds *1b4. Urine Drug Screen*
- 21 FUVyss *1b5. Specimen Storage*
- 22 FUVcdf *1b6. CD4+/Viral Load Results*
- 23 FUVae *1b7. Adverse Experience Log (new)*
- 24 FUVsil *1b8. Social Impact Log (new)*
- 25 FUVboth *1b9. Other, specify:*
  - *(Local language)* ______
  - *(English)* 26 FUVbothx

2 At this visit, how many **new** Adverse Experiences (AEs) have been reported? 27 FUVaepg → ***Complete a separate AE Log page for each AE. If none, enter 00.***

3 At this visit, how many **new** social impacts have been reported? 28 FUVsilpg → ***Complete a separate Social Impact Log page for each event. If none, enter 00.***

VersionInitDate [1]

Version 29 2 30 VersDt | 31 formlang | Completed by: 32 InitDate *(initials/date)*

(HPTN 074) DF/Net 074 (SIL) 151

Page: 6 visit

Participant ID:

7 ptid

Site Number | Network Number | Chk | Cohort

Header [1]

Social Impact Log

SocialImpctLog [1

1	Concisely describe social impact:	8 SILsumx
2	Onset date:	9 SILondt (dd MMM yy)
3	Reported at visit:	10 SILatvis
4	Social impact code:	11 SILcode → ***See back for definitions of Social Impact Codes.***

VersionInitDate [1

Version 12 13 VersDt | 14 formlang | Completed by: 15 InitDate (initials/date)

(HPTN 074) DF/Net 074 (SIA-1) 152

Visit Code: 6 visit

Participant ID: 7 ptid

Site Number | Network Number | Chk | Cohort

Contact Date: 8 Date

dd MMM yy

Header [1]

Social Impact Assessment

SocialImpact [1]

1	Because of your participation in this study, did anything negative or bad happen to you that you have not reported to us already?	9 SIAnegnr	No → *If no, go to item 4.*
2	Because of your participation in this study, have you...		
	2a. been arrested or had trouble with the police or other legal problems?	10 SIAlg	No → *If no, go to item 2b.*
	2a1. If yes how many times?	11 SIAlgnum # of times	
	2b. had trouble getting or keeping housing?	12 SIAhs	No → *If no, go to item 2c.*
	2b1. If yes how many times?	13 SIAhsnum # of times	
	2c. had trouble getting or keeping a job or trouble with income or economic support?	14 SIAj	No → *If no, go to item 2d.*
	2c1. If yes how many times?	15 SIAjnum # of times	
	2d. had trouble getting health care or with health insurance?	16 SIAhe	No → *If no, go to item 2e.*
	2d1. If yes how many times?	17 SIAhenum # of times	
	2e. had personal trouble with friends, family, or acquaintances?	18 SIAtr	No → *If no, go to item 2f.*
	2e1. If yes how many times?	19 SIAtrnum # of times	
	2f. had any other type of problem?	20 SIAop	No → *If no, go to item 3.*
	Local language: ____________	*specify:* *English:* 21 SIAopx	
3	Indicate the total number of impacts:	22 SIAnum # of impacts	*Complete a separate Social Impact Log (SIL) for each impact.*
4	Has your participation in this study had any positive or beneficial impact on your life?	23 SIApos	Don't know<br>*If no, end of form. Do not fax page 2.*

VersionInitDate [1]

Version 2 24 25 VersDt | 26 formlang | Completed by: 27 InitDate *(initials/date)*

(HPTN 074) DF/Net 074 (SIA-2) 153

Visit Code: 6 visit

Participant ID:

7 ptid

Site Number | Network Number | Chk | Cohort

Header [1]

Social Impact Assessment

SocialImpact [1]

		Yes	No
5	Because of your participation in this study, have you experienced...		
5a.	Employment improvement?	8 SIAei	
5b.	Financial improvement?	9 SIAfi	
5c.	Reduction in drug use?	10 SIAredd	
5d.	Reduction in cravings/withdrawal?	11 SIAredc	
5e.	Gained knowledge?	12 SIAgknow	
5f.	Life improvement?	13 SIAli	
5g.	Physical health improvement?	14 SIAphi	
5h.	Improved relationships?	15 SIAreli	
5i.	Reduced stigma?	16 SIAredsg	
5j.	Improved mental health?	17 SIAmhi	
5k.	Other, specify:	18 SIAoi	

Local language: ____________

English: 19 SIAoix

VersionInitDate [1]

Version 20 21 VersDt | 22 formlang | Completed by: 23 InitDate *(initials/date)*

(HPTN 074) DF/Net 074 (STG-1) 155

Visit Code: 6 visit

Participant ID: 7 ptid

Site Number | Network Number | Chk | Cohort

Contact Date: 8 Date

dd MMM yy

Header [1]

Social Support and Stigma Questionnaire

In this section, I am going to read you statements about how you may feel about yourself, your injection drug use, and your HIV/AIDS infection. I would like you to tell me if you agree strongly, agree, disagree, or disagree strongly with each statement.

INTERNALIZED STIGMA AND SHAME

		Agree strongly / *Agree* / *Disagree* / *Disagree strongly*
1	I think less of myself or I have felt ashamed because:	ocialSupStigma [1
	1a. I inject drugs.	9 STGissid
	1b. I am living with HIV.	10 STGisswh

EXPERIENCED STIGMA

		Agree strongly / *Agree* / *Disagree* / *Disagree strongly*
2	I have become isolated from my family because:	
	2a. I inject drugs.	11 STGesid
	2b. I am living with HIV.	12 STGeswh

ANTICIPATED STIGMA

		Agree strongly / *Agree* / *Disagree* / *Disagree strongly*
3	I fear that if I disclosed my HIV status to my family, they would exclude me from usual family activities.	13 STGfamex
4	I fear that if I disclosed my HIV status to others, I would find it hard to find work.	14 STGfindw
5	I fear that if I go to an MMT or buprenorphine clinic to treat my substance use I will be treated poorly.	15 STGmbtp
6	I fear that if I go to an HIV clinic I will be treated poorly because I inject drugs.	16 STGhivtp
7	I do not feel comfortable talking to health care providers as I feel they would judge me.	17 STGhcpjm

/ersionInitDate [1

Version 18 19 VersDt 20 formlang Completed by: 21 InitDate (initials/date)

(HPTN 074) DF/Net 074 (STG-2) 156

Visit Code: 6 visit

Participant ID: 7 ptid

Site Number | Network Number | Chk | Cohort

Header [1]

Social Support and Stigma Questionnaire

8 Over the last 2 weeks, how often have you been bothered by any of the following problems?

ocialSupStigma [1

	Not at all	Several days	More than half of the days	Nearly every day
8a. Little interest or pleasure in doing things	8 STGlilpl			
8b. Feeling down, depressed or hopeless	9 STGdprss			
8c. Trouble falling or staying asleep or sleeping too much	10 STGsleep			
8d. Feeling tired or having little energy	11 STGtired			
8e. Poor appetite or overeating	12 STGeatng			
8f. Feeling bad about yourself—or that you are a failure or have let yourself or your family down	13 STGflbad			
8g. Trouble concentrating on things, such as reading the newspaper or watching television	14 STGtrblc			
8h. Moving or speaking so slowly that other people have noticed; or the opposite—being so fidgety or restless that you have to be moving around a lot more than usual	15 STGmovng			
8i. Thoughts that you would be better off dead or of hurting yourself in some way	16 STGthght			

9 People sometimes look to others for companionship, assistance, or other types of support. Please tell me how often each of the following kinds of support are available to you if you need them.

9a. Someone to have a good time with:

17 STGgoodt *Most of the time*

9b. Someone to get together with for relaxation:

18 STGrelax *Most of the time*

9c. Someone to do something enjoyable with:

19 STGenjoy *Most of the time*

/ersionInitDate [1

Version 1. 20 21 VersDt | 22 formlang | Completed by: 23 InitDate *(initials/date)*

(HPTN 074) DF/Net 074 (ITB) 158

Visit Code: 6 visit

Participant ID: 7 ptid

Site Number | Network Number | Chk | Cohort

Contact Date: 8 Date

dd MMM yy

Header [1]

Index Treatment Belief Questionnaire

		Agree strongly / Agree / Disagree / Disagree strongly
1	If you have side effects, you should continue to take your HIV treatment.	9 ITBhivse
2	HIV treatment is safe.	10 ITBhivsf
3	HIV treatment is effective (by effective we mean, does it work).	11 ITBhivef
4	If you miss your medication one day, what should you do?	12 ITBhivms — *Take one and half doses*
5	In the past 3 months, have you set personal goals for your HIV treatment?	13 ITBhivgl *No*
6	In the past 3 months, have you asked questions to your medical provider about your HIV?	14 ITBhivq *No*

IndexBeliefQues [1]

		Agree strongly / Agree / Disagree / Disagree strongly
7	If you have side effects, you should continue to take your substance use treatment.	15 ITBsuse
8	Substitution therapy (site specific, only ask if available) therapy is safe.	16 ITBsubsf *N/A*
9	Substance use treatment is effective (by effective we mean, does it work).	17 ITBsuef
10	If you miss your substance use treatment medication one day, what should you do?	18 ITBsums — *Take one and half doses*

Version 19 20 VersDt | 21 formlang | Completed by: 22 InitDate *(initials/date)*

VersionInitDate [1]

(HPTN 074) DF/Net 074 (UDS) 165

Visit Code: 6 visit

Participant ID: 7 ptid

Site Number | Participant Number | Chk | Cohort

Specimen Collection Date: 8 Date

dd MMM yy

Header [1]

Urine Drug Screen

rineDrugScreen [1

9 UDSnd Not done/Not collected → **End of form.**

1 Was drug detected?

		Yes	No	Not done/ Not collected
1a.	Amphetamines	10 UDSamp		
1b.	Methamphetamines	11 UDSmamp		
1c.	Methadone	12 UDSmdn		
1d.	Opiates/morphine	13 UDSop		
1e.	Benzodiazepines	14 UDSbenz		
1f.	Buprenorphine	15 UDSbup		
1g.	Cannabinoids	16 UDScann		
1h.	Cocaine metabolite	17 UDSco		
1i.	Other, specify:	18 UDSoth		

(Local language) ______________________

(English) 19 UDSox

Comments *(Local language)*: ______________________

Comments *(English)*: 20 UDScomm

/ersionInitDate [1

Version 1 21 22 VersDt | 23 formlang | Completed by: 24 InitDate *(initials/date)*

Participant ID: 7 ptid

Site Number | Network Number | Chk | Cohort

Assessment Date: 8 Date

dd MMM yy

Header [1]

Follow-up Behavior Assessment

A broad range of participants are taking part in this study. So some questions may not apply to you. We ask the same questions of all participants.

In this interview, you will be asked about your sexual behaviors and possible alcohol and drug use. Some of the questions may seem very personal. Please remember that all of your answers will be kept private. Your responses are linked only to your study number, not to your name.

We need you to answer these questions honestly. *We do not judge about alcohol or drug use. We make no judgments about how you have sex or the number of times or with whom.*

These questions help us understand the type of behaviors our participants are doing while on study. Your honesty will help us understand these differences in behaviors.

FOLLOW-UP DEMOGRAPHICS

1 How would you describe your current living situation? *Mark all that apply.*

FuBehavAssess [1

- ☐ 9 FBAlshou *1a. Live in a house that I own or rent*
- ☐ 10 FBAlspar *1b. Live in parent's home*
- ☐ 11 FBAlsrel *1c. Live in other relative's home (e.g, sibling, cousin, aunt and/or uncle)*
- ☐ 12 FBAlsrnt *1d. Rent a room or space in someone else's house or apartment*
- ☐ 13 FBAlsfre *1e. Stay with someone else for free (don't pay regular room and board)*
- ☐ 14 FBAlsst *1f. Live on the street, in a park, alley or abandoned building*
- ☐ 15 FBAlsshe *1g. Live in a homeless shelter*
- ☐ 16 FBAlsdif *1h. Stay at more than two different places a week*
- ☐ 17 FBAlstrn *1i. Live in a drug recovery or any transitional house*
- ☐ 18 FBAlso *1j. Other place, specify:*

Local language: ______________________

English: 19 FBAlsox

- ☐ 20 FBAlsref *1k. Refuse to answer*

VersionInitDate [1

Follow-up Behavior Assessment

ehavAssess [1

2 Who lives with you? *Mark all that apply.*

- 8 FBAlvspo *2a. Spouse/partner/girl-boyfriend*
- 9 FBAlvch *2b. Own children*
- 10 FBAlvpar *2c. Parents*
- 11 FBAlvfam *2d. Other family members*
- 12 FBAlvfr *2e. Friends*
- 13 FBAlvoth *2f. Others (e.g., roommates, tenants)*
- 14 FBAlvalo *2g. Live alone*
- 15 FBAlvref *2h. Refuse to answer*

3 What is your relationship status? Are you:

16 FBAmar

- *Married*
- *Living with a sexual partner but not married*
- *Separated*
- *Divorced*
- *Widowed*
- *Single*
- *Refuse to answer*

4 At any time during the last 3 months, that is from today, have you been unemployed?

17 FBAun3mo

- *Yes*
- *No*
- *Refuse to answer*

(HPTN 074) DF/Net 074 (FBA-3) 173

Visit Code: 6 visit

Participant ID: 7 ptid

Site Number | Network Number | Chk | Cohort

Header [1]

Follow-up Behavior Assessment

5 Which of the following best describes your current employment status? Are you:

FuBehavAssess [1

8 FBAemp
- *Employed full-time*
- *Employed part-time*
- *Unemployed but seeking work*
- *Unemployed—not seeking work*
- *Retired*
- *Refuse to answer*

6 In the past month, how many days have you been working for pay? 9 FBAwrkdy *# days*

7 ***(Index: Intervention ONLY)*** Do you have a designated support person? 10 FBAdsgns *No* → ***If no, go to item 8.***

7a. What is the relationship of the support person to you?

11 FBAdrel
- *Spouse/partner/girl-boyfriend*
- *Father/mother*
- *Sibling*
- *Other family member*
- *Friend*
- *Other, specify:* *Local language:* ______ *English:* 12 FBAdrelx

INCARCERATION

8 In **the last 3 months**, how many different times were you in...

8a. Jail/prison? 13 FBAjail *# of times*

8b. Involuntary detoxification/detention? 14 FBAdetox *# of times*

8c. Other incarceration, specify: 15 FBAoinc *# of times*

Local language: ______ *English:* 16 FBAoincx

VersionInitDate [1

Version 3 17 18 VersDt | 19 formlang | Completed by: 20 InitDate *(initials/date)*

(HPTN 074) DF/Net 074 (FBA-4) 174

Visit Code: 6 visit

Participant ID: 7 ptid

Site Number | Network Number | Chk | Cohort

Header [1]

Follow-up Behavior Assessment

ALCOHOL USE (Audit C) *Answer questions to reflect your behavior over the past 3 months.*

FuBehavAssess [1

9 How often do you have a drink containing alcohol?

8 FBAafreq

2–3 times a week

4 or more times a week

10 How many standard drinks containing alcohol do you have on a typical day?

9 FBAanum

7 to 9

10 or more

11 How often do you have six or more drinks on one occasion?

10 FBA6drnk

Weekly

Daily or almost daily

NON-INJECTION DRUG USE

12 In the **last 3 months**, did you... *Yes* *No*

12a. Use marijuana? 11 FBAnimj

12b. Use stimulants (cocaine, methamphetamines)? 12 FBAnist

12c. Use opiates (heroin, opium)? 13 FBAniop

12d. Use other drugs *(as indicated by sites)*, specify: 14 FBAnioth

Local language: ____________________

English: 15 FBAniox

13 When was the last time you used marijuana, amphetamines, methadone, opiates or benzodiazepines that you did not inject? 16 FBAnidt (*dd* *MMM* *yy*) OR *Never* 17 FBAninev

VersionInitDate [1

Version 18 19 VersDt | 20 formlang | Completed by: 21 InitDate *(initials/date)*

Participant ID:
7 ptid
Site Number | Network Number | Chk | Cohort

Header [1]

Follow-up Behavior Assessment

INJECTION DRUG USE

FuBehavAssess [1

14 In **the last 3 months**, did you use a needle to inject any drugs under your skin or into a vein? 8 FBAinj3m No → ***If no, go to item 33 on page 8.***

15 In the **last 3 months**, which of the following did you inject?

	Yes / No		Yes / No
15a. Amphetamines	9 FBAiamp	15i. Ketamine	17 FBAiket
15b. Methamphetamines	10 FBAimamp	15j. Homemade opiods	18 FBAihop
15c. Cocaine	11 FBAicoc	15k. Short and long action stimulants	19 FBAisas
15d. Heroin	12 FBAiher	15l. Dezomorphine	20 FBAidez
15e. Opium	13 FBAiop	15m. Other, specify:	21 FBAioth
15f. Buprenorphine	14 FBAibup	*Local language:* ______	
15g. Methadone	15 FBAimeth	*English:* 22 FBAiotx	
15h. Benzodiazepines	16 FBAibenz		

16 In **the last month**, on how many days did you inject drugs? 23 FBAinjd *# of days*

17 In **the last month**, on days that you injected, how many times a day did you usually inject drugs? 24 FBAinit *# of times a day*

18 When was the last day you injected (including today)? 25 FBAidt *dd MMM yy* OR 26 FBAima *# of months ago*

VersionInitDate [1

Version 3 27 28 VersDt | 29 formlang | **Completed by:** 30 InitDate *(initials/date)*

Participant ID:
7 ptid
Site Number | Network Number | Chk | Cohort

Header [1]

Follow-up Behavior Assessment

SHARING

Often people share syringes and other equipment because they are in a rush, they are concerned about the police, feeling sick or for a variety of other reasons.

		Yes / *No* / *Don't know*
19	Have you shared rinse water in **the last 3 months**?	9 FBAsrw — *If no, go to item 20.*
	19a. The last time you injected, did you share rinse water?	10 FBAsrwls
20	Have you shared a cooker/container in **the last 3 months**?	11 FBAscc — *If no, go to item 21.*
	20a. The last time you injected, did you share a cooker/container?	12 FBAsccls
21	Have you shared filter cotton in **the last 3 months**?	13 FBAsfc — *If no, go to item 22.*
	21a. The last time you injected, did you share filter cotton?	14 FBAsfcls

CLEANING

22 How often do you clean your needle before injecting?

15 FBAclnf *Never*

	Yes / *No* / *Don't know*
22a. The last time you injected, did you clean the needle before you injected?	16 FBAndlc
22b. The last time you injected, did you use a new needle?	17 FBAndln

22c. How often do you clean your needle after injecting?

18 FBAclna *Never*

	Yes / *No* / *Don't know*
22d. The last time you injected, did you clean the needle after you injected?	19 FBAclnls

VersionInitDate [1

Version 20 21 VersDt | 22 formlang | Completed by: 23 InitDate *(initials/date)*

Participant ID:
7 ptid
Site Number Network Number Chk Cohort
Header [1]

Follow-up Behavior Assessment

PRE-FILLING AND BACKLOADING SYRINGES

23	The last time you injected, did you use a pre-filled syringe?	9 FBApfs *Don't know*	
24	The last time you injected, did you inject drugs that were frontloaded or backloaded into the syringe or needle that you used?	10 FBAfbs *Don't know*	
25	In **the last 3 months**, how many different people did you use injection drugs with?	11 FBApeop *# of people*	→ ***If 000, go to item 31.***
26	In **the last 3 months**, how many times did you even once pass on a needle or syringe to someone else after you used it?	12 FBApass *# of times*	→ ***If 000, go to item 28.***
27	In **the last 3 months**, with how many different people did you pass on a needle or syringe after you had used it?	13 FBApassn *# of people*	
28	In **the last 3 months**, how many times did you ever, even once, use a needle or syringe after someone else used it?	14 FBAused *# of times*	→ ***If 000, go to item 30.***
29	In **the last 3 months**, with how many different people did you use a needle or syringe after someone else used it?	15 FBAusedn *# of people*	

30 In **the past 3 months**, when you shared syringes, how often did you inject last?

16 FBAilast
Sometimes
I have not shared in the past 3 months

31 In **the last 3 months**, did you get your new needles/syringes from a...

31a. 17 FBAnonew *I did not get new needles in the last 3 months* → ***Go to item 33 on page 8.***

		Yes / *No*
31b.	Pharmacy or health center?	18 FBApharm
31c.	Needle seller?	19 FBAsellr
31d.	Needle exchange?	20 FBAnexch
31e.	Other drug injectors?	21 FBAodinj
31f.	Other, specify?	22 FBAosrc

Local language: ______________________ *English:* 23 FBAosrcx

32 In **the last 3 months**, when you obtained new needles, typically, how many did you buy at one time? 24 FBAbuy *# of needles*

VersionInitDate [1

Version 3 25 26 VersDt 27 formlang Completed by: 28 InitDate *(initials/date)*

Follow-up Behavior Assessment

SEXUAL BEHAVIOR QUESTIONS

The next few questions are about sexual behavior. These are very personal questions. Please remember that all the information you give us is confidential. We ask that you answer the questions as honestly as you can.

FuBehavAssess [1]

33	In **the last month**, did you have vaginal or anal sex?	8 FBAsexlm *No*	→ *If no, go to item 39.*
34	In **the last month**, how many different **female** sex partners have you had?	9 FBAsexf *# of female sex partners*	
35	In **the last month**, how many different **male** sex partners have you had?	10 FBAsexm *# of male sex partners*	
36	Do you have a main partner such as a husband/wife or boyfriend/girlfriend?	11 FBAmp *No*	→ *If no, go to item 38.*
	36a. Is your main partner male or female? 12 FBAmpgen *Other, specify:* *(Local language)* ______ *(English)* 13 FBAmpgnx		
37	In **the last month**, how many times did you have vaginal or anal sex with your main partner?	14 FBAsexmp *# of times*	→ *If 000, go to item 38.*
	37a. How many of these times did you (or your partner) use a condom?	15 FBAmpcon *# of times*	
38	In **the last month**, how many times did you have vaginal or anal sex with someone other than a main partner?	16 FBAsexop *# of times*	→ *If 000, go to item 39.*
	38a. How many of these times did you (or your partner) use a condom?	17 FBAopcon *# of times*	

Next I am going to ask about your sex trade (paid or paying) partners. By sex trade partners, I mean people that you had oral, vaginal or anal sex with in exchange for money, drugs, food or a place to stay.

39	In **the last month**, how many times did you give sex partners money or drugs in exchange for sex?	18 FBAexchy *# of times*	
40	In **the last month**, how many times did a sex partner give you money or drugs in exchange for sex?	19 FBAexchp *# of times*	

VersionInitDate [1]

(HPTN 074) DF/Net 074 (IFH-1) 185

Visit Code: 6 visit

Participant ID: 7 ptid

Site Number | Network Number | Chk | Cohort

Assessment Date: 8 Date

dd MMM yy

Header [1]

Index Follow-up HIV Care and ART

The next few questions are about anti-HIV medicines called antiretroviral therapy that you may be taking. These questions are not about drugs you may be taking to treat substance use. We need to know what is really happening, not what you think we "want to hear." Many people find it hard to take their medications all of the time.

IndexFllwUpHIV [1]

1 Since your last visit, have you seen an HIV clinician? 9 IFHcln *No* → ***If no, go to item 2.***

1a. If yes, on what date? 10 IFHclndt
dd MMM yy

1b. How many total clinical visits have you had since your last study visit? 11 IFHclnnm *# of clinical visits*

2 Since your last study visit, with whom have you shared that you are living with HIV? *Mark all that apply.*

- 12 IFHspous *2a. Spouse/Partner/Girl-Boyfriend*
- 13 IFHchild *2b. Own children*
- 14 IFHparen *2c. Parents*
- 15 IFHofm *2d. Other family members*
- 16 IFHfrnds *2e. Friends*
- 17 IFHshoth *2f. Others*
- 18 IFHnoone *2g. No one*
- 19 IFHref *2h. Refuse to answer*

3 How many of your drug partners (the people you inject drugs with) know that you have HIV?

20 IFHptnr

Less than half of my partners

None of my partners

VersionInitDate [1]

Version 21 22 VersDt | 23 formlang | Completed by: 24 InitDate *(initials/date)*

Index Follow-up HIV Care and ART

4 Who is the most supportive in helping you with your HIV care and treatment?

ndexFllwUpHIV [1

8 IFHsuprt

- *Health care providers (such as doctors or nurses)*
- *Your spouse/live-in partner/long-term partner*
- *Injecting partners*
- *Commercial sex workers*
- *Your mother or father*
- *Your in-laws*
- *Other family members*
- *Priests/religious leaders*
- *Your neighbors or community members*
- *Friends*
- *Your employers*
- *Other, specify:*

Local language: ______ *English:* 9 IFHothx

5 How often have you talked to your family or support person about your HIV medications?

10 IFHtalk

- *Once a month*
- *One time*
- *Never*

6 Since your last visit, has any HIV clinician told you that you should initiate ART treatment for your own health?

11 IFHainit

- *Yes*
- *No*
- *Not applicable*

7 Since your last visit, have you visited an HIV clinic or pharmacy with the intention of starting ART?

12 IFHastrt *No*

(HPTN 074) DF/Net 074 (IFH-3) 187

Visit Code: 6 visit

Participant ID: 7 ptid

Site Number | Network Number | Chk | Cohort

Header [1]

Index Follow-up HIV Care and ART

ndexFllwUpHIV [1

8 Are you currently taking HIV medications (ART)? 8 IFHacurr No → *If no, go to item 16 on page 4.*

8a. Since what date have you been on HIV medications (ART) treatment? 9 IFHartdt (dd MMM yy) OR 10 IFHck Since last study visit

9 Since your last study visit, with whom have you shared that you are taking ART? *Mark all that apply.*

- 11 IFHaspou 9a. Spouse/Partner/Girl-Boyfriend
- 12 IFHachld 9b. Own children
- 13 IFHaprnt 9c. Parents
- 14 IFHaofm 9d. Other family members
- 15 IFHafrnd 9e. Friends
- 16 IFHaoth 9f. Others
- 17 IFHanone 9g. No one
- 18 IFHaref 9h. Refuse to answer
- 19 IFHant 9i. I've not taken ART since my last study visit

10 Since your last study visit, has your doctor changed your HIV medications (ART) drugs? 20 IFHachng No → *If no, go to item 11.*

10a. Were your HIV medications (ART) drugs changed due to side effects or some other reason? 21 IFHacrsn something else

11 We know that it is very difficult to take your HIV medications all the time. When was the last time you missed taking any doses of your ART medication?

22 IFHalast

Never missed taking medications in past 3 months → **End of form.**

12 In the **last month**, on about how many days did you miss at least one tablet? 23 IFHadays days

13 Did you miss at least one tablet in **the last four days**? 24 IFHa4day No → *If no, go to item 15 on page 4.*

13a. In **the last four days**, on how many days did you miss at least one tablet? 25 IFHa4num days

14 Did you miss at least one tablet **yesterday**? 26 IFHayest No

/ersionInitDate [1

Version 2. 27 1- 28 VersDt 29 formlang Completed by: 30 InitDate (initials/date)

Visit Code: 6 visit

ndexFllwUpHIV [1

No data recorded on this page 8 IFH4nd

Participant ID:

7 ptid

Site Number Network Number Chk Cohort

Header [1]

Index Follow-up HIV Care and ART

15 The last time you missed at least one tablet, which of these best describes the reason why? ***Mark only one.***

9 IFHarsn

- *I forgot*
- *I did not have the medication with me at the time I needed to take it*
- *I did not want person(s) nearby to see me taking the medication*
- *I was trying to avoid side effects*
- *I felt healthy*
- *I was drunk*
- *I am not engaging in any behaviors that can transmit HIV to others*
- *I do not believe the medicines are beneficial*
- *not applicable—no missed tablets*
- *Other, specify:*

Local language: ________________ *English:* 10 IFHarsx

16 If you were on ART since your last visit, have you stopped? By stopped we mean that you have not taken any of your medication for the last 2 weeks. 11 IFHastop *No* → ***If no, go to item 18.***

17 What date did you stop taking ART? 12 IFHaspdt

dd MMM yy

VersionInitDate [1

Version 2 13 1-14 VersDt 15 formlang **Completed by:** 16 InitDate *(initials/date)*

(HPTN 074) DF/Net 074 (IFH-5) 189

Visit Code: 6 visit

ndexFllwUpHIV [1

No data recorded on this page 8 IFH4nd

Participant ID: 7 ptid

Site Number | Network Number | Chk | Cohort

Header [1]

Index Follow-up HIV Care and ART

		Agree	Disagree	Not relevant/ Not Applicable
18	Sometimes people have trouble taking HIV medications (ART) because of personal situations or circumstances. What are the reasons you have stopped taking ART or have not yet started to take ART?			
	18a. You don't think that you need it.	9 IFHpneed		
	18b. You don't have time to go to the clinic.	10 IFHptime		
	18c. You don't have the energy or motivation to go to the clinic.	11 IFHpenrg		
	18d. You missed an appointment, so it is difficult to go back.	12 IFHpappt		
	18e. You have been too sick to go to the clinic.	13 IFHpsick		
	18f. Your drug use got in the way of going to the clinic.	14 IFHpdrug		
	18g. You had unwanted side effects or complications.	15 IFHpsaff		
	18h. You don't understand when you are supposed to take each pill.	16 IFHpunds		
	18i. You sold or lost your medicine or your medicine was stolen.	17 IFHplost		
	18j. Your drinking (alcohol) got in the way of going back to the clinic.	18 IFHpalc		
	18k. You had to wait too long, so you left without being seen.	19 IFHpwait		
19	Sometimes people find it difficult to take HIV medications (ART) because of their family or friends. What are the reasons you have stopped taking ART or have not yet started to take ART?			
	19a. You don't have enough support from family or friends.	20 IFHfnosp		
	19b. You don't want others to know you have HIV (e.g., see me take medication).	21 IFHfknow		

Version [illegible] 22 V 23 VersDt | 24 formlang | /ersionInitDate [1 | Completed by: 25 InitDate (initials/date)

Participant ID:
7 ptid

Site Number Network Number Chk Cohort

Header [1]

Index Follow-up HIV Care and ART

		Agree	Disagree	Not relevant/ Not applicable
20	Many people find it difficult to take HIV medications (ART), even if they have previously taken ART, because of challenges in the health systems/health care process. What were the reasons you have stopped taking ART or have not yet started to take ART?			
20a.	You do not have the necessary materials (e.g. paperwork, identification, test results) needed by the clinic.	9 IFHhmat		
20b.	ART is too expensive.	10 IFHhexp		
20c.	ART was not available.	11 IFHhna		
20d.	HIV care provider would not continue your medicine or prescription.	12 IFHhmed		
20e.	You do not know how or where to get ART.	13 IFHhget		
20f.	You find transport/travel to the clinic is difficult.	14 IFHhtran		
20g.	You have been traveling.	15 IFHhtrav		
20h.	You have been in jail or incarcerated.	16 IFHhjail		
20i.	You are scared of being arrested for your drug use.	17 IFHharst		
20j.	You will need to register with the government as being a drug user.	18 IFHhgov		

21 Are there any other reasons you find it difficult to continue taking ART or why you have not yet started to take ART?

Local language: ______________________________

English: 19 IFHartx

Version 20 21 VersDt 22 formlang VersionInitDate [1 Completed by: 23 InitDate *(initials/date)*

Visit Code: 6 visit

Participant ID: 7 ptid

Site Number | Network Number | Chk | Cohort

Assessment Date: 8 Date

dd MMM yy

Header [1]

Follow-up Substance Use Treatment

uSubstanceUse [1

1	Since your last visit, have you tried to start MAT (methadone or buprenorphine) to treat your substance use?	9 FSUstart No → *If no, go to item 2.*
	1a. When was your last visit?	10 FSUlvdt (dd MMM yy)
2	Are you currently taking methadone or buprenorphine?	11 FSUcrrnt No → *If no, go to item 3.* → *If yes, and this is the participant's first time in MAT during the study, complete the Substance Use Treatment Initiation CRF.*
	2a. How long have you currently been on MAT (methadone or buprenorphine)?	12 FSUmnths months
3	If you were on substance use treatment since your last visit, have you stopped taking MAT or buprenorphine? By stopped, we mean that you have not been taking it for at least 2 weeks.	13 FSUstop No → *If no, end of form.*
	3a. What date did you stop taking MMT or buprenorphine?	14 FSUstpdt (dd MMM yy)

VersionInitDate [1

Version 2. 15 5- 16 VersDt | 17 formlang | Completed by: 18 InitDate (initials/date)

(HPTN 074) DF/Net 074 (FSU-2) 202

Visit Code: 6 visit
uSubstanceUse [1
No data recorded on this page 8 FSUndr

Participant ID:
7 ptid
Site Number Network Number Chk Cohort

Header [1]

Follow-up Substance Use Treatment

4 Sometimes people find it difficult to continue MMT or buprenorphine because of personal situations or circumstances. What are the reasons you have stopped MMT or buprenorphine?

		Agree	Disagree	Not relevant/ Not applicable
4a.	You want to keep injecting.	9 FSUkpinj		
4b.	You want to handle your drug problem or stop injecting on your own.	10 FSUwnt2h		
4c.	You had side effects or withdrawal symptoms.	11 FSUsidef		
4d.	Your injecting got in the way of going to the clinic.	12 FSUinjnw		
4e.	Your drug use got in the way of going to the clinic.	13 FSUdrunw		
4f.	You have been too sick to go to the clinic.	14 FSU2sick		
4g.	You missed an appointment, so it is difficult to go back.	15 FSUmissa		
4h.	You can handle your drug problem yourself.	16 FSUcnhnd		
4i.	Your drinking (alcohol) got in the way of going to the clinic.	17 FSUdrink		

5 Sometimes people find it difficult to continue MMT or buprenorphine because of their family or friends. What are the reasons you have stopped MMT or buprenorphine?

		Agree	Disagree	Not relevant/ Not applicable
5a.	You don't have enough support from family or friends.	18 FSUnosup		
5b.	You don't want to stop seeing the friends you inject with.	19 FSUfrinj		
5c.	Your family objects to drug treatment.	20 FSUfamob		
5d.	You don't feel you have enough support from friends and family.	21 FSUflnos		

VersionInitDate [1

Version 22 25 23 VersDt 24 formlang Completed by: 25 InitDate (initials/date)

Visit Code: 6 visit

uSubstanceUse [1

No data recorded on this page 8 FSUndr

Participant ID:

7 ptid

Site Number | Network Number | Chk | Cohort

Header [1]

Follow-up Substance Use Treatment

6 Many people find it difficult to continue MMT or buprenorphine, even if they have previously tried, because of challenges in the health systems/health care process. What were the reasons you have not continued MMT or buprenorphine?

	Agree / Disagree / Not relevant/ Not applicable
6a. The waiting list is too long.	9 FSUwaitl
6b. You don't have the necessary materials (ID card, paperwork).	10 FSUneedm
6c. You find getting on drug treatment is too much of a hassle.	11 FSUhassl
6d. You do not know how or where to get drug treatment.	12 FSUnoknw
6e. You find transport/travel to the clinic is difficult.	13 FSUtrnsd
6f. You have been traveling.	14 FSUtravl
6g. You have been in jail or incarcerated.	15 FSUjncar
6h. You are scared of being arrested for your drug use.	16 FSUscrar
6i. You are scared of losing your job.	17 FSUscrjb
6j. You were asked to leave MAT.	18 FSUleave

7 Are there any other reasons you find it difficult to continue MAT or buprenorphine?

Local language: ______

English: 19 FSUothx

VersionInitDate [1

Version 2 20 21 VersDt | 22 formlang | Completed by: 23 InitDate *(initials/date)*

Participant ID: 7 ptid

Site Number | Network Number | Chk | Cohort

Assessment Date: 8 Date

dd MMM yy

Header [1]

Systems Navigator Encounter

ystemNavigator [1

1	Staff ID: 9 SNEstfid
2	Activity type: 10 SNEatype *Interim*
3	Contact initiated/requested by: 11 SNEcreq *Participant* *Other, specify:* *Local language:* ____ *English:* 12 SNEcreqx
4	Type of encounter: 13 SNEncntr *E-mail/Internet* *Text/SMS* *Other, specify:* *Local language:* ____ *English:* 14 SNEncntx
5	Is the participant currently taking antiretroviral therapy (ART)? 15 SNEctart *No*
6	Is the participant currently receiving methadone or buprenorphine? 16 SNEcrmth *No*
7	Does the participant have a designated support person? 17 SNEdsgns *No* → ***If no, go to item 8 on page 2.***
	7a. Did the support person participate in this contact activity? 18 SNEdspar *No*
	7b. What is the relationship of the support person to the participant? 19 SNEdrel *Friend* *Other, specify:* *Local language:* ____ *English:* 20 SNEdrelx

/ersionInitDate [1

Version 1 21 22 VersDt | 23 formlang | Completed by: 24 InitDate *(initials/date)*

Systems Navigator Encounter

ystemNavigator [1

8 Total number of minutes of activity: *0–5 minutes* *6–10 minutes* *11–15 minutes* *16–30* [illegible] *utes*

8 SNEnmins

9 Primary focus of contact:

9 SNEprifc

Legal/Judicial
Sexual health services
Social services
Priests/religious leaders
Your neighbors or community members
Clinical/medical management
Other, specify:

Local language: ____________

English: 10 SNEprifx

10 Additional areas addressed: ***Mark all that apply.***

- 11 SNEsubut *10a. Substance use treatment*
- 12 SNEhivc *10b. HIV care*
- 13 SNEexpgm *10c. Needle and syringe exchange programs*
- 14 SNEartim *10d. ART initiation/Management*
- 15 SNEapdsp *10e. Assistance with primary designated support person*
- 16 SNEsnetw *10f. Social network management*
- 17 SNEacnsl *10g. Additional counseling*
- 18 SNElegal *10h. Legal/Judicial*
- 19 SNEsexhs *10i. Sexual health services*
- 20 SNEsocsv *10j. Social services*
- 21 SNErelig *10k. Priests/religious leaders*
- 22 SNEneigh *10l. Your neighbors or community members*
- 23 SNEclnmd *10m. Clinical/medical management*
- 24 SNEoth *10n. Other, specify:*

Local language: ____________

English: 25 SNEothx

VersionInitDate [1

Version 1.[illegible] 26 27 VersDt | 28 formlang | Completed by: 29 InitDate *(initials/date)*

(HPTN 074) DF/Net 074 (SNE-3) 212

Encounter #: 6 visit

Note: Number encounters sequentially (001, 002, 003) for each participant.

Participant ID: 7 ptid

Site Number | Network Number | Chk | Cohort

Header [1]

Systems Navigator Encounter

ystemNavigator [1

11 Assessment of client status:

8 SNEcstat

- *Client has no immediate complicating psychosocial issues*
- *Client has complicating psychosocial issues that post minimal risk to ART or substance use adherence*
- *Client has complicating psychosocial issues that pose moderate risk to ART or substance use adherence*
- *Client has complicating psychosocial issues that pose severe risk to ART or substance use adherence*

12 Was a referral made at this encounter? 9 SNErefer *No* → ***If no, go to item 13.***

12a. What was the referral made for? ***Mark all that apply.***

- 10 SNErhiv *HIV care (ART)*
- 11 SNErisut *Initial substance use treatment*
- 12 SNErrsut *Reentry into substance use treatment*
- 13 SNErepgm *Needle/syringe exchange program*
- 14 SNErmntl *Mental health counseling*

13 Was a social impact reported at this encounter? 15 SNEsil *No* → ***If yes, complete Social Impact Log for each impact. If this is an interim visit, also complete a Follow-up Visit form.***

14 Was a Serious Adverse Experience reported at this encounter? 16 SNEae *No* → ***If yes, complete AE Log for each reported AE. If this is an interim visit, also complete a Follow-up Visit form.***

15 Did the participant report initiating any antiretroviral medications for treatment at this encounter? 17 SNEiai *No* → ***If yes, complete Index ART Initiation CRF. If this is an interim visit, also complete a Follow-up Visit form.***

16 Did the participant report initiating substance use treatment (MAT) at this encounter? 18 SNEsut *No* → ***If yes, complete Substance Use Treatment Initiation CRF. If this is an interim visit, also complete a Follow-up Visit form.***

VersionInitDate [1

Version 19 20 VersDt | 21 formlang | Completed by: 22 InitDate *(initials/date)*

(HPTN 074) DF/Net 074 (PSY-1) 225

Encounter #: 6 visit

Note: Number encounters sequentially (001, 002, 003) for each participant.

Participant ID: 7 ptid

Site Number | Network Number | Chk | Cohort

Assessment Date: 8 Date

dd MMM yy

Header [1]

Psychosocial Encounter

PsychEncounter [1]

1 Counselor ID: 9 PSYstfid

2 Contact type: 10 PSYctype

- *Study site visit*
- *Telephone contact*
- *Off-site contact, specify:*

Local language: ____________

English: 11 PSYcothx

3 Activity type: 12 PSYatype

- *Introductory session*
- *First session*
- *Second session*
- *Booster session*
- *Index and supporter session*

4 Does the participant have a designated support person? 13 PSYdsgns *No* → ***If no, go to item 5 on page 2.***

4a. Did the support person participate in this contact activity? 14 PSYdspar *No*

4b. What is the relationship of the support person to the participant? 15 PSYdrel

- *Friend*
- Other, specify:

Local language: ____________ *English:* 16 PSYdrelx

VersionInitDate [1]

Version 17 18 VersDt | 19 formlang | Completed by: 20 InitDate *(initials/date)*

(HPTN 074) DF/Net 074 (PSY-2) 226

Encounter #: 6 visit

Note: Number encounters sequentially (001, 002, 003) for each participant.

Participant ID: 7 ptid

Site Number | Network Number | Chk | Cohort

Header [1]

Psychosocial Encounter

5 Indicate focus of session. *Mark all that apply.*

PsychEncounter [1

- [ ] 8 PSYhinf 5a. *Dealing with HIV infection*
- [ ] 9 PSYfhivd 5b. *HIV disclosure*
- [ ] 10 PSYfrr 5c. *Risk reduction*
- [ ] 11 PSYfmap 5d. *ART adherence and/or development of medication adherence plan*
- [ ] 12 PSYfeng 5e. *Engagement into care*
- [ ] 13 PSYfacs 5f. *Adherence communication skills*
- [ ] 14 PSYfsrcs 5g. *Sexual risk communication skills*
- [ ] 15 PSYfircs 5h. *Injection risk communication skills*
- [ ] 16 PSYfrss 5i. *Relationships/Social support*
- [ ] 17 PSYflit 5j. *HIV literacy*
- [ ] 18 PSYfoth 5k. *Other, specify:* *Local language:* ______ *English:* 19 PSYfothx

VersionInitDate [1

Version 2 20 21 VersDt 25 | 22 formlang | **Completed by:** 23 InitDate ______ *(initials/date)*

Encounter #: 6 visit

Note: Number encounters sequentially (001, 002, 003) for each participant.

Participant ID: 7 ptid

Site Number Network Number Chk Cohort

Header [1]

Psychosocial Encounter

6 Barriers to ART addressed. *Mark all that apply.*

PsychEncounter [1

- 8 PSYbart *6a. Need for ART*
- 9 PSYbhass *6b. Hassle of taking medication*
- 10 PSYbtime *6c. Time to go to the clinic*
- 11 PSYbgtc *6d. Challenges getting to the clinic*
- 12 PSYbinc *6e. Challenges/Issues in the clinic*
- 13 PSYbmiss *6f. Previously missed appointments*
- 14 PSYbsick *6g. Too sick*
- 15 PSYbalc *6h. Alcohol use interference*
- 16 PSYbdrug *6i. Drug use interference*
- 17 PSYbfood *6j. Don't have food/water to take medicine*
- 18 PSYbmeds *6k. Don't understand when/how to take medicine*
- 19 PSYbse *6l. Side effects/complications*
- 20 PSYbsupp *6m. Family or friend support*
- 21 PSYbdisc *6n. Disclosure issues, including fear of inadvertent disclosure, strategies for intentional disclosure*
- 22 PSYbnav *6o. Navigating structural issues–referral to systems navigator*
- 23 PSYboth *6p. Other, specify:* *Local language:* ____ *English:* 24 PSYbothx

VersionInitDate [1

Completed by: 28 InitDate *(initials/date)*

(HPTN 074) DF/Net 074 (PSY-4) 228

Encounter #: 6 visit

Note: Number encounters sequentially (001, 002, 003) for each participant.

Participant ID: 7 ptid

Site Number | Network Number | Chk | Cohort

Header [1]

Psychosocial Encounter

7 *Barriers to MMT/buprenorphine addressed. Mark all that apply.*

PsychEncounter [1

- ☐ 8 PSYmmmt *7a. Need for MMT/buprenorphine*
- ☐ 9 PSYmhass *7b. Hassle of taking medication*
- ☐ 10 PSYmtime *7c. Time to go to the clinic*
- ☐ 11 PSYmgtc *7d. Going to the clinic is a hassle*
- ☐ 12 PSYmmiss *7e. Previously missed appointments*
- ☐ 13 PSYmsick *7f. Too sick*
- ☐ 14 PSYmalc *7g. Alcohol use interference*
- ☐ 15 PSYmdrug *7h. Drug use interference*
- ☐ 16 PSYmfood *7i. Don't have food/water to take medicine*
- ☐ 17 PSYmmeds *7j. Don't understand when/how to take medicine*
- ☐ 18 PSYmse *7k. Side effects/complications*
- ☐ 19 PSYmsupp *7l. Family or friend support*
- ☐ 20 PSYmnav *7m. Navigating structural issues–referral to systems navigator*
- ☐ 21 PSYmoth *7n. Other, specify:*

Local language: ____________________

English: 22 PSYmothx

8 Total number of minutes of activity:

0–5 minutes | *6–10 minutes* | *11–15 minutes*

16–30 [illegible] *utes*

23 PSYmin

9 As a result of this counseling, were systems navigator services provided? 24 PSYsnsp *No*

VersionInitDate [1

Version 2 25 5- 26 VersDt | 27 formlang | Completed by: 28 InitDate *(initials/date)*

(HPTN 074) DF/Net 074 (SS) 230

Visit Code: 6 visit

Participant ID: 7 ptid

Site Number | Network Number | Chk | Cohort

Header [1]

Specimen Storage

SpecimenStorage [1

1 PLASMA

Specimen Collection Date: 8 SSplsdt (dd MMM yy)

9 SSpls — Not required

1a. Reason not stored or not collected: Local language: ______ English: 10 SSplsr

2 FROZEN URINE

Specimen Collection Date: 11 SSfurndt (dd MMM yy)

12 SSfurn — Not required

2a. Reason not stored or not collected: Local language: ______ English: 13 SSfurnr

3 DRIED URINE CARTRIDGE

Specimen Collection Date: 14 SSdurndt (dd MMM yy)

15 SSdurn — Not required

3a. Reason not stored or not collected: Local language: ______ English: 16 SSdurnr

4 DRIED URINE FILTER PAPER

Specimen Collection Date: 17 SSufpdt (dd MMM yy)

18 SSufp — Not required

4a. Reason not stored or not collected: Local language: ______ English: 19 SSufpr

Comments (*Local language*): ______

Comments (*English*): 20 SScomm

VersionInitDate [1

Version 1 21 22 VersDt 23 formlang Completed by: 24 InitDate (initials/date)

(HPTN 074) DF/Net 074 (ACL) 232

Page: 6 visit

Participant ID: 7 ptid

Site Number | Network Number | Chk | Cohort

Date of Contact: 8 Date

dd MMM yy

Header [1]

Agency Contact

AgencyContact [1]

1 Contact initiated by:

9 ACLinit

- *Systems navigator*
- *Counselor*
- *Agency/clinic*
- *Other, specify:* *(Local Language)* ________ *(English)* 10 ACLcothx

2 Type of facility contacted:

11 ACLfacil

- *HIV clinic*
- *Substance use treatment facility*
- *Needle and syringe exchange program*
- *Mental health counselor/facility*
- *Sexual health services*
- *Social services*
- *Clinical/medical services*
- *Legal/judicial*
- *Other, specify:* *(Local Language)* ________ *(English)* 12 ACLfothx

3 Type of contact:

13 ACLtype

- *In person*
- *Telephone*
- *Email/Internet*
- *Text message/SMS*
- *Other, specify:* *(Local Language)* ________ *(English)* 14 ACLtothx

VersionInitDate [1]

Version 2 15 16 VersDt | 17 formlang | Completed by: 18 InitDate *(initials/date)*

(HPTN 074) DF/Net 074 (CDF) 330

Visit Code: 6 visit

Participant ID: 7 ptid

Site Number | Network Number | Chk | Cohort

Initial Specimen Collection Date: 8 Date

dd MMM yy

Header [1]

CD4+ Count and Viral Load

CD4Count [1]

1 **CD4 + Count:** 9 CDFtcln *Not done/ Not collected*

Alternate Collection Date: 10 CDFtcldt (dd MMM yy)

1a. Absolute CD4+: 11 CDFcd4n *Unable to analyze* 12 CDFcd4 *cells/mm3*

2 **HIV-1 RNA PCR:** 13 CDFhrpn *Not done/ Not collected*

Alternate Collection Date: 14 CDFhrpdt (dd MMM yy)

2a. HIV RNA PCR (plasma): < = > 15 CDFhrp 16 CDFhrp *viral copies/mL* OR 17 CDFundct *Undetectable*

2b. RNA PCR kit code: 18 CDFrkit *kit code*

Comments (*Local language*): ______

Comments (*English*): 19 CDFcomm

Version 20, 25 21 VersDt | 22 formlang | VersionInitDate [1] Completed by: 23 InitDate *(initials/date)*

(HPTN 074) DF/Net 074 (AE) 460

Page: 6 visit

Log [1]

42 AEsaeno

Participant ID: 39 subsum

7 ptid

Site Number | Network Number | Chk | Cohort

Date Reported to Site: 8 Date

dd MMM yy

Header [1]

Adverse Experience Log

17 AEmdlth 10 AEmdlth | 15 AEmdlltc | 16 AEmdve

14 AEmdsoc | 11 AEmdpt

12 AEm

13 AEm

1 9 AEdiag

34 AEmquery

2 Onset date: 18 AEondt

dd MMM yy

CQC [1]

40 AEcqc3

3 Severity Grade: 19 AEseve Grade 5—death

ening

41 AEcqc4

4 Relationship to study product: 20 AErel Not related *If not related, record rationale or alternative etiology in Comments.*

5 Study product administration: 21 AEdrad N/A

6 Status or Outcome of AE: 22 AEoutc

Continuing

Resolved

Death

Severity/frequency increased ***(Report as new AE)***

Continuing at end of study participation

6a. Status/Outcome Date *(Leave blank if Status/Outcome is "continuing".)*

23 AEocdt

dd MMM yy

7 Treatment: *Mark "none" or all that apply.*

24 AEtrno *None* | 25 AEtrrx *Medication(s)* | 26 AEtrpr *Procedure/surgery* ***Comment below.***

27 AEtrho *New/prolonged hospitalization* ***Comment below.*** | 28 AEtrot *Other, specify:* ***Comment below.*** 29 AEtrotx

8 Is this an SAE according to ICH guidelines? 30 AEich No

9 Has or will this AE be reported as an EAE? (if a SUSAR)? 31 AEdaids No

10 At which visit was this AE first reported? *Visit code required (regular or interim).* 32 AEvisit visit code

Comments *(Local language):* ____________________

Comments *(English):* 33 AEcomm

VersionInitDate [1]

Version 1 35 36 VersDt | 37 formlang | Completed by: 38 InitDate *(initials/date)*

(HPTN 074) DF/Net 074 (MV) 463

Visit Code: 6 visit

Participant ID: 7 ptid

Site Number | Network Number | Chk | Cohort

Form Completion Date: 8 Date

dd MMM yy

Header [1]

Missed Visit

MissedVisit [1]

1 Target Visit Date: 9 MVtdt

dd MMM yy

2 Reason visit was missed. *Mark only one.*

10 MVreasn

- 2a. Unable to contact participant
- 2b. Unable to schedule appointment(s) within window
- 2c. Participant refused visit
- 2d. Participant incarcerated
- 2e. Participant admitted to a health care facility
- 2f. Participant withdrew from the study → ***Complete Termination form.***
- 2g. Participant deceased → ***Complete Termination form. Complete Adverse Event Log.***
- 2h. Participant travelled outside the province for work
- 2i. Other, specify: *(Local language)* ______

(English) 11 MVotrex

Comments (*Local language*): ______

Comments (*English*): 12 MVcomm

VersionInitDate [1]

Version 1 13 VerNum 14 VersDt | 15 formlang | **Completed by:** 16 InitDate *(initials/date)*

(HPTN 074) DF/Net 074 PIL (487)

Page 6 visit

Participant ID:

7 ptid

Site Number | Network Number | Chk | Cohort

FOR INTERNAL USE ONLY

Participant Incident Log

PIL [1]
Header [1]

Form completion date (dd MMM yy): 8 Date

Study Project Manager: 9 PILstaff

If applicable — Plate #: 10 PILplate — Visit code/page #: 11 PILvisit

Incident *Mark all that apply.*

- 12 PILinelg inappropriate enrollment/ineligible
- 13 PILrand randomization
- 14 PILhivt HIV testing
- 15 PILprot protocol deviation/event
- 16 PILsubo other, specify: 17 PILsubox
- 18 PILprots [protocol-specific reason]

Details: *Provide additional information, including date(s), site(s), lab(s), outcome, etc. as appropriate.*

19 PILevnt

VersionInitDate [1]

Version 20 21 VersDt

FOR INTERNAL USE ONLY

(HPTN 074) DF/Net 074 (TM) 490

6 visit

Participant ID:

7 ptid

Site Number | Network Number | Chk | Cohort

Header [1]

Termination

Termination [1]

1 Termination Date: 8 TMtmdt (dd MMM yy)

Date the site determined that the participant was no longer in the study.

2 Reason for termination. *Mark only one.*

9 TMtrmrsn

2a. Scheduled exit visit/end of study → ***End of form.***

2b. Death (Indicate date and cause if known.)

2b1. Date of death: 10 TMddt (dd MMM yy) OR 11 TMddun date unknown

2b2. Cause of death: 12 TMdcau OR 13 TMdcun cause unknown

→ ***Complete or update Adverse Experience Log.***

2c. Participant refused further participation, specify: 14 TMrefrx

2d. NOT APPLICABLE FOR THIS PROTOCOL

2e. Participant relocated, no follow-up planned

2f. Investigator decision, specify 15 TMinvdx

2g. Unable to contact participant

2h. NOT APPLICABLE FOR THIS PROTOCOL

2i. Inappropriate enrollment → ***End of form.***

2j. Invalid ID due to duplicate screening/enrollment → ***End of form.***

2k. Other, specify 16 TMtrmox

2l. Early study closure → ***End of form.***

2m. Index Termination (Network Partners only) → ***End of form.***

3 Was termination associated with an adverse experience? *yes* *no* *don't know* 17 TMae → ***If no or don't know, end of form.***

3a. Record AE Log page number: *page #* 18 TMaelp *OR* Specify: *(Local language)* *(English)* 19 TMreacx

Comments *(Local language)*: ____

Comments *(English)*: 20 TMcomm

VersionInitDate [1]

Version 21 22 VersDt | 23 formlang | **Completed by:** 24 InitDate *(initials/date)*

(HPTN 074) DF/Net 074 (PDL) 495

Page: 6 visit

Participant ID: 7 ptid

Site Number Network Number Chk Cohort

Form Completion Date: 8 Date

dd MMM yy

Header [1]

Protocol Deviation Log

ProtocolDevLog [1

1	Site awareness date: 9 PDLsdt (dd MMM yy)	2	Deviation date: 10 PDLddt (dd MMM yy)	
3	Has or will this deviation be reported to local IRB/EC?	11 PDLirb	No	
4	Has or will this deviation be reported to DAIDS as a critical event?	12 PDLdaids	No	
5	Type of deviation:	13 PDLtype	deviation code *(See back of form for code listing.)*	

6 Description of deviation *(Local language):*

Description of deviation *(English):* 14 PDLpdx

7 Plans and/or action taken to address the deviation *(Local language):*

Plans and/or action taken to address the deviation *(English):* 15 PDLdaddx

8 Plans and/or action taken to prevent future occurrences of the deviation *(Local language):*

16 PDLfdocx

9 Deviation reported by (staff name): 17 PDLStaff

VersionInitDate [1

Version 18 19 VersDt 20 formlang Completed by: 21 InitDate *(initials/date)*
